# Mouse α-synuclein fibrils are structurally and functionally distinct from human fibrils associated with Lewy body diseases

**DOI:** 10.1126/sciadv.adq3539

**Published:** 2024-11-01

**Authors:** Arpine Sokratian, Ye Zhou, Meltem Tatli, Kevin J. Burbidge, Enquan Xu, Elizabeth Viverette, Sonia Donzelli, Addison M. Duda, Yuan Yuan, Huizhong Li, Samuel Strader, Nirali Patel, Lauren Shiell, Tuyana Malankhanova, Olivia Chen, Joseph R. Mazzulli, Lalith Perera, Henning Stahlberg, Mario Borgnia, Alberto Bartesaghi, Hilal A. Lashuel, Andrew B. West

**Affiliations:** ^1^ Duke Center for Neurodegeneration Research, Department of Pharmacology and Cancer Biology, Duke University, Durham, NC 27710, USA.; ^2^ Aligning Science Across Parkinson’s (ASAP) Collaborative Research Network, Chevy Chase, MD 20815, USA.; ^3^ Department of Computer Science, Duke University, Durham, NC 27708, USA.; ^4^ Laboratory of Biological Electron Microscopy, Institute of Physics, School of Basic Sciences, Ecole Polytechnique Fédérale de Lausanne, and Department of Fundamental Microbiology, Faculty of Biology and Medicine, University of Lausanne, CH-1015 Lausanne, Switzerland.; ^5^ Department of Neurology, Northwestern University Feinberg School of Medicine, Chicago, IL 60611, USA.; ^6^ Department of Health and Human Services, Genome Integrity and Structural Biology Laboratory, National Institute of Environmental Health Sciences, National Institutes of Health, Durham, NC 27709, USA.; ^7^ Laboratory of Molecular and Chemical Biology of Neurodegeneration, Institute of Bioengineering, School of Life Sciences, École Polytechnique Fédérale de Lausanne, Lausanne, Switzerland.; ^8^ Department of Chemistry, Duke University, Durham, NC 27708, USA.; ^9^ Department of Biochemistry, Duke University School of Medicine, Durham, NC 27705, USA.; ^10^ Department of Electrical and Computer Engineering, Duke University, Durham, NC 27708, USA.; ^11^ Qatar Foundation ND BioSciences, Qatar Foundation Headquarters, PO Box 3400, Al Rayyan, Qatar.

## Abstract

The intricate process of α-synuclein aggregation and fibrillization holds pivotal roles in Parkinson’s disease (PD) and multiple system atrophy (MSA). While mouse α-synuclein can fibrillize in vitro, whether these fibrils commonly used in research to induce this process or form can reproduce structures in the human brain remains unknown. Here, we report the first atomic structure of mouse α-synuclein fibrils, which was solved in parallel by two independent teams. The structure shows striking similarity to MSA-amplified and PD-associated E46K fibrils. However, mouse α-synuclein fibrils display altered packing arrangements, reduced hydrophobicity, and heightened fragmentation sensitivity and evoke only weak immunological responses. Furthermore, mouse α-synuclein fibrils exhibit exacerbated pathological spread in neurons and humanized α-synuclein mice. These findings provide critical insights into the structural underpinnings of α-synuclein pathogenicity and emphasize a need to reassess the role of mouse α-synuclein fibrils in the development of related diagnostic probes and therapeutic interventions.

## Teaser

Commonly used mouse α-synuclein fibrils are structurally and functionally close to human E46K-mutated fibrils.

## INTRODUCTION

The accumulation of aggregated and fibrillar forms of α-synuclein (α-syn) in neurons is a defining hallmark of several neurodegenerative diseases that include Parkinson’s disease (PD), multiple system atrophy (MSA), and dementia with Lewy bodies (DLB), which are collectively referred to as synucleinopathies (*1*, *2*). Recent advances in cryo–electron microscopy (EM) have enabled unprecedented insight into the structural features of α-syn fibrils from different synucleinopathies, revealing disease-specific fibril structural features (*3*–*6*). Furthermore, in vitro recombinant α-syn can self-assemble into fibrils with distinct structural features, depending on the buffer composition, pH, and incubation parameters (*7*, *8*). Altogether, these observations demonstrate that α-syn is capable of forming a diversity of fibril types. However, our understanding of the molecular and cellular determinants of fibril formation and structure in the brain remains incomplete. Increasing evidence from studies investigating the pathogenic properties of recombinant and brain-derived α-syn fibrils suggests that the molecular architecture of folds and stacking arrangements within the fibrils, as well as their biochemical properties (e.g., posttranslational modifications), are key determinants of their pathogenicity and spreading patterns (*6*, *9*, *10*). The fibril strain hypothesis postulates that these differences may contribute to the neuropathological and clinical heterogeneity of PD and other synucleinopathies (*3*, *4*, *11*, *12*).

The formation and propagation of α-syn pathology occurs through recruitment of nonfibrillar (e.g., monomeric) α-syn protein into preformed or newborn fibrils in a self-propagation mechanism (*13*–*15*). α-Syn fibril formation is driven by extensive hydrogen bonding interactions involving a large segment of the protein that is highly structured in forming the core of the fibrils (*3*, *16*, *17*). Different α-syn fibrils have been described with remarkable variations of strand folding, rotational symmetry, and helical twists, as characterized by cryo-EM structure analysis. Fibrils have been purified for structural analysis directly from postmortem brain tissues in the presence of detergents, or generated detergent-free through recombinant α-syn protein incubated with patient biofluids and tissues in seeding aggregation assays, or produced using recombinant proteins under spontaneous aggregation reactions (*3*, *4*, *18*–*20*). Most known α-syn fibrils produced under physiological-like conditions consist of two protofilaments connected by salt bridges and intermolecular hydrophobic interactions.

Recent analyses of disease-associated ex vivo α-syn fibrils extracted from MSA brains (i.e., MSA-fold fibrils) revealed fibrils with core structures that are distinct from those found in PD, PD dementia (PDD), and DLB (*3*, *4*). Sarkosyl-insoluble α-syn fibrils extracted from MSA brain homogenates contain two types of distinct structures, each consisting of two asymmetric protofilaments each with 9 to 12 β strands (*3*, *21*). In contrast, the proposed Lewy fold associated with PD and DLB is composed of a single protofilament containing nine β strands, with a presumed salt bridge (E35-K80) involved in the compact packaging of the singular subunit (*4*). Attempts to replicate the structures of brain-derived α-syn fibrils in vitro by manipulating the aggregation conditions of recombinant α-syn proteins, or the use of brain-derived fibrils as seeds, have not yet been widely successful (*22*–*24*). For example, the structure of recombinant α-syn fibrils amplified from seeding with MSA-fold fibrils was different from all synucleinopathy ex vivo fibril variants. Fibrils seeded from MSA fold type II, referred to as MSA-amplified, resemble recombinant α-syn fibrils with the PD and DLB-associated E46K mutation in *SNCA* (*23*, *25*). Despite the structural heterogeneity, many types of recombinant α-syn fibrils have been consistently shown to exhibit high seeding efficiency when added to cells and primary neuronal cultures, or injected into the brain of rodent and nonhuman primate models of α-syn pathology formation and spreading (*13*–*15*, *26*–*28*). These properties of preformed fibrils (PFFs) enabled the induction of the aggregation, fibrillization, and formation of Lewy body–like inclusions in the absence of α-syn overexpression (*26*, *28*–*34*). This has led to the emergence of α-syn PFF seeding–based models as the most commonly used tools to investigate the mechanisms of α-syn pathology formation and spreading and to validate experimental therapeutic targets or anti–α-syn aggregation therapeutic strategies.

Different types of α-syn fibrils have been used as PFF seeds based on wild-type (WT) human and mouse recombinant α-syn as the major PFFs used by most laboratories. However, human α-syn fibrils, whether recombinantly expressed or purified from human tissues, poorly seed pathology in rodents and fail to produce progressive dopaminergic neurodegeneration when injected into WT mice and rats (*27*, *35*–*38*). In contrast, mouse α-syn fibrils exhibit higher seeding activity and induce more pathological spreading in rodents (*27*, *36*, *38*). For this reason, the injection of recombinant mouse α-syn fibrils is commonly used in both mice and rat studies. Mouse PFFs induce a sporadic Lewy body disease phenotype, including progressive dopaminergic neurodegeneration in combination with α-syn inclusion formations (*13*, *39*–*46*). Mouse α-syn fibrils bind thioflavin T (ThT) less compared to human fibrils and exhibit distinct morphological features, suggesting the possible presence of different structural conformations (*35*, *47*, *48*). The primary sequence of mouse α-syn differs from the human ortholog in seven amino acid positions (*49*, *50*). Several reports have demonstrated that a single substitution of mouse α-syn in the non-amyloid component (NAC) or pre-NAC domain (i.e., T53A or N87S) is sufficient for mouse α-syn to template human α-syn monomeric protein in vitro to the same extent as WT human α-syn (*35*, *47*, *51*). Solid-state nuclear magnetic resonance (NMR) studies identified probable structural differences between mouse and human α-syn fibrils but did not identify the nature of these differences (*52*). In complement, introduction of the S87N mutation into human α-syn increases the levels of α-syn seed-induced pathology in primary mouse hippocampal neurons (*35*). However, the sequence, molecular, and structural factors underlying differences in morphological and seeding properties of human and mouse α-syn fibrils have remained poorly understood.

Therefore, it has remained unclear whether the structure of mouse fibrils commonly used in preclinical models of PD and synucleinopathies matches to known recombinant or brain-derived human α-syn fibril structures. This knowledge gap has important implications for the field as the mouse PFF seeding models are increasingly used to decipher the role of α-syn in the pathogenesis of PD to test and validate targets and therapies, often with the assumption that the mouse fibrils form structures that resemble those formed by human α-syn. To address this knowledge gap, we sought to determine the structure of mouse and human α-syn fibrils using cryo-EM under identical conditions. Our results show that mouse α-syn fibrils are composed of a single homogeneous and reproducible fibril type that bears a strong similarity to human α-syn recombinant fibrils templated from MSA fold type II tissues and E46K-mutated fibrils. The functional properties associated with mouse α-syn fibrils are also strikingly different from human α-syn fibrils in nearly all pathological endpoints that were measured. Our findings provide important structural insights that explain the high seeding efficiency of mouse α-syn PFFs in mouse and human neurons and rodents and underscore the critical importance of developing disease-relevant human α-syn–expressing models of pathology formation and spreading. Models with better structural and functional overlap with disease seem achievable and essential to decipher disease-relevant pathogenic processes that increase the probability of success in translating findings from preclinical models to the clinic.

## RESULTS

### Mouse α-syn forms a core fibril structure distinct from both recombinant and brain-derived human α-syn fibrils

**Fig. 1.**
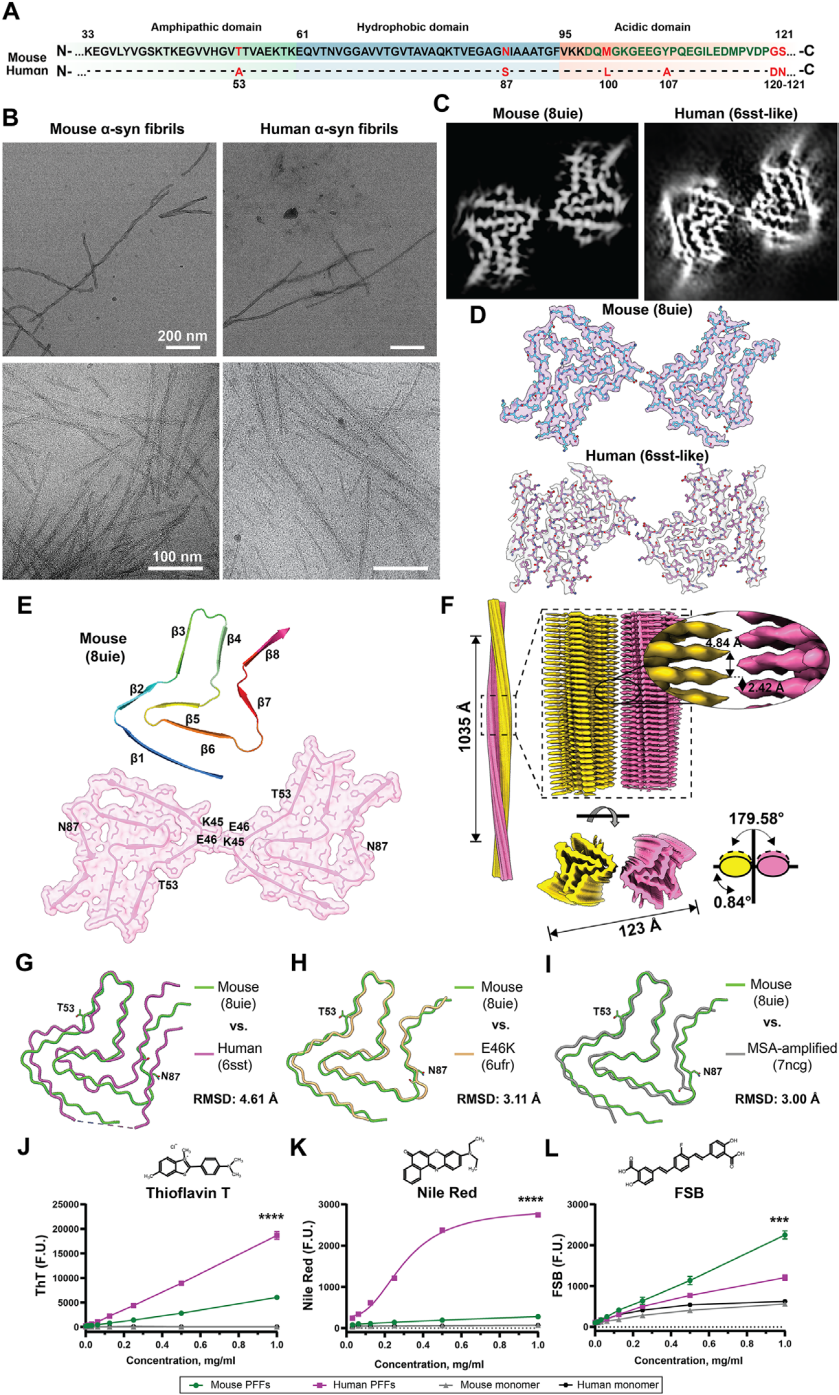
Mouse α-syn fibrils are structurally similar to human E46K-mutated and MSA-amplified α-syn fibrils. (**A**) Primary amino acid sequence comparing mouse and human α-syn, with indicated divergent residues highlighted in red. Depicted three domains are amphipathic in green, hydrophobic in blue, and acidic in orange. (**B**) Representative TEM (top row) and cryo-EM (bottom row) micrographs of mouse and human α-syn fibrils. (**C**) Central slice of the 3D cryo-EM map of mouse and human α-syn fibrils. (**D**) Cryo-EM density map surfaces of α-syn fibril single filaments. (**E**) Cartoon depiction of the fitted atomic model of mouse α-syn structural arrangement with β-strand positions and a cross-sectional view of the cryo-EM density map with an overlaid molecular model representation of the 3D density surface rendering of the fibril core. Highlighted are residues involved in the formation of a salt bridge between protofilaments (K45-E46) and residues that differ between mouse and human α-syn sequence. (**F**) Side view of the density map of mouse α-syn left-handed helices with a crossover distance of 1035 Å and a twist of −0.84°. The densities of the two intertwining protofilaments are colored yellow and magenta. An overlay of one layer of mouse α-syn (8uie, green) with (**G**) recombinant human (6sst, magenta), (**H**) recombinant E46K (6ufr, yellow), and (**I**) MSA-amplified (7ncg, gray) structures with visualized T53 and N87 amino acid schematics and indicated aligned total RMSD values. Alignments shown are with respect to fibril core-based best fitting (see table S2). Chemical structure and binding curves of ThT (**J**), Nile Red (**K**), and FSB (**L**) to mouse and human sonicated α-syn fibrils (average radii: 16.68 ± 1.44 nm and 15.14 ± 4.02 nm, respectively). F.U., fluorescence units. Data points indicate means from three independent experiments, and error bars are SEM. ****P < 0.0001 and ***P < 0.01 from two-tailed *t* tests.

To investigate and compare the structural properties of mouse and human α-syn fibrils, we purified both recombinant WT mouse and human α-syn proteins (Fig. 1A) from *Escherichia coli* and induced their aggregation under conditions [endotoxin-free phosphate-buffered saline (PBS), pH 7.4] that have been optimized and standardized for the production of recombinant α-syn fibril preparations across different laboratories (*15*, *44*). In both mouse and human α-syn fibril preparations, cryo-EM and transmission electron microscopy (TEM) micrographs revealed homogeneous unbranched structures with twisted morphology (Fig. 1B). Two-dimensional (2D) class averages reveal a single dominant structural class for both mouse and human α-syn fibrils (Table 1 and table S1). Helical reconstructions resolved a 3.1-Å map for mouse α-syn and a 2.7-Å map for human α-syn fibrils produced under the same conditions. Cross-sectional projections of both fibrils showed two protofilament architectures (Fig. 1C). Human α-syn formed fibrils with a core structure that is very similar to that of the previously reported 6sst human WT α-syn conformation (*18*), whereas the high-resolution fitted atomic model for mouse α-syn revealed a very different fibril core structure forming antiparallel pairs (Fig. 1, D to F). Mouse α-syn fibrils display a left-handed internal pseudo-C2 symmetry with protofilaments connected through a salt bridge formed by residues K45 and E46 (Fig. 1E). The monomer subunits stack along the fibril axis with a helical rise of 4.84-Å and helical twist of 0.84° (Fig. 1F). No other fibril structures were identified in either mouse or human α-syn fibril preparations.

**Table 1.** Statistics of cryo-EM data collection and structure refinement of mouse α-syn fibril preparations.

Fibril type	Mouse (Duke)	Mouse (EPFL)
Data collection		
Microscope	FEI Titan Krios	Thermo Fisher Scientific Glacios
Image detector	Gatan K3 (6k × 4k)	Gatan K3 (6k × 4k)
Pixel size (Å)	1.08	0.878
Defocus range (μm)	−2.5 to −0.8	−2.5 to −0.8
Voltage (kV)	300	200
Exposure time (s/movie)	8	1
Number of frames	55	30
Total dose (e^−^/Å^2^)	55	50
Micrographs	3322	5358
Interbox distance (pixels)	27	16
Initially extracted fragments	468,092	271,844
Segments after 2D classification	42,967	146, 075
Reconstruction		
Initial model used (PDB code)	6ufr	7ncg
Segments after 3D classification	27,524	50, 378
Resolution after 3D refinement (Å)	3.3	3.0
Final resolution (Å)	3.1	2.9
Estimated map sharpening *B*-factor (Å^2^)	−56	−52
Helical rise (Å)	2.42	2.39
Helical twist (°)	−0.84	−0.80
Atomic model		
EMDB code	EMD-42294	EMD-50023
PDB code	8uie	9ewv
FSC threshold	0.143	0.143
Composition		
Chains	12	12
Atoms (non-hydrogen)	5352	5676
Residues	768	768
*B*-factors (Å^2^)		
Protein	100	98.25
RMSDs		
Bond lengths (Å)	0.002	0.009
Bond angles (°)	0.434	0.918
Validation		
MolProbity score	1.44	1.74
Clashscore	8.10	7.25
Poor rotamers (%)	0	0
Ramachandran plot		
Favored (%)	98.39	95.16
Allowed (%)	1.61	4.84
Disallowed (%)	0	0

In an effort to substantiate these findings from mouse α-syn fibrils produced at Duke University, independent preparations of mouse α-syn fibrils (endotoxin-free PBS, pH 7.4) were also created and analyzed via cryo-EM analysis at the École Polytechnique Fédérale de Lausanne (EPFL). The conditions for generating mouse α-syn fibrils were standardized across both locations, with only minor variation in the concentration of monomeric protein (table S2 and Materials and Methods). Collected cryo-EM micrographs highlight unbranched homology similar to that observed from protein produced at Duke University (fig. S1A). Cross sections obtained at both sites were similar, further validating initial results (Fig. 1C and fig. S1B). The reconstructed cryo-EM map of the EPFL mouse α-syn (PDB: 9ewv) fibrils revealed the same structural characteristics as those observed at the Duke site (Fig. 1, E and F, and fig. S1, C to E). Although the collection and refinement methods at the two sites differed, including the use of different initial models (6ufr for Duke; 7ncg for EPFL), the consistency in the structures at atomic scales highlights the robustness and reproducibility of our findings (fig. S2, A to C, and Table 1). For clarity and distinction, we will refer to the 8uie mouse cryo-EM map as “Duke,” acknowledging its origin at Duke University, and the 9ewv map as ‘EPFL’, acknowledging its independent origin at the EPFL.

When comparing the mouse and human fibril structures, large shifts in the β strands are observed near areas where the protein sequences differ, specifically at residues T53 (A53 in humans) and N87 (S87 in humans; Fig. 1, A and G, and fig. S1D). Differences in the β-strand folding can be attributed to steric hindrance caused by N87, which is involved in forming an antiparallel β7 fold. In contrast, S87 might facilitate disruption of the β sheet into a loop. We noticed that the β-strand positioning associated with mouse fibrils overlaps with α-syn fibril structures previously reported for E46K-mutated α-syn (6ufr) as well as WT human α-syn fibrils amplified from MSA brain-derived fibrils [7ncg; Fig. 1, H and I; fig. S1, G and H; table S2; and (*23*, *25*)]. The alignment of mouse α-syn protofilaments in a global fit of five stacked fibril rungs demonstrates a particularly close alignment to the protofilaments obtained from human α-syn monomers seeded with MSA-amplified fibrils (fig. S3, A and B). However, the twist length is stretched between mouse fibrils (1035 Å) and MSA-amplified fibrils (900 Å), as well as a relaxed corresponding twist angle (0.84° versus 0.95°; fig. S3). Spontaneous WT human and PD-associated α-syn fibrils, or fibrils with posttranslational modifications, demonstrated incompatible alignments with mouse α-syn fibrils (figs. S4 and S5). These data show that mouse α-syn fibrils align with human MSA-amplified and E46K fibrils, but are different from human WT α-syn fibrils produced under commonly used experimental conditions. Neither mouse nor human fibril structures, produced under these conditions, aligned well with reported structures for sarkosyl-extracted α-syn fibrils from the brain [fig. S6 and (*23*)].

Notable differences in twist, angle, and β-fold patterning between mouse and human α-syn fibrils suggest the possibility of differential interaction with amyloid dyes that bind in pockets affected by these patterns, for example, ThT, Nile Red, and the Congo Red derivative dye FSB {1-fluoro-2,5-bis[(E)-3-carboxy-4-hydroxystyryl]benzene} that are commonly used in biomarker seeded aggregation assays and assessments of α-syn pathology in human brains and preclinical models of synucleinopathies (*53*–*55*). To test this prediction, both mouse and human α-syn fibrils were fragmented by sonication to uniform populations (i.e., PFFs) and then concentrations of particles were matched from multiple independent preparations (fig. S7). As anticipated, the concentration-dependent curves of ThT binding revealed a much stronger affinity for human as compared to mouse PFFs (Fig. 1J). Nile Red, a polarity-sensitive probe, revealed a large discrepancy between human and mouse α-syn PFFs (*56*, *57*). Nile Red binds poorly to mouse PFFs but strongly to human variant, suggesting that mouse α-syn fibrils do not have surface-exposed hydrophobic clusters (Fig. 1K). These observations are supported through FSB binding, a dye with two distinct hydrophilic groups which binds tighter to mouse than to human α-syn fibrils (Fig. 1L). To gain more insight into the structural basis of the differential binding of the amyloid dyes to human and mouse α-syn fibrils, we carried out molecular dynamic (MD) analysis under physiological conditions. We generated 2D hydrophobicity projections of generated cryo-EM maps that support the high surface hydrophobicity associated with human α-syn fibrils and low hydrophobicity associated with mouse α-syn fibrils (fig. S8A). In silico 3D models of electrostatic density showed a higher level of charged clusters in mouse α-syn fibrils, which appear to correspond to the hydrophobicity projections (fig. S8B). Altogether, these results demonstrate that human and mouse α-syn fibrils exhibit distinct structural, surface, and dye binding properties. The differential binding to dyes like Nile Red could be useful classifiers in lieu of high-resolution structures, as well as in seeding amplifications assays where differences in dye binding have emerged as one of the key distinguishing features of samples isolated from different synucleinopathies [i.e., in the amplification of fibrils from MSA versus PD colony-stimulating factor (CSF) samples (*53*)].

### Mouse α-syn fibrils exhibit increased fragmentation and sensitivity to detergents

Protein fibrils tend to be very stable in pathogenic forms, but can also show a great range of stabilities, presumably due to relatively minor structural changes that associate with disease in complex ways (*58*). Mouse α-syn fibrils are known to be very efficient in seeding de novo inclusions in neurons, but also readily fragment with sonication and may be poorly resistant to detergents (*59*–*61*). Several studies have shown that the length of α-syn fibrils is a strong determinant of α-syn seeding activity and pathology spreading in different models, with shorter fibrils (i.e., 20 to 100 nm) exhibiting the highest seeding activity (*46*, *62*, *63*). Fibril breakage increases the number of fibril ends, thus providing more surfaces to template the misfolding and aggregation of α-syn monomers. Furthermore, shorter fibrils are more likely to be internalized and secreted by neurons, which is expected to translate to increased seeding and spreading activities (*46*, *62*, *64*). Therefore, protein fibrils that are prone to fracturing may be more advantageous in spreading throughout a cell or across interconnected circuits in driving pathological properties, with fibril fragility linked to cellular cytotoxicity (*65*, *66*). The tendency of any amyloid fibril to fracture and partially denature can be influenced by the strength of β-sheet layer interactions across fibril rungs, further modified by backbone hydrogen bonding and hydrophobic interactions (*67*).

**Fig. 2.**
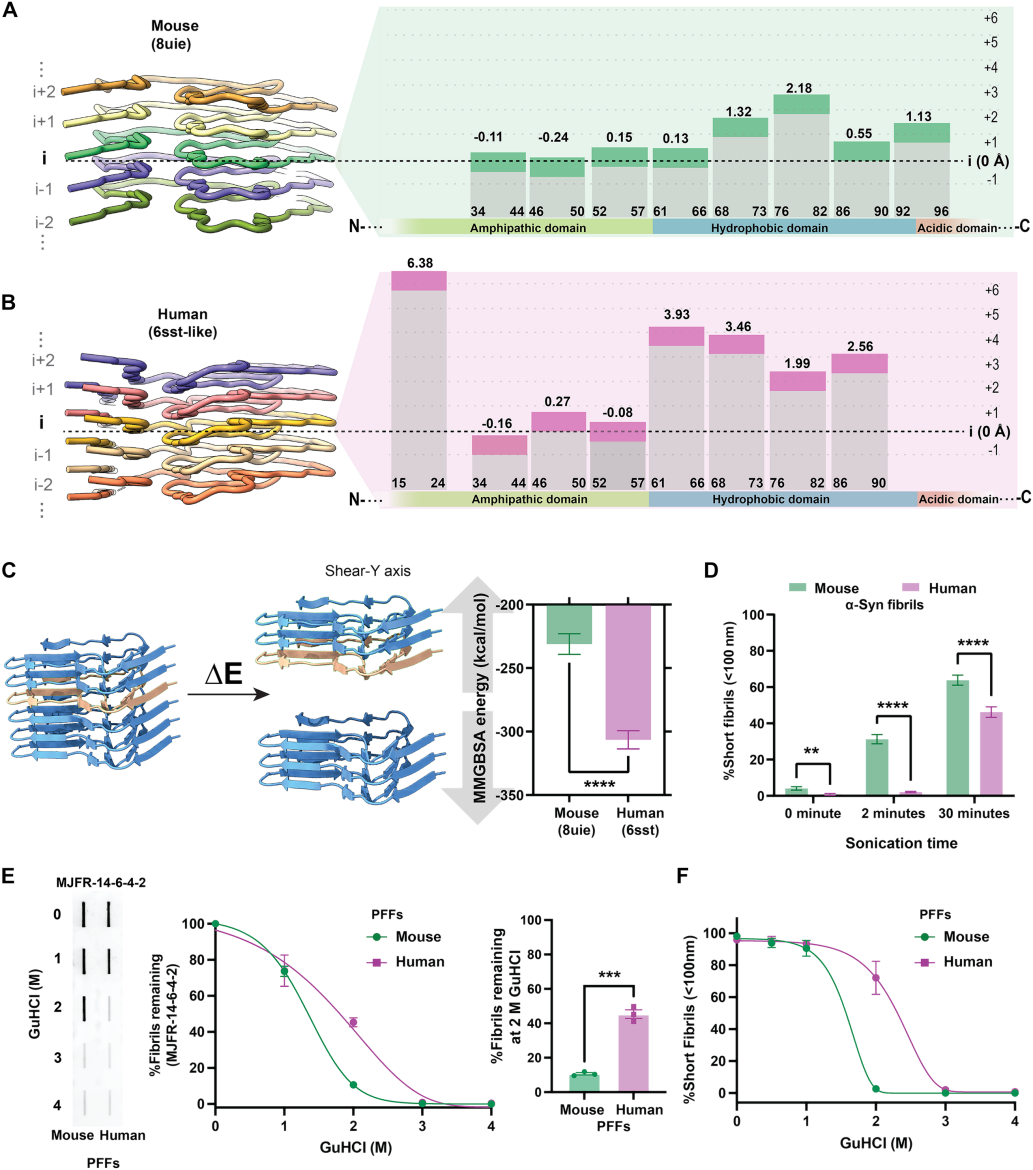
Distinct β-fold stacking arrangements in mouse α-syn fibrils contribute to low tensile strength and resilience. (**A**) Cross–β-structure of five protofilament rungs, and schematic representation of stacking arrangements with estimated coordinates against the assigned baseline position (i) as the horizontal plane (37 to 57 amino acids) mouse (8uie) and (**B**) human (6sst) cryo-EM models. (**C**) Representation of proposed model of fibril fragmentation for tensile strength estimation used to simulate the MMGBSA energy of α-syn fibrils rupture and group analysis of MMGBSA energy required to disrupt a stack of six rungs. Error bars represent SD from 100 independent simulations. (**D**) Group comparison of fibril breakage under sonication conditions shown as the percent of size population of 10 to 100 nm (e.g., short fibrils) at 0, 2, and 30 min, measured by DLS. Error bars indicate SEM of three independent experiments, with 30 acquisition measurements corresponding to each experiment. (**E**) Filter-trap slot-blot analysis of sonicated fibrils (i.e., PFFs) exposed to different concentrations of guanidinium chloride (GuHCl), and then remaining fibrils detected with the fibril-selective antibody MJFR-14-6-4-2. Error bars indicate SEM from three independent experiments. (**F**) DLS analysis of PFFs after incubation with GuHCl. Error bars indicate SEM of three independent experiments with 10 acquisition measurements for each biological sample. Curves in (E) and (F) show asymmetric sigmoidal models with a goodness of fit >0.96, and **P < 0.01, **P < 0.001, ****P < 0.0001 from unpaired two-tailed *t* tests.

We hypothesized that the increased seeding activity of mouse fibrils could also be due to their reduced stability and high tendency to fragment. To test this hypothesis, we examined potential differences in α-syn rung stacking using the high-resolution cryo-EM structures of both human and mouse α-syn fibrils. We noticed that mouse α-syn rungs were stacked flatly. The β sheets deviated from a horizontal plane more than 2 Å in height only between residues 76 and 82 in β-strand six (Fig. 2A). In contrast, human α-syn fibril rungs are kinked and rise higher along the horizontal plane in β strands from residues 61 to 90 (Fig. 2B). Next, we conducted a computational analysis to estimate the tensile strength required to break these rung interactions in a predicted fibril particle (Fig. 2C). The energy produced for mouse was 1.35-fold lower than that for human α-syn fibrils in the model. Decreased energy required to disrupt the mouse fibril layers implicates a higher sensitivity to stress fracturing. To test tensile strength of fibrils during sonication, we subjected full-length mouse and human α-syn fibrils to ultrasonic waves produced by a cup horn water bath–cooled apparatus that avoids probe-tip positioning effects, thereby minimizing heterogeneity in energies applied across the sample. Our and other previous studies have shown that the length of terminally sonicated mouse α-syn fibrils ranges from 15 to 25 nm under common sonication conditions in physiological buffers, while full-length intact fibrils are much longer and exceed well over 100 nm in length (*68*–*70*). Approximately 31.24% (±2.54) of mouse α-syn fibrils, compared to 2.08% (±0.41) of the human conformer, fracture to less than 100 nm in size after 2 min of sonication, suggesting that mouse α-syn fibrils are drastically less stable. When the sonication process was extended to 30 min, most of the mouse fibrils (64.5%, ±2.74) were fractured to less than 100 nm, whereas a larger proportion of longer fibrils persisted in the human fibril pool under these sonication conditions (53.77%, ±2.92; Fig. 2D).

Tensile strength is also linked to degradation, as fibrils that exhibit a low-force structural architecture are prone to rapid denaturation (*71*). To further assess the differential overall stability of mouse and human α-syn fibrils, we investigated their propensity to disassemble in the presence of detergents (*72*, *73*). This was achieved by monitoring the amount of remaining fibrils in the presence of increasing concentration of detergents. We conducted a filter-trap slot-blot analysis with previously fully sonicated fibrils (60 min, PFFs) and then incubated the PFFs with increasing concentrations of guanidine HCl or sarkosyl to assess the amount of detergent-resistant fibrils under denaturing conditions (Fig. 2E and fig. S9). While 2 M guanidine HCl was sufficient to denature mouse α-syn, human PFFs were more resistant to denaturation, as monitored using the MJFR-14-6-4-2 monoclonal antibody, which shows higher affinity and preferential binding to aggregated forms of α-syn. Monoclonal antibody MJFR-14-6-4-2 binding intensities were normalized to fibril preparations without detergents to account for the possibility that the MJFR-14-6-4-2 antibody might have different affinity between mouse and human α-syn PFFs. In agreement, percent of size populations attributed to sonicated fibrils rapidly shifted for mouse PFFs upon 2 M guanidine HCl denaturation compared to human variants monitored by light-scattering dynamics, confirming the susceptibility of mouse α-syn to detergents in an antibody-independent manner (Fig. 2F). Similar results were obtained with exposures to sarkosyl at concentrations previously used to extract brain-derived α-syn fibrils (fig. S9). Collectively, these results suggest that mouse α-syn fibrils are considerably more fragile and subject to rapid fragmentation even with low detergent concentrations.

### Mouse α-syn fibrils have low immunogenicity

**Fig. 3.**
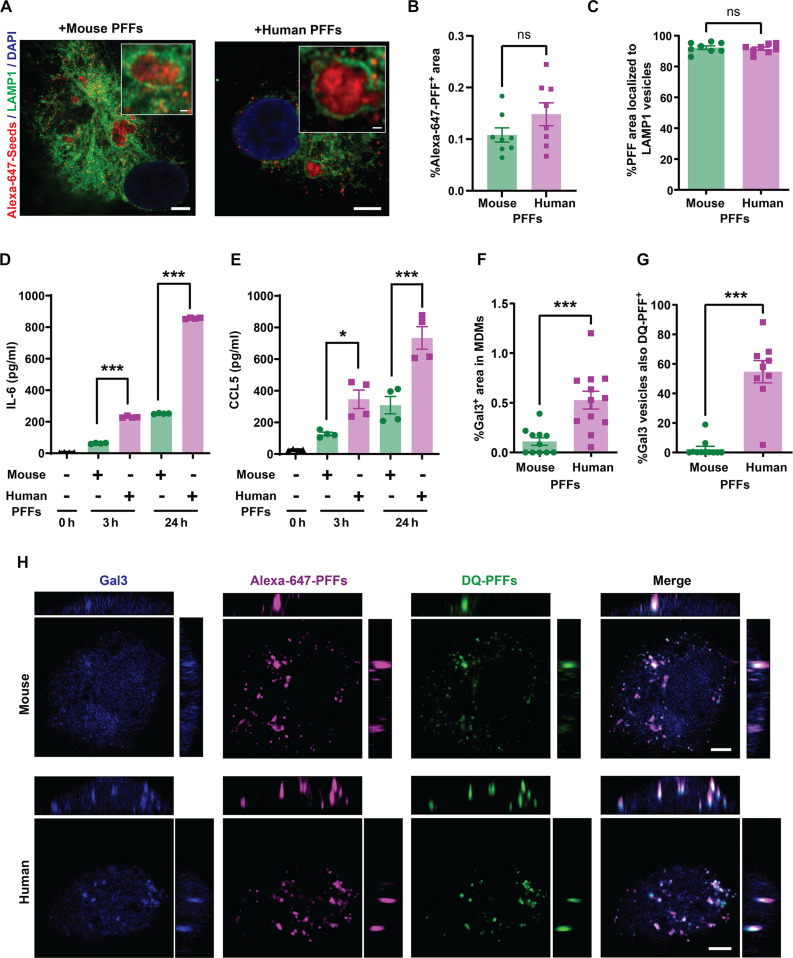
Mouse α-syn fibrils fail to elicit robust cytokine and lysosome damage in macrophages. (**A**) Representative orthogonal views of LAMP1 immunostaining in human MDMs after 2 hours of incubation with mouse or human α-syn PFFs (1 μg/ml). Magnified boxes highlight PFF-positive LAMP1 vesicles. Scale bars, 5 μm and 0.5 μm. (**B**) Internalized Alexa Fluor 647–PFFs (% area) inside cells and (**C**) percent of LAMP1-positive vesicles positive for PFFs. Each data point represents the means of cells analyzed from at least eight images from three independent experiments. (**D**) ELISA analysis of the extracellular IL-6 and (**E**) CCL5 from MDM cultures treated with PFFs (1 μg/ml) for 3 and 24 hours. Each data point represents the mean from two technical replicates from four independent experiments. (**F**) Percent of Gal3-positive vesicles calculated per mm^2^ of cell surface area in PFF-treated MDM cultures after 24 hours of incubation and (**G**) percent of Gal3-positive vesicles also positive for DQ-PFFs after 48 hours of PFF incubation. Data points show the mean values from cells imaged across three independent experiments with at least eight images analyzed per group. (**H**) Representative images of Gal3 immunostaining after 24 hours of treatment with Alexa Fluor 647– or DQ-labeled PFFs. Orthogonal views of sequential z-stacks are shown. Side left image = *x*,*y* plane, side right image = *y*,*z* plane; top image = *x*,*z* plane. Scale bar, 5 μm. Error bars represent SEM and ***P < 0.001, *P < 0.05, and ns for not significant from unpaired two-tailed *t* tests (B, C, F, and G) or from Tukey’s post hoc test after analysis of variance (ANOVA) (D and E).

Previous studies have suggested that different human α-syn fibril structures can elicit powerful immunological responses, especially from myeloid cells like microglia and monocyte-derived macrophages [MDMs; (*74*, *75*)]. Given these differences in the structural characteristics observed between human and mouse α-syn fibrils, we examined whether these structural differences might affect their functional interactions with immune cells. We cultured primary human MDMs from the blood of healthy individuals as previously described (*76*). Macrophages were first incubated with Alexa Fluor 657–conjugated mouse and human α-syn PFFs to determine whether there were differences in cellular uptake. Both mouse and human α-syn fibrils were derived from preparations of protein processed extensively through endotoxin removal columns, and verified endotoxin free (i.e., <0.1 endotoxin units per milligram of PFFs) via assessments with ultrasensitive limulus amebocyte lysate (LAL) assays. As mouse fibril preparations may present with an average shorter fibril length owing to spontaneous fracturing, immediately before treatment on cells, both mouse and human fibril preparations were sonicated to terminal lengths and particles matched in concentration between preparations (fig. S7). The uptake of both types of PFFs into macrophages was identical, with both fibril types equivalently localized into LAMP1-positive vesicles soon after addition to cell cultures (Fig. 3, A to C). To investigate the inflammatory responses to the PFFs, secreted human interleukin-6 (IL-6) and C-C motif chemokine ligand 5 (CCL5), known to be robustly stimulated by human α-syn fibrils, were measured by enzyme-linked immunosorbent assay (ELISA) in a time-dependent manner similar to methods previously described (*74*). With the addition of equivalent numbers of mouse and human fibril particles to the culture medium, human PFFs provoked a larger IL-6 and CCL5 response than mouse PFFs (Fig. 3, D and E). IL-6 and CCL5 signaling is associated with autophagy and lysosomal damage (*77*), and α-syn fibrils are thought to cause lysosomal damage in different types of cells (*78*). We noticed that internalized human PFFs tended to be co-positive in vesicles for the lysosomal damage marker Gal3, whereas few instances of internalized mouse fibril particles were associated with Gal3-positive vesicles (Fig. 3F). Gal3 recruitment to lysosomes has been associated with release of IL-6 upon CCL5 activation (*79*, *80*). Further analysis of colocalization with dye-quenched (DQ) mouse or human PFFs, produced as previously described to assess fibril-induced lysosomal damage, demonstrated few instances of Gal3 staining in degradative vesicles filled with mouse α-syn compared to most vesicles filled with human PFFs that show signs of damage [Fig. 3, G and H; (*81*)]. These results suggest that human, but not mouse, α-syn PFFs damage lysosomes and cause correlated cytokine and chemokine responses. Consistently, a previous study has shown that mixtures of E46K human α-syn fibrils, now known to bear resemblance to fibril structures associated with mouse α-syn, demonstrate reduced accumulations of an mCherry-Gal3 reporter on lysosomes in neuroblastoma cell lines and reduced vesicle rupture (*82*). The findings from this study point directly to differences in α-syn fibril structure as a key determinant affecting proinflammatory immunological responses likely through lysosome disruption.

### Mouse α-syn fibrils are subject to clathrin-dependent endocytosis and robustly cross-seed human α-syn pathology in neurons

**Fig. 4.**
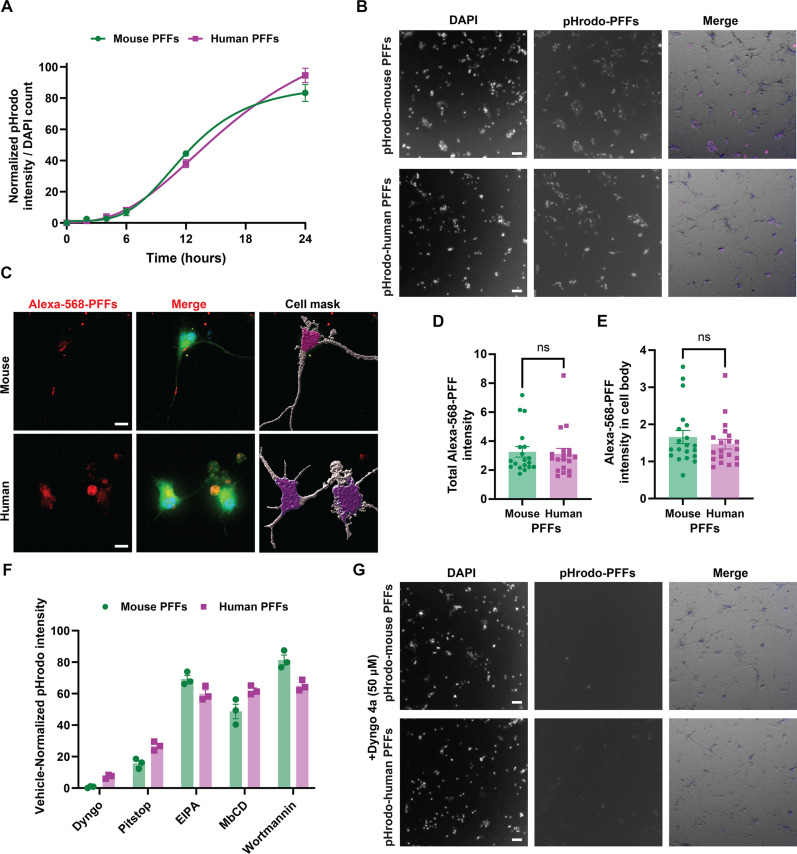
Mouse and human α-syn fibril uptake in neurons is similar and clathrin dependent. (**A**) Time-dependent dynamics of pHrodo-labeled mouse or human PFF (1 μg/ml) internalization over 24 hours into human-PAC-wt-*SNCA*^+/+^/*Snca*^−/−^ hippocampal primary neuronal cultures at DIV7, with normalized pHrodo-channel intensity to DAPI counts at the indicated time point. Each dot represents mean values from four images each from three independent neuronal cultures. (**B**) Representative immunofluorescence images of pHrodo-labeled PFF internalization at 24 hours. Merged images incorporate overlays of pHrodo-labeled PFF signal colored in magenta, DAPI in blue, and phase contrast in gray. (**C**) Representative images of ~60-day-old iPSC midbrain dopaminergic neurons treated with Alexa Fluor 568–labeled mouse or human PFFs at a concentration of 10 μg/ml. Merged images include Celltag labeling of total cell membrane in green, Alexa Fluor 568–labeled PFFs in red, and DAPI in blue. Owing to the high concentration of labeled fibrils used on iPSC-derived neurons, extracellular signals were quenched using 0.1% trypan blue. Cell masks highlight perinuclear and neuritic areas used for analysis. (**D**) Alexa Fluor 568 intensity in dopaminergic neurons and (**E**) intensity exclusively in the perinuclear area 8 hours after PFF incubation. Each dot represents the mean value of one image with at least 20 images collected from three independent experiments. (**F**) Calculated uptake of pHrodo-labeled α-syn PFFs at 24 hours in the presence of endocytosis inhibitors with signals normalized to vehicle only controls and (**G**) representative images. Merged images include the pHrodo-PFF signal indicated in magenta, DAPI in blue, and phase contrast in gray. Each dot in (F) represents the mean value of four images evaluated per condition from three independent experiments. Error bars for each group analysis represent SEM, and ns is not significant from two-tailed *t* tests.

α-Syn fibrils are known to be rapidly internalized in cells through a variety of reported endocytosis mechanisms that may vary between cell types (*83*). The effects of α-syn structural variation, such as that which exists between human and mouse α-syn fibrils, has not been closely evaluated on the uptake of fibrils into neurons. Differences in uptake between fibril structures could dictate downstream phenotypes such as inclusion formation and toxicity. To evaluate mouse fibril uptake, primary hippocampal neurons were cultured from human-PAC-wt-*SNCA*^+/+^/*Snca*^−/−^ transgenic mice that exclusively express the human α-syn protein at physiological levels (*68*), and low concentrations of terminally sonicated PFFs (1 μg/ml) were added to the cultures at day in vitro 7 (DIV7). Similar to macrophages, in both primary neurons and induced pluripotent stem cell (iPSC)–derived neurons, there were no apparent differences in neuronal uptake between human and mouse fibrils labeled with pHrodo and Alexa Fluor 568 as monitored over a 24-hour period (Fig. 4, A to E). Application of the clathrin-mediated endocytosis inhibitor Dyngo 4a, 30 min before addition of the labeled PFFs, virtually eliminated any traces of mouse or human fibril uptake, whereas the 5-(*N*-ethyl-*N*-isopropyl) amiloride (EIPA) small-molecule pinocytosis inhibitor had minor effects (Fig. 4, F and G). These results suggest that the uptake of sonicated α-syn fibrils into neurons in culture is similar between mouse and human fibrils in a clathrin-dependent endocytosis mechanism.

**Fig. 5.**
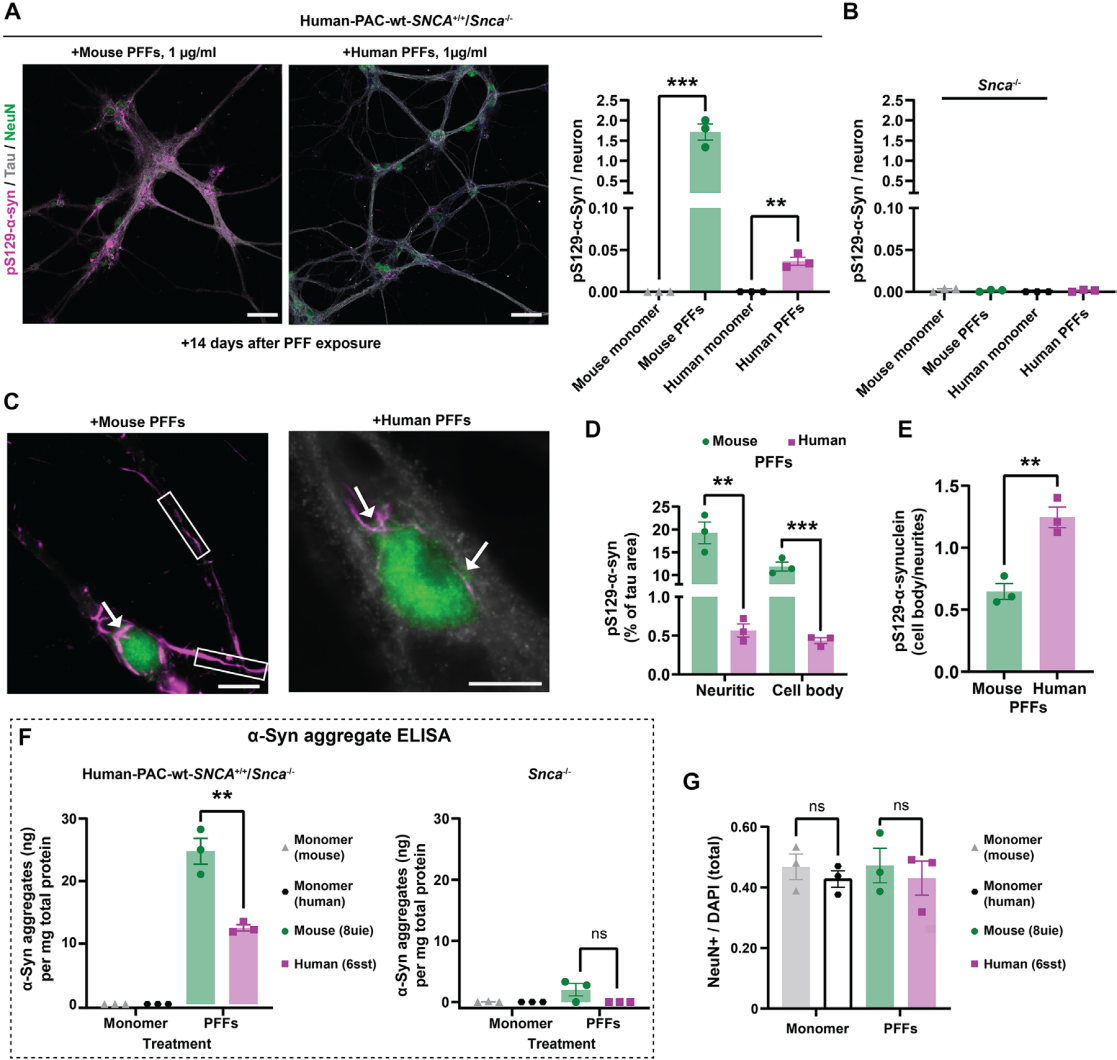
Mouse α-syn fibril pathology propagation is more efficient than human α-syn in primary neurons. (**A**) Representative immunostaining of neurons cultured from human-PAC-wt-*SNCA*^+/+^/*Snca*^−/−^ hippocampal primary neuron culture treated with α-syn PFFs (1 μg/ml) for 14 days, or equivalent amounts of monomer as indicated, and stained against pS129–α-syn (magenta), Tau (gray), and NeuN (green), with levels of pS129–α-syn signal assessed relative to the number of neurons in the corresponding cultures. (**B**) To ensure the specificity of pS129–α-syn signals, control groups show the lack of signal in neurons cultured from *Snca*^−/−^ mice following 14 days of incubation with α-syn PFFs or monomer as shown. (**C**) Representative images of inclusions in the cell bodies (indicated with arrows) and neurites (outlined in square boxes) in human-PAC-wt-*SNCA*^+/+^/*Snca*^−/−^ neurons 14 days after PFF exposure. Scale bar, 50 μm and 10 μm (magnified images). (**D**) Abundance of distinct pS129–α-syn signals in cell bodies or neuritic morphology in neuronal cells treated with mouse or human α-syn PFFs (1 μg/ml). (**E**) Proportion of pS129–α-syn occupancy in cell body and neurites in primary hippocampal cultures incubated with mouse or human α-syn PFFs for 14 days. (**F**) ELISA quantification of α-syn aggregate levels in cell lysates from human-PAC-wt-*SNCA*^+/+^/*Snca*^−/−^ or *Snca*^−/−^ neuronal cultures treated with fibril PFFs or monomeric protein for 14 days. (**G**) Group analysis of NeuN-positive nuclei abundance normalized to DAPI count. Each data point in a group in the graphs represents the mean of signal from an individual litter with two technical replicates per litter and at least 25 images analyzed for each replicate, with error bars indicating SEM. Significance was determined by two-tailed *t* tests: **P < 0.001, ****P < 0.0001.

With similar uptake between mouse and human fibrils, since human α-syn fibrils poorly template endogenous mouse α-syn protein (*35*), it can be reasonably predicted that mouse α-syn fibrils would also exhibit a marked reduction in templating human α-syn monomers. However, different structural and mechanical fibril properties discovered here between mouse and human fibrils that include reduced hydrophobicity and elevated rates of fragmentation may affect seeding potency in neurons. Therefore, we directly compared the seeding activity of mouse and human fibril preparations, paying close attention to equivalency in the number of fibril particles applied to cell culture models. Mouse PFFs were much more efficient in seeding human α-syn pathology compared to human PFFs (Fig. 5A). Staining of neuronal cultures derived at the same time from *Snca*^−/−^ transgenic mice, which do not express α-syn protein, showed no evidence of pS129–α-syn pathology when treated with the same concentration of α-syn PFFs or control monomeric α-syn (Fig. 5B and fig. S10). Observing the pS129–α-syn patterning in neurons, there is a tendency for human PFF-seeded pathology to localize near the cell nucleus, whereas mouse α-syn fibril pathology spreads across the cell (Fig. 5, C to E). Other studies have also indicated that a higher proportion of cell body–like, or somatic, inclusion can be associated with a Lewy body functional fibril profile, different from those found with MSA-associated fibrils that appear to spread pathology across the cell (*84*). These functional results further corroborate an MSA-like fibril phenotype associated with mouse α-syn fibrils.

To further assess the extent of pathology formation, we used an antibody-based ELISA approach designed to quantify aggregated, but not monomeric, forms of α-syn. The ELISA approach is extremely sensitive in detecting both mouse and human α-syn fibrils without apparent bias to picogram levels (fig. S11). In total lysates from neurons, we found that mouse fibrils induce ~2.5 times higher levels of α-syn aggregates compared to equivalent concentrations of human fibril particles (Fig. 5F). With this technique, we did not detect any signal above background in neuronal lysates from samples incubated with the same amount of monomeric α-syn protein. Further, in cultures from *Snca*^−/−^ mice (that lack any endogenous α-syn expression), incubation with either mouse or human α-syn fibrils did not yield any detectable signal after 14 days of incubation, demonstrating that the ELISA signals obtained do not reflect the fibril particles (i.e., PFFs) added to the cultures, ostensibly due to their degradation in the culture over time in the absence of endogenous α-syn. In this experimental paradigm, there was no loss of neurons that would otherwise confound results, as assessed through NeuN cell and DAPI (4′,6-diamidino-2-phenylindole)–positive counts in the cultures (Fig. 5G).

Observing the unexpected phenomenon that mouse α-syn PFFs can template more pathology in mouse neurons expressing only human α-syn, we sought to confirm these results in the context of human iPSC-derived dopaminergic neurons (iPSC-DA) prepared and cultured for 60 days by established protocol (*85*). Characterization of neuronal and dopaminergic markers showed that cultures contained 81% (±4.1) FOXA2/TH + neurons and 90% (±5%) TH/βIII-tubulin + neurons (fig. S12A). iPSC neurons incubated with equivalent concentrations of Alexa Fluor 568 mouse and human PFFs for 7 days revealed the presence of puncta pS129–α-syn across the cell body and neurites. Notably, Alexa Fluor 568 mouse α-syn fibril particles provoked more than twice the pathology than comparable particles of human PFFs (fig. S12, B and C). Also similar to observations in mouse neurons, Alexa Fluor 568 mouse PFF-induced pS129–α-syn pathology tended to spread across the neuron through the neurite, whereas human α-syn fibril-induced pathology tended to occur predominantly in the cell body (fig. S12D). Overall, these results demonstrate higher seeding potency of mouse PFFs and suggest that they are able to form diffuse pathology across neuronal processes and cell bodies could be linked to their higher tendency to fragment and diffuse in neurons. Despite the structural and surface differences between the mouse and human fibril particles that include large differences in hydrophobicity, uptake remains the same in neurons through a similar clathrin-dependent endocytosis pathway.

### Mouse α-syn fibrils demonstrate exacerbated spreading in humanized α-syn mice

**Fig. 6.**
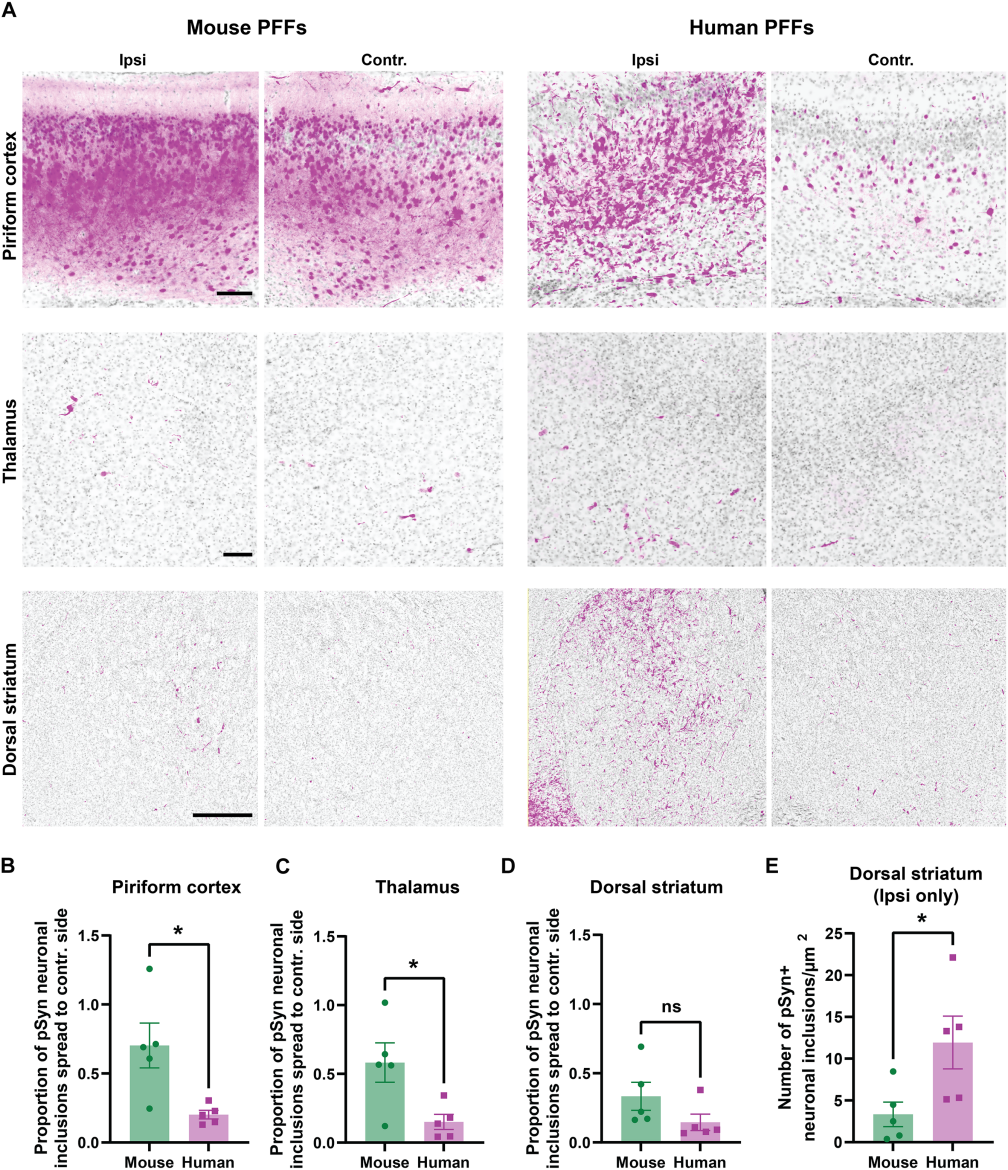
Mouse PFF induced α-syn pathology spreads through the mouse brain to seed human α-syn pathology more efficiently than human α-syn PFFs. (**A**) Representative immunofluorescence images of pS129–α-syn pathology (magenta) and DAPI (gray) 3 months after PFF injection into human α-syn–expressing SNCA-OVX mice (lacking *Snca* expression). Matched (size and concentration) PFFs (10 μg) were unilaterally injected into the dorsal striatum. Analysis of pS129–α-syn pathology in the piriform cortex (top row), thalamus (middle row), and dorsal striatum (bottom row) is separated to indicate ipsilateral (Ipsi) and contralateral (Contr.) sides. Scale bars, 100 μm (top and middle rows) and 500 μm (bottom row). (**B**) Group analysis of α-syn pathology propagation ratio to contralateral side in piriform cortex, (**C**) thalamus, and (**D**) dorsal striatum, quantified as proportion of pSyn neuronal inclusion spread between ipsilateral and contralateral areas. (**E**) Analysis of pS129–α-syn pathology near the injection site within the ipsilateral dorsal striatum. Each data point (*n* = 5 per group) in group analysis plots represents the mean of the signal from 20 to 25 sections from an individual animal, and error bars indicate SEM. Significance was determined by two-tailed *t* tests: *P < 0.05.

To explore whether the unique functional features of mouse α-syn fibrils discovered in primary neurons translate to the mouse brain, we selected SNCA-OVX transgenic mice that express human α-syn throughout their brain without expression of endogenous mouse *Snca*. We administered single intracranial injections into the dorsal striatum, using equal quantities of mouse or human PFFs alongside mice injected with saline (vehicle) control. Three months after injection, a time when inclusions are described as most abundant with mouse PFF injections (*13*, *86*, *87*), we stained the extracted brains against pS129–α-syn to investigate the spread and pathology abundance across the brain. We studied the propagation of α-syn pathology in several brain regions, comparing the ipsilateral and contralateral hemispheres (Fig. 6A). As anticipated, both human and mouse PFF variants triggered the formation of pS129–α-syn in the ipsilateral hemisphere. The vehicle control injection, however, did not induce any α-syn–associated pathology, consistent with our past reports with this method (*88*). To further explore the transmission of α-syn pathology via interneuronal connectivity, we measured the spread of α-syn inclusions to the contralateral side. Our findings revealed that mouse α-syn PFF spread more effectively from the injection site to the contralateral side in the piriform cortex, thalamus, and contralateral striatum (Fig. 6, B to D). This result could not be explained by more pathological abundance at the injection site, since human PFFs caused more pathology in the ipsilateral dorsal striatum (i.e., around the injection site) than mouse PFFs (Fig. 6E).

**Fig. 7.**
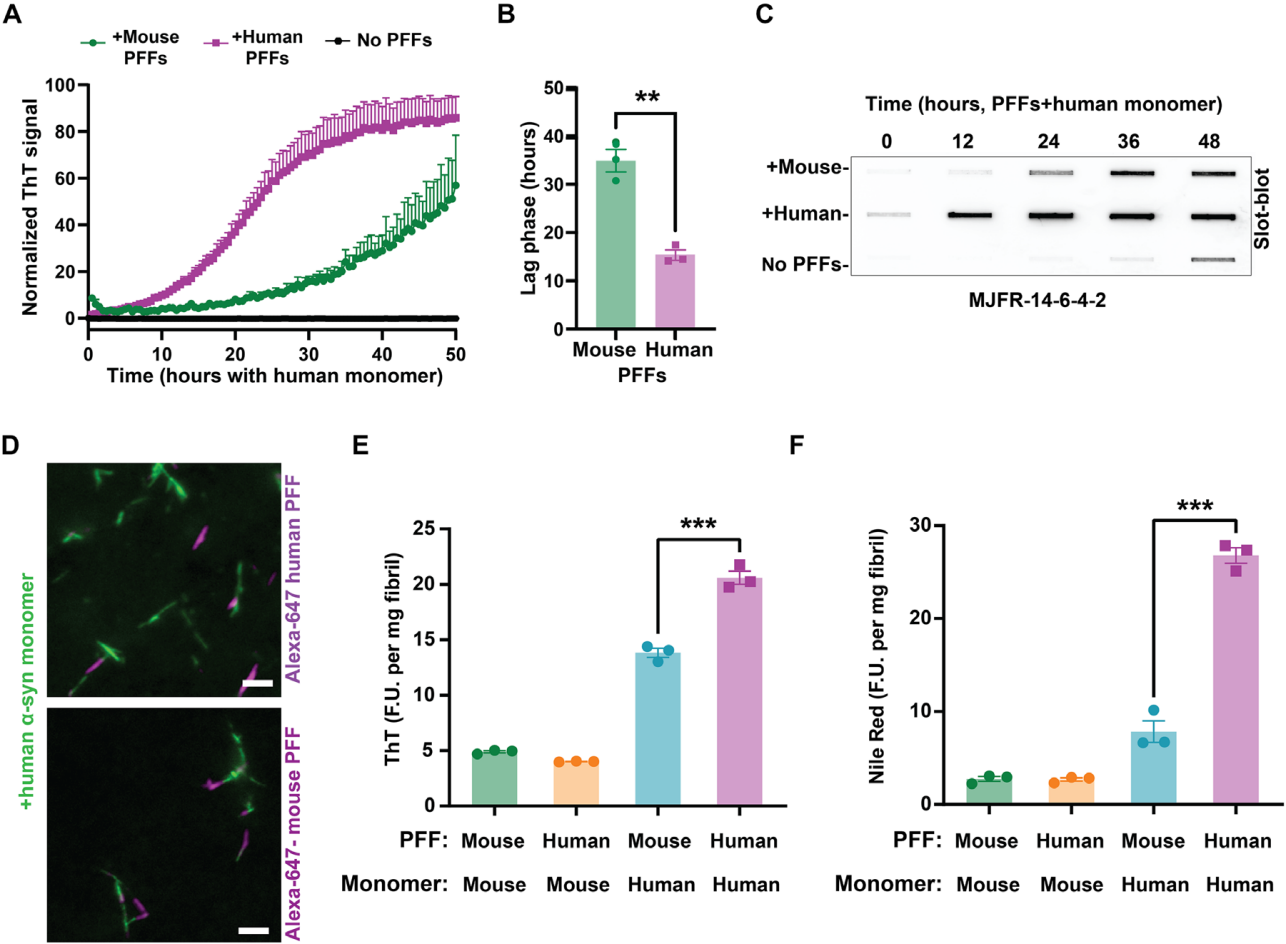
Recruitment of human α-syn monomer into mouse PFFs leads to the generation of mouse-like fibrils. (**A**) Representative aggregation assays showing mouse and human PFF-templated aggregation with human α-syn monomer, with (**B**) the calculated lag phase. Data points represent normalized ThT fluorescence from three independent experiments, with error bars indicating SEM. (**C**) Representative filter-trap slot-blot membranes stained with MJFR-14-6-4-2 α-syn aggregate-specific antibodies for the detection of aggregated α-syn in corroboration of aggregation kinetics without amyloid dyes. (**D**) Representative images of labeled (Alexa Fluor 647, magenta) mouse or human α-syn PFF-templated aggregation with human α-syn monomer, collected after 48 hours of incubation. Resultant fibril products were stained with ThT (green) shown with an overlay of PFFs (magenta). Scale bars, 0.5 μm. (**E**) Group analysis of ThT binding from chimeric or homogeneous sequence–extracted, monomer-free, sonicated α-syn fibril (PFF) products, as well as (**F**) Nile Red binding. Each data point in (E) and (F) represents a mean from an individual experiment measured in duplicate with three independent experiments. Error bars indicate SEM, **P < 0.01 from a two-tailed *t* test, and ***P < 0.001 from Tukey’s post hoc test after ANOVA.

In vitro studies with mouse α-syn fibrils have demonstrated that they poorly template human α-syn protein into cross-seeded fibrils (*35*). Given the unexpected results here in both primary neurons and in vivo with neurons expressing only human α-syn, we assessed again the cross-seeding properties of human and mouse α-syn fibrils using in vitro aggregation kinetics assays. Time-resolved aggregation assays were designed with normalized ThT signals (i.e., percent of maximum signal) to account for the difference in ThT binding between mouse and human fibrils. Consistent with previous observations, in vitro aggregation assays demonstrated that human PFFs are much more potent than mouse PFFs in templating human monomeric α-syn into new fibrils (Fig. 7, A and B). Mouse PFFs poorly template human α-syn monomer, in contrast to what we observed in neurons. To confirm these results without the use of any amyloid dye, we repeated the experiments with endpoints defined by reactivity measured through the aggregation-selective MJFR-14-6-4-2 monoclonal antibody (Fig. 7C), which did not appear to have species preference between human and mouse α-syn fibrils. Using cross-labeled fibrils, microscopic inspection of the chimeric fibrils confirmed that both mouse and human PFFs are incorporated into single-growing fibrils harboring both mouse and human α-syn protein (Fig. 7D). Thioflavin-binding profiles of the chimeric products suggest that neither human nor mouse α-syn PFFs completely preserve parental fibril structure into the newly born mixed (i.e., chimeric) fibrils (Fig. 7E). However, the mouse fibril PFFs recruit human monomers into new chimeric fibrils that have very low Nile Red staining, much closer to the starting mouse fibrils in comparison to typical human PFFs (Fig. 7F). In further evaluation of kinetic properties associated with the chimeric fibrils, aggregation assays with mouse monomer demonstrate that the chimeric fibrils (PFFs originating from mouse seeds that incorporated human monomer) largely recapitulated pure mouse α-syn fibril kinetics (fig. S13). Moreover, chimeric human PFFs (i.e., PFFs originating from human seeds that incorporated mouse monomer) showed decreased overall growth rates as expected for pure fibrils. Although high-resolution structures were not obtained from cross-seeded fibril structures, altogether, the derived dye profiles combined with kinetic behaviors suggest that mouse α-syn fibrils can incorporate human monomeric α-syn into mouse-like α-syn fibrils, despite the sequence differences between mouse and human α-syn. Further, the reverse appears to be true, with human fibrils recruiting mouse monomer protein into human-like fibrils. These results would suggest that the fibril structures seeded by mouse α-syn fibrils that form in neurons expressing human α-syn are likely similar to parental mouse fibril structures, although direct observations in intact neurons would be needed to confirm these predictions. Further, a better recognition of the specific cellular factors that drive de novo α-syn fibril formation and provide the initial constraints for fibril folding in neurons, which may not be the same between mouse and human α-syn, could be essential to enable the reproduction of structures of fibrils associated with Lewy body diseases. On the basis of these observations, we recommend exercising caution in the potential over-reliance on aggregation studies without relevant cell-based models.

## DISCUSSION

Mouse α-syn fibrils are commonly used for the discovery of molecules that might bind to high affinity and function as probes for disease features, or in drug discovery efforts to find therapeutics that block aggregation (*89*–*91*). It has been presumed that the structures and features associated with mouse α-syn are close enough to those associated with disease that they will inform pathways and targets that might be pursued to better understand disease. Cryo-EM analysis of protein fibrils associated with disease offers unprecedented insights into how conformational variation in fibril structure might drive different disease endpoints. Despite the ubiquitous integration of mouse α-syn fibrils into research models of α-syn pathology formation and spreading in PD and other synucleinopathies, there have been no high-resolution structures of these tools to inform whether they are similar or distinct from the growing compendium of α-syn fibril conformations found in different diseases or generated under various aggregation conditions in vitro. Addressing this knowledge gap has critical implications for assessing the utility of seeding-based mouse models, which rely primarily on the induction of aggregation and pathology formation by endogenous mouse α-syn, in translational PD research and drug discovery.

Here, we report the cryo-EM structure of mouse α-syn fibrils and demonstrate that it exhibits distinct structural features, amyloid dye binding, hydrophobicity profiles, and stability properties compared to human α-syn fibrils prepared under identical conditions. Structures and results were replicated in multiple laboratories. Although different buffer conditions in vitro are known to affect fibril structures, fortunately, the field has largely coalesced around standardized in vitro growth conditions for mouse α-syn fibrils (*44*) so that the structures and results produced in this study can be used to re-interpret past studies using these particles. The high-resolution structure of mouse α-syn reveals an arrangement clearly distinct from human α-syn fibrils produced under the same buffer conditions. Moreover, the high similarity of mouse α-syn fibril cryo-EM maps between independently generated and processed datasets at two different sites serves as a robust validation of the reproducibility inherent in our findings. The mouse α-syn fibril preparations were homogeneous and highly reproducible from one batch to the next, even between the two sites.

Notably, the structure of the mouse fibrils does not resemble any of the brain-derived α-syn fibril structures from patients with PD and other synucleinopathies. Unfortunately, mouse α-syn fibrils seem unstable in even low amounts of detergents like sarkosyl that are routinely used to help isolate protein fibrils from the brain or other tissues. Recently, a cryo–electron tomography (cryo-ET) method was disclosed for the in situ analysis of fibrils associated with tau and ß-amyloid, although here the tissues used were devoid of α-syn aggregates (*92*). Besides the analysis of high-pressure frozen human brain tissues, cryo-ET might be adopted to analyze neurons in culture in different models, and our group and others are actively working in these areas. We can predict that cryo-ET analysis of fibrils found in different α-syn models and human brain tissues affected by Lewy body diseases will be transformative in the interpretation of the results of this study as well as many others. The compendium of fibril structures analyzed here that considers nearly 50 different types of α-syn fibrils so far analyzed, together with their associated buffer and amplification conditions, stands ready to compare to newly derived cryo-ET structures. At least for recombinant α-syn fibrils currently in use, our results with cryo-EM are consistent with predictions made from a comparative solid-state NMR-based study comparing mouse and human α-syn fibrils (*52*), with the interface between the protofilaments of mouse α-syn fibrils found to be more hydrophilic, and the two types of fibrils different at residues T53 (A53) and N87 (S87).

The deposition of α-syn protein fibrils into larger aggregates such as Lewy bodies or neurites has been associated with strong immunogenic responses in affected tissues of several neurodegenerative diseases (*75*, *93*–*97*). While a broad comparative analysis of immunogenic potentials of different α-syn fibrils associated with disease has not been performed, to our knowledge, at least one study identified human α-syn fibrils as the most potent in inducing the secretion of proinflammatory cytokines and chemokines (*74*). The high expression of pattern recognition receptors like Toll-like receptor 4 (TLR4) is thought to drive these responses in the recognition of human α-syn fibrils presented at physiological picomolar concentrations (*98*). Here, we find that the more hydrophobic human 6sst α-syn fibrils are much more potent in eliciting an inflammatory response in macrophages, whereas mouse α-syn fibrils fail to cause a similar reaction. The Gal3 protein accumulates on impaired lysosomes and indicates the activation of autophagy-lysosomal pathways that might help eliminate hazardous molecules, including protein aggregates (*99*–*101*). The Gal3 protein acts as a TLR4 ligand to contribute to macrophage activation and IL-6 secretion (*102*). Although we did not pinpoint the mechanism of suppressed immune activation associated with mouse α-syn fibrils, it seems likely that the lack of lysosomal damage caused by the mouse fibrils before their elimination may cause a failure of Toll receptor signaling pathway and NLRP3 inflammasome activation that interconnects damaged lysosomes to the secretion of IL-6 and other cytokines in a variety of immune cells. Since we now know that mouse α-syn fibrils share structure with E46K-mutated human α-syn fibrils, these results may be compatible with a previous study demonstrating that E46K human fibrils fail to provoke lysosome damage and Gal3 accumulations on lysosomes in cell lines compared to WT human fibrils (*82*). Together, our findings suggest that the structural properties of the fibrils, and not only their biochemical properties, are key determinants of their interactions with lysosomes and pathogenicity (*87*).

While mouse PFF-induced inclusions in mouse brain strongly correlate with dopaminergic neurodegeneration in nontransgenic mice (*13*, *46*), our observation that mouse α-syn fibrils exhibit heightened fragmentation and increased sensitivity to detergents, as well as increased spread of pathology in neurons, may not be consistent with the general concept that fibril stability predicts pathogenicity (*58*). However, several studies have shown that the length of α-syn fibrils is a strong determinant of their seeding activity, with α-syn shorter fibrils exhibiting higher seeding activity (*46*, *62*, *63*). Fibril fragmentation outside of the degradative lysosome might be more advantageous for pathogenicity, since enhanced fragmentation is thought to promote the spread of newly seeded fibrils across the cell and across interconnected cells as well as increase the apparent number of seed-competent fibril ends (*58*, *65*). However, what causes fibrils to fragment remains unknown, and whether spontaneous breakage or cofactor induced snaps along the longer filaments. It seems likely either way that factors in the cell milieu critically determine the pathogenicity of α-syn fibrils (*103*). A recent study by Ohgita *et al.* (*51*) investigated the relative contribution of intramolecular interactions between the C- and N-terminal domains of α-syn within fibrils with respect to the stability of both mouse and human α-syn. Their results revealed that these intramolecular interactions are perturbed in mouse α-syn fibrils and could contribute to their higher tendency to fragment. Another study from our group showed that post-aggregation nitration induces α-syn fibril fragmentation in vitro (*104*). Further, MSA tissue–extracted α-syn fibrils show higher sensitivity to chemical degradation compared to PD-associated fibrils (*105*). Moreover, MSA fibrils are found to disassemble under lower concentrations of guanidine and exhibit higher pathology propagation potential compared to recombinant or PD-derived fibrils in vitro (*53*, *105*, *106*). Collectively, these results suggest that nuanced properties related to different aspects of fibril stability, among a myriad of other factors, may be critically important for determining α-syn–related pathogenicity and downstream disease phenotypes.

Our results underscore the importance of validating the structural similarities or differences of seeded aggregates to the native fibrils by cryo-EM before concluding successful amplification of native α-syn fibrils from brain or peripheral tissues. The field is increasingly relying on in vitro amplification assays to draw conclusions about the structural and pathogenic properties of native α-syn fibrils (*107*). Incorporating in vivo results, differential potencies between human and mouse α-syn fibrils vary considerably from one neuron type to another. It is clear through this study and others that both human and mouse α-syn PFFs associated with a variety of structures are available to cross-seed and potentially form new chimeric progeny structures that do not exactly resemble the parental seeding structures, but rather echo the major properties of the parental seeds. Again, paralleling our results with mouse α-syn fibrils to human E46K fibrils, a recent study demonstrated that E46K fibril seeds could recruit WT α-syn monomers into progeny fibrils that aligned well with the parental E46K fibrils despite the sequence differences, according to cryo-EM analysis (*108*). Had we performed a full cryo-EM analysis of species cross-seeded progeny fibrils here, we would expect to derive very similar results to those reported from similar experiments with E46K human fibrils, that the progeny fibrils with mismatched monomer protein end up structurally aligning with the parental seeds and not spontaneous fibrils that the monomer protein would otherwise form. We expect that this inherent property of protein fibril growth can be exploited to understand the structural diversity of potential soluble seeds associated with different Lewy body diseases. As seeding assays become closely incorporated into research pipelines and clinical trials, a better understanding of the types of fibril products formed in these reactions at the atomic level of detail could give rise into a better understanding of structural heterogeneity that may be important in disease.

Following the results disclosed here, several predictions for the interpretation of past studies using mouse α-syn fibrils can be made based on the structure-to-function observations in this study. First, immunogenic responses to mouse α-syn fibrils may be underestimated compared to fibril types associated with Lewy body diseases. Second, the spread of pathology and induction of de novo fibrils templated from mouse α-syn fibrils in neurons may be overestimated compared to human fibrils, if not assessed using quantitative biochemical methods for monitoring loss of monomers or formation of fibrils. These findings may help explain why mouse α-syn fibrils are very successful in templating pathology compared to fibrils extracted from PD and DLB, regardless of whether the models endogenously express human or mouse *SNCA/Snca*. Third, in vitro aggregation assays, especially those exclusively reliant on thioflavin binding, may not reflect important fibril properties that dictate pathological potency in cells that might include the effects of hydrophobicity and fragility differences between fibril types. Consistent with these observations and our findings, a recent study by Howe *et al.* (*69*) reported that mouse α-syn fibrils induced more pS129–α-syn pathology in rat brains than in rat α-syn fibrils, despite primary amino acid sequence differences, and suggested that additional cellular factors and not simply sequence homology between the fibrils and the monomeric subunits are critical determinants of α-syn fibril seeding activity and pathogenic properties.

In summary, the results from this study have shed additional light on structural and functional properties of α-syn fibrils that may drive their pathobiological characteristics, and highlight the importance of a detailed structural analysis of tools used in research models that are intended to recapitulate central neuropathological features in certain aspects of disease. A better characterization of the types of α-syn aggregates associated with disease, and methods to recapitulate them in models, may facilitate a higher success rate of pathological probes and therapeutics intended to mitigate the pathological effects of the aggregates in disease.

## MATERIALS AND METHODS

### Purification of recombinant human and mouse α-syn

Recombinant expression and purification of human and mouse α-syn were conducted by a previously published protocol (*68*). Plasmid (pET21a) constructs encoding human and mouse α-syn proteins were used to transform *E. coli* BL21-CodonPlus (DE3)-RIL competent cells (Agilent, catalog #230245-41). Protein expression at OD_600_ (optical density at 600 nm) was induced by adding 0.05 mM isopropyl-β-D-thiogalactopyranoside (IPTG) (RPI, catalog #I56000) at 18°C for 16 hours. The collected bacterial pellets were thoroughly resuspended in lysis buffer [10 mM tris-HCl, pH 7.6, 750 mM NaCl, 1 mM phenylmethylsulfonyl fluoride (PMSF), 1 mM EDTA, protease inhibitors (Sigma-Aldrich, catalog #4693132001)]. Resuspended homogenates were lysed by sonication for 1 min at 70% amplitude (Fisher500 Dismembrator, Fisher Inc.) and heating at 90°C for 15 min followed by centrifugation at 20,000*g* for 1 hour at 4°C. Supernatants were dialyzed against the dialysis buffer (10 mM tris-HCl, pH 7.6, 25 mM NaCl, 1 mM EDTA, 1 mM PMSF) overnight and purified through HiPrep Q HP 16/10 column (Cytiva, catalog #29018182) with linear gradient application of high-salt buffer. Identified fractions were selected based on the presence of the protein band at correct molecular weight, and the purity of the band was evaluated by SDS-PAGE (polyacrylamide gel electrophoresis). Selected fractions were combined and then dialyzed with the dialysis buffer (10 mM tris-HCl, pH 7.6, 25 mM NaCl) overnight. Anion exchange chromatography followed by dialysis was repeated to achieve a high purity sample. The dialyzed protein suspension was concentrated using ultracentrifugation units 3000 cutoff (Amicon, catalog #UFC901024) up to 5 ml. Finally, endotoxins were removed using an endotoxin removal kit (Genscript, catalog #89233-330), and the levels of endotoxin were measured using an endotoxin detection kit (Genscript, catalog #95045-024). A detailed protocol is available: dx.doi.org/10.17504/protocols.io.yxmvm3rz9l3p/v1.

### Preparation of mouse and human α-syn fibrils

Monomeric α-syn, at a concentration of 5 mg/ml in PBS, was incubated for 5 days with intermittent shaking (1 min on and off, Eppendorf, Thermomixer Model 5382, ThermoTop Smart Block catalog #5308000003) at 1000*g* at 37°C. Resulting suspensions were centrifuged at 20,000*g* at 10°C for 10 min. Soluble fraction was removed, and the remaining fibril pellets were washed three times in 1 ml of PBS. To measure fibril concentration, aliquots were titrated into 3 M guanidine chloride and agitated at room temperature for 5 min. Concentrations were then determined through *A*_280_ (absorbance at 280 nm) measurements using NanoDrop (Thermo Fisher Scientific, catalog #ND-ONE-W). In some experiments, fibril preparations were labeled with 100 μg of pHrodo iFL Red STP Ester (Thermo Fisher Scientific, catalog #P36010), Alexa Fluor 647 NHS Ester (Thermo Fisher Scientific, catalog #A20006), Alexa Fluor 568 NHS Ester (Thermo Fisher Scientific, catalog #A20003), and BODIPY-FL *N*-hydroxysuccinimide (NHS) ester (Thermo Fisher Scientific, catalog #D2184) at a concentration of 1 mg/ml in 1 ml of 100 mM sodium bicarbonate overnight with constant low-speed shaking at room temperature. Unconjugated dye was removed through rounds of pelleting the insoluble fractions via low-speed centrifugation and washing in PBS. For DQ fibrils, equal concentrations of Alexa Fluor 647– and BODIPY-FL–conjugated fibrils were mixed together before the cell treatment. Preceding the application as indicated, fibrils were sonicated to matched lengths of 20 to 30 nm in radius, with polydispersity indices <0.5 determined by dynamic light scattering (DLS) conducted on the same day of the experiment. The sonication of fibrils was achieved with a cup horn sonicator in thin-walled 0.2-ml tubes (Q-Sonica, Cup horn, catalog #431C2; Q700 Sonicator, catalog #Q700-110). A detailed protocol is available: dx.doi.org/10.17504/protocols.io.q26g7p268gwz/v1.

Fibrillization of chimeric fibrils was conducted by adding 1% (50 μg/ml) recombinant mouse/human α-syn PFFs (or Alexa Fluor 647–conjugated variants) to the sample containing mouse/human α-syn monomer (5 mg/ml) in PBS. The reactions for the evaluation of fibril growth were incubated for 5 days with intermittent shaking (1 min on and off, Eppendorf, Thermomixer Model 5382, ThermoTop Smart Block catalog #5308000003) at 1000*g* at 37°C. To monitor the elongation of added PFFs by incorporation of monomeric α-syn, the resulting reactions were supplemented with 50 μM ThT. After three wash cycles with PBS, the samples were further diluted to a concentration of 400 ng/ml and imaged on Zeiss Axio Observer Z1 using Plan-Apo oil immersion objective with ×100 magnification.

### Cryo-EM and helical reconstructions (Duke site)

Full-length recombinant mouse and human α-syn fibrils were applied to plasma-cleaned (Pie Scientific) 1.2/1.3 UltrAufoil (Quantifoil, catalog #Q350AR13A) grids using an automatic grid plunger (Leica GP2) set at 95% humidity and at 20°C. Before application to the grid, 4-μl aliquots of fibrils were thoroughly mixed. The mixed fibrils were then added to the grid, with the blot parameters set for a duration of 4 s for back-blotting. Subsequently, the grids were plunge frozen into a liquid Ethane/Propane mix. Images were captured on a Titan Krios cryo-TEM at a dose of 60 electrons per Å^2^, following the Latitude data collection procedures (Gatan Inc.). Before atlas collection, image alignments and fine alignments were executed with selected data collection parameters outlined in Table 1. Movie frames were gain corrected, aligned, and dose weighed using Relion 4.0 software (RRID:SCR_016274; OMICtools). Contrast transfer function (CTF) parameters were estimated using CTFFIND-4.1 software (RRID:SCR_016732; MRC Laboratory of Molecular Biology). For helical reconstructions, approximately 40 images were randomly selected for manual picking and around 14,000 fragments were extracted with an interparticle distance of six β rungs of 28.5 Å (with an estimated helical rise of 4.75 Å) from the start-end coordinate pairs. These extracted coordinates were used for training crYOLO (RRID:SCR_018392; Sphire). The same model was used for all image datasets in autopicking mode with crYOLO. Fragments were initially extracted using a box size of 1024 pixels and then down-scaled to 256 pixels, resulting in a pixel size of 4.32 Å. Reference-free 2D classifications were performed to assess different fibril strains, cross-over distances (estimated as 1200 Å), and helical rise (4.82 Å), and to select suitable fragments for further processing. Initial references were generated from selected 2D class averages using the Relion_helix_inimodel2d command with a cross-over distance of 1200 Å, search shift of 15 Å, and search angle of 15°. The 2D classes showing split protofilaments or protofilaments only were discarded. Another round of 2D classification was performed using a box size of 384 and no binning. The clean set of fragments achieved by selecting 2D classes showing clear rungs features was re-extracted using box sizes of 512 pixels and down-scaled to 256 pixels (binned pixel size of 2.16 Å) for optimal performance in 3D classifications. To minimize reference bias, initial models were low-pass filtered to 40 Å. During 3D classification, six to eight classes were used along with initial helical twists set to −0.7°, initial helical rises of 4.82 Å, and a regularization parameter *T* of 4. Helical symmetry local search was performed during classification, with helical twist ranges varying from −0.85 to −0.5 and helical rise ranges varying from 4.8 to 4.84. Only the central 10% of the *z* length was used for reconstructions. Classes with nominal folding features were individually selected and re-extracted with box sizes of 384 pixels. Additional rounds of 3D classification were performed with the new reconstructions as reference using an initial low-pass filter set to 10 Å to further clean the particles. Initial helical twist and helical rise parameters were updated from 3D classifications. Local symmetry search was not performed at the first round of refinement. In the second round refinement, a shape mask comprising 90% of the central *z* length was applied along with local symmetry search. To improve the resolution, multiple rounds of CTF refinement, Bayesian polishing, and refinements with previous results using a particle extraction size of 256 pixels were used. Postprocessing was accomplished using a shape mask comprising 10% of the central *z* length, and final resolutions were estimated for each dataset using the gold standard FSC 0.143 cutoff. The final twist and rise for mouse fibril were determined as −0.84 and 4.84, respectively. The final twist and rise for human spontaneous fibril were determined as −0.75° and 4.82 Å, respectively. More information about the processing is included in table S1. A detailed protocol is available: dx.doi.org/10.17504/protocols.io.ewov1qwy2gr2/v1.

### Cryo-EM and helical reconstructions (EPFL site)

Cryo-EM images were collected using a Thermo Fisher Scientific Glacios electron microscope, equipped with an X-FEG electron source and operated at 200 kV. A total of 5258 dose-fractionated movies were recorded with a Gatan K3 camera in counting mode at a physical pixel size of 0.8780 Å, a defocus ranging from 0.8 to 3 μm, and a total dose of ~50 e^−^/Å^2^, using SerialEM with life analysis using FOCUS (*109*, *110*). The movies were imported into RELION 4.0 for data processing (*111*, *112*). Motion correction was performed using RELION’s implementation of Motioncor2 (RRID:SCR_016499; University of California at San Francisco) with a dose of 1.6 e^−^/Å^2^ per frame (*113*). CTF correction was done using CTFFIND 4.1.14 (RRID:SCR_016732; MRC Laboratory of Molecular Biology), and 4904 selected micrographs were used for further image processing (*114*). From the 4904 micrographs with a maximum CTF resolution better than 5 Å and defocus ranging from 0.8 to 2.4 μm, 4144 fibrils were manually selected.

At first, 271,844 particles with a box size of 674 Å were extracted and subjected to reference-free 2D classification, leading to the selection of 146,075 particles, which were subsequently re-extracted with a box size of 316 Å (360 pixels) for 3D refinement. The initial model was created using IniModel2D from six distinct 2D averages, each having a box size of 674 Å, encompassing diverse views and a crossover distance of 1050 Å. After two rounds of 3D refinement, a third refinement was tested for C2 and pseudo-twofold symmetry. The structure with pseudo-twofold symmetry was implemented in 3D refinement and classifications. After multiple rounds of 3D classification and refinement (helical z-percentages between 0.15 and 0.3), a total of 50,378 particles with 316-Å box size underwent CTF refinement, Bayesian polishing, and postprocessing, yielding an optimized twist of 179.598° and a rise of 2.39 Å. Postprocessing was performed with a soft-edged mask and an estimated sharpening B-factor of −52 Å^2^, resulting in a 2.9-Å structure according to gold-standard Fourier shell correlation (FSC) = 0.143 criteria. Model building was performed manually for one rung of the single protofilament, using Coot 0.9.8.92 (RRID:SCR_014222; MRC Laboratory of Molecular Biology) (*115*). Both unsharpened and sharpened maps were used for modeling the peptide backbone. Further, rounds of iterative real-space refinement were carried out on six adjacent fibril stackings of the sharpened map using Phenix (phenix.real_space_refine) (RRID:SCR_014224; University of California; Berkeley) and ISOLDE, incorporating β-stacking secondary structure strains (*116*–*118*). The model quality was assessed using MolProbity (RRID:SCR_014226; Duke University) (*119*). The matchmaker function in UCSF ChimeraX (RRID:SCR_015872; University of California at San Francisco) was used to facilitate comparisons with previously published structures (*120*).

### Other structure alignments

Mouse α-syn structure (8uie) was superimposed and aligned with recombinant human (6sst), recombinant E46K (6ufr), and MSA-amplified (7ncg) in UCSF ChimeraX using the command “align” focusing on residue range 54 to 66, where the 3D feature indicated the most conserved among those structures (*121*). Cα root mean square deviation (RMSD) values were calculated based on the alignment with sequence range of 34 to 97. For global alignment, mouse α-syn fibrils (8uie), recombinant human (6sst), recombinant E46K (6ufr), and MSA-amplified (7ncg) were imported into Maestro (RRID:SCR_016748; Schrodinger Inc.) and prepared with the Protein Preparation Workflow task. Preprocessing was performed with the following parameters: fill in missing side chains; assign bond orders, using Combined Chemical Dictionary (CCD) database; replace hydrogens; create zero-order bonds to metals; create disulfide bonds; fill in missing loops using Prime (RRID:SCR_014887; Schrodinger Inc.); sample water orientations; use crystal symmetry; minimize hydrogens of altered species; and use PROPKA with pH 7.4; restrained minimization was then performed, converging heavy atoms to RMSD of 0.3 Å using OPLS4; waters were removed 3.0 Å beyond het groups. The prepared fibril structures were trimmed on their terminal ends to each contain five β-structure repeat units and amino acids 37 to 96 (600 residues total for each fibril). A single protofilament was isolated by removal of the paired protofilament in the fibril structure. Prepared protofilament structures were exported to Protein Data Bank (PDB) format for alignment. Protofilament structures were aligned using MM-align, and RMSD values were computed (*122*). MM-align computes alignments of multi-chain protein complexes. Aligned structures were exported from MM-align to PDB format.

### MD simulations

For the preparation of starting configurations of MD trajectories, which were based on the cryo-EM structures, missing atoms and protons were introduced by using the leap module of AMBER.20 (RRID:SCR_014230; University of California). Counter ions were added, and the systems were solvated in a box of water with the box boundary extending to 20 Å from the nearest peptide atom. Before equilibration, the solvated system was sequentially subjected to (i) 500-ps belly dynamics with fixed peptide, (ii) minimization, (iii) low-temperature constant pressure dynamics at fixed protein to assure a reasonable starting density, (iv) minimization, (v) stepwise heating MD at constant volume, and (vi) constant volume simulation for 10 ns with a constraint force constant of 10 kcal/mol applied only on backbone heavy atoms. After releasing all constraining forces, sampling was increased by performing four independent constant-temperature constant-volume MD simulations for 100 ns each. All trajectories were calculated using the PMEMD module of Amber.20 with 1-fs time step. The amino acid parameters were selected from the SB14ff force field of Amber.20, and configurations were applied for every nanosecond of the analysis.

### MMGBSA energy analysis

Using the Molecular Mechanics with Generalized Born and Surface Area (MMGBSA) protocol of Amber.20, interaction free energies of all the residues in the third protofilament with the residues in the two surrounding protofilament from each side were calculated for the 100 configurations extracted at each nanosecond interval from each trajectory. The ionic strength for MMGBSA calculations was selected to be 0.1 M.

### Amyloid dye binding assays

Fresh aliquots of 50 mM Nile Red (Sigma-Aldrich, catalog #19123-10MG), FSB (Sigma-Aldrich, catalog #344101-5MG), and 500 mM ThT (Sigma-Aldrich, catalog #BC85) in dimethyl sulfoxide (DMSO) were diluted in PBS to reach 100 μM concentration. Freshly sonicated α-syn PFFs evaluated by DLS were prepared at concentration 1 mg/ml followed by serial dilutions by a factor of 2. Diluted α-syn PFFs were combined with amyloid dye aliquots and transferred into 384-well plates with clear bottoms (Corning, catalog #4588). Fluorescence intensity was recorded on CLARIOstar OMEGA reader (BMG Inc.) with excitation/emission spectra set at 468-15/510-20 for fetal bovine serum (FBS), 535-20/585-30 for Nile Red, and 448-10/482-10 nm for ThT. Monomeric mouse and human α-syn monomers were used as a control. A detailed protocol is available: dx.doi.org/10.17504/protocols.io.x54v9yznpg3e/v1.

For real-time aggregation reactions, samples were supplemented with 10 μM ThT in ultralow binding 384-well plates with clear bottoms and sealed with foil (Bio-Rad, catalog #1814040). Each plate was supplemented with a standard curve of serial dilutions of recombinant α-syn reference PFFs. Reaction fluorescence was monitored at 448-10 nm excitation and 482-10 nm emission on FLUOstar Omega (BMG Inc.) plate reader every 30 min with intermittent shaking at 700*g*. A detailed protocol is available: dx.doi.org/10.17504/protocols.io.6qpvr67kpvmk/v1.

### Fibril fragmentation and denaturation analyses

Freshly prepared full-length α-syn fibrils were diluted to 5 mg/ml and aliquoted into 0.2-ml thin-wall polymerase chain reaction (PCR) tubes (BrandTech, catalog #13-882-58), each containing 20 μl of the sample. These aliquoted α-syn fibrils were then placed into a PCR tube adaptor (Qsonica, catalog #451) and subjected to ultrasonic waves using a cup horn water-bath sonicator (Qsonica, catalog #431C2). The sonication was performed for 2 min at 30% amplitude in a temperature-controlled environment set at 10°C. After sonication, the samples were collected for subsequent analysis. The sonicated fibrils were further diluted to 200 ng/ml and measured using the DLS approach, conducting 30 acquisitions for each sample. A separate set of freshly prepared full-length α-syn fibrils underwent sonication for 30 min. For the analysis, size distributions were divided into groups of sizes: 1 to 10 nm, 10 to 100 nm, >100 nm. Each sonication cycle included three technical replicates to account for the variability of the distribution of ultrasonic waves in the cup horn adaptor. A detailed protocol is available: dx.doi.org/10.17504/protocols.io.6qpvr3nebvmk/v1.

For the chemical denaturation analysis, sonicated fibrils were initially assessed using DLS to ensure that the size distributions matched between the mouse and human fibril preparations. Chemical denaturants were subjected to serial dilutions, with a dilution factor of 10 for SDS (Sigma-Aldrich, catalog #436143) starting from 10%, 2 for sarkosyl (*N*-lauroylsarcosine, Sigma-Aldrich, catalog #61739) starting from 2%, and guanidinium chloride (GuHCl) (Sigma-Aldrich, catalog #G3272) starting from 6 M concentrations. The sonicated fibrils were then diluted to a concentration of 2 mg/ml and spiked into the serial dilutions of the chemical denaturants. These reactions were incubated for 30 min and then diluted to 200 ng/ml before the slot-blot analysis.

### Slot-blot analysis

Samples of α-syn PFFs, at a concentration of 200 ng/ml, were applied to 0.22-μm nitrocellulose membrane, previously soaked in tris-buffered saline (TBS), using a bio-dot slot format microfiltration apparatus (Bio-Rad, catalog #1706542). Samples were filtered and washed in tris-buffered saline with 0.1% tween-20 detergent (TBST) through the membrane using a low-speed vacuum to ensure full drainage of the liquid. Washed membranes were removed from the cassette and placed in the blocking solution containing 5% (w/v) nonfat dry milk (Bio-Rad, catalog #1706404XTU) in TBST. The membrane was then transferred into a solution containing Alexa Fluor 647–MJFR-14-6-4-2 (Abcam, catalog #ab216309) antibodies for detection of α-syn–specific aggregates. A detailed protocol is available: dx.doi.org/10.17504/protocols.io.q26g7pkrkgwz/v1.

### MDM culture

Mononuclear cells from the blood of healthy volunteers were separated using SepMate and Lymphoprep tubes (STEMCELL Technologies Inc.), as previously described (*76*). All protocols used here with human cells were approved by the Duke University Health System Institutional Review Board. Cells were processed using EasySep Negative Selection Human Monocyte Enrichment kits (STEMCELL Technologies, catalog #19058), without depleting CD16, and then cultured in Dulbecco’s minimum essential medium (DMEM) (Invitrogen, catalog #11995073) supplemented with GlutaMAX (Thermo Fisher Scientific, catalog #35050061), 10% FBS (Atlanta Biologicals, catalog #S11150H), antibiotic-antimycotic (Thermo Fisher Scientific, catalog #15240062), and animal-free recombinant human macrophage CSF (M-CSF) (20 ng/ml, PeproTech, catalog #AF-300-25). The cells were cultured for 7 days before the experiments to obtain human macrophage characteristics and treated with α-syn PFFs. MDM cultures incubated with α-syn PFFs for periods of time indicated in figure legends were then washed thrice with endotoxin-free PBS from Sigma-Aldrich (catalog #TMS-012-A). After three wash cycles with endotoxin-free PBS on ice, MDM cells were fixed with 4% paraformaldehyde (PFA) (EMS, catalog #15700). PBS was supplemented with 5% bovine serum albumin (BSA) (Sigma-Aldrich, catalog #A4612), fluorescein isothiocyanate (FITC) pre-conjugated LAMP1 monoclonal antibody (LY1C6; 1:100, Thermo Fisher Scientific, catalog #MA1-164, RRID:AB_2536869), Galectin-3 monoclonal antibody (A3A12; Thermo Fisher Scientific, catalog #MA1-940, RRID:AB_2136775), and Hoechst 33342 (1:5000, BD Biosciences, catalog #561908, RRID:AB_2869394). A detailed protocol is available: dx.doi.org/10.17504/protocols.io.14egn7rzyv5d/v1.

### Primary neuron culture preparation and analysis

All procedures involving mice were approved by the Duke Institutional Animal Care and Use Committee (A205-21-10-42 and A206-21-10). Primary hippocampal neuronal cultures were prepared from postnatal (P0) pups of nTg (The Jackson Laboratory, JAX stock #000664, RRID:IMSR_JAX:000664), human-PAC-wt-*SNCA*^+/+^/*Snca*^−/−^ (JAX stock #010710, RRID:IMSR_JAX:010710), or *Snca*^−/−^ (JAX stock #016123, RRID:IMSR_JAX:016123) and cultured as previously described (*68*). Briefly, the hippocampi were isolated and dissected in Hibernate E medium (VWR, catalog #MSPP-HE). The tissue was then digested in a papain solution (Worthington, catalog #LS003126) in a Hanks’ balanced salt solution (HBSS) buffer (Thermo Fisher Scientific, catalog #14025126), supplemented with 10 mM Hepes (pH 7.4), 100 mM sodium pyruvate, and 1% penicillin/streptomycin (Thermo Fisher Scientific, catalog #10378016). The cells were plated and incubated in Neurobasal medium (Thermo Fisher Scientific, catalog #21103049) with B-27 (Thermo Fisher Scientific, catalog #17504044), 5 mM GlutaMAX, and 10% FBS (Atlanta Biologicals, catalog #S11150H) in wells coated with poly-d-lysine (0.1 mg/ml; Thermo Fisher Scientific, catalog #A3890401) at a density of 10,000 cells per cm^2^. After 12 hours, the plating medium was replaced with a maintenance medium that included a Neurobasal medium supplemented with B-27 and 0.5 mM l-glutamine. The primary cultures that reached DIV7 were used in experiments. A detailed protocol is available: dx.doi.org/10.17504/protocols.io.j8nlkk241l5r/v1.

Primary hippocampal neurons at DIV7 prepared according to the aforementioned protocol were treated with α-syn PFFs at a concentration of 0.64 nM or 64 pM (as indicated in figure legends as 10 μg/ml or 1 μg/ml) relative to estimated molecular weight measured as calculated size by DLS acquisitions and protein weight measured via NanoDrop. For ELISA measurements, primary neuron cultures in 48-well plates at DIV21 were lysed in 200 μl of a lysis buffer containing 1% Triton, protease, and phosphatase inhibitors in PBS. Cells were thoroughly scraped and resuspended before transferring into Eppendorf tubes. The collected samples were then sonicated using a water-bath sonicator (Qsonica700, Qsonica Inc.) at 30% amplitude at 10°C for a duration of 10 min. The sonicated cell suspensions were then centrifuged at 10,000*g* for 20 min at 4°C. The supernatants were aliquoted and stored at −80°C for subsequent bicinchoninic acid (BCA) analysis (Thermo Fisher Scientific, catalog #23225) and ELISA measurements. For immunofluorescence analysis, culture medium at DIV21 was removed and fixed with 2% PFA (EMS, catalog #15700) in TBS. Samples were then permeabilized in 3% normal donkey serum (Equitech-Bio, catalog #SD32-0500) supplemented with 0.1% saponin (Sigma-Aldrich, catalog #S4521-25G) in TBS for 30 min. Primary antibodies including anti–α-syn (phospho-S129) antibody EP1536Y (1:4000, Abcam, catalog #ab51253, RRID:AB_869973), Tau5 (1:2000, Thermo Fisher Scientific, catalog #AHB0042, RRID:AB_1502093), and NeuN (1:2000, GeneRwx, catalog #GTX00837, RRID:AB_2937041) were incubated in 0.02% saponin and 1% normal donkey serum in TBS overnight at 4°C with gentle agitation. After three washing rounds with TBS, secondary antibodies, including anti-rabbit Alexa Fluor 647 (Thermo Fisher Scientific, catalog #A-31573, RRID:AB_2536183), anti-mouse Alexa Fluor 488 (Thermo Fisher Scientific, catalog #A-21131, RRID:AB_2535771), anti-chicken Alexa Fluor 555 (Jackson ImmunoResearch, catalog #703545155, RRID:AB_2340375), and Hoechst 33342 dye (1:10,000), were incubated with the cells overnight at 4°C with orbital shaking. Images were obtained using Keyence BZ-X810 and Zeiss880 and coded for a blinded approach to analyze using CellProfiler Image Analysis Software (RRID:SCR_007358; Broad Institute) and ImageJ (RRID:SCR_003070; Research Services Branch National Institute of Mental Health). A detailed protocol is available: dx.doi.org/10.17504/protocols.io.81wgbxe13lpk/v1.

### iPSC-DA preparation and analysis

All protocols used here with human cells were approved by the Northwestern University Institutional Review Board. The iPSCs were differentiated into midbrain dopaminergic neurons following the previously published protocol (*123*, *124*). Neurons were cultured in Neurobasal SM1 medium (Thermo Fisher Scientific, catalog #21103-049) containing NeuroCult SM1 supplement (STEMCELL Technologies, catalog #05711), 1% penicillin/streptomycin (Thermo Fisher Scientific, catalog #10378016), and 1% l-glutamine (Gibco, catalog #25030081). Neurons were aged to 60 to 70 days for each experiment as indicated. Neurons were cultured on either 48-well (8 mm; Warner Instruments, catalog #64-0701) or 24-well (12 mm; VWR, catalog #48366-251) sized coverslips sequentially coated with poly-d-lysine (Sigma-Aldrich, catalog #P1149-10 mg) and laminin (Sigma-Aldrich, catalog #11243217001). Before undergoing differentiation, all iPSCs were maintained in mTeSR1 medium (STEMCELL Technologies, catalog #85850) on Cultrex (Thermo Fisher Scientific, catalog #343301001)–coated plates. For iPSC-DA neuron characterization, the 60- to 75-day-old cultures were permeabilized and blocked with a blocking solution composed of 0.1% Triton X-100 in PBS (Thermo Fisher Scientific, catalog #10010-049) supplemented with 2% BSA (Roche, catalog #03117057001) and 5% normal goat serum (Jackson ImmunoResearch, catalog #005-000-121) overnight at 4°C to prevent nonspecific antibody binding. Neurons were incubated with primary antibodies against βIII-tubulin (1:333, BioLegend, catalog #802001, RRID:AB_2564645), tyrosine hydroxylase (1:333, TH; EMD Millipore, catalog #AB9702, RRID:AB_570923), and FOXA2/HNF3β (1:333, Santa Cruz Biotechnology, catalog #sc-101060, RRID:AB_1124660) in blocking solution overnight at 4°C and washed three times with a 0.1% Triton X-100 PBS wash solution, followed by a 1-hour, room temperature incubation with secondary antibodies Alexa Fluor 568 donkey anti-rabbit immunoglobulin G (IgG) (Invitrogen, catalog #A10042, RRID:AB_2534017), Alexa Fluor 488 goat anti-chicken IgY (Invitrogen, catalog #A32931, RRID:AB_2762843), and Alexa Fluor 647 donkey anti-mouse IgG (Invitrogen, catalog #A-31571, RRID:AB_162542) all at 1:500. The cells were then washed three times with wash solution and mounted onto microscope slides (Fisher Scientific, catalog #12-550-15) with DAPI Fluoromount mounting medium (Southern Biotech, catalog #0100-20) and imaged. For seeding experiments, 60- to 75-day-old iPSC-DA neurons cultured on coverslips were treated with Alexa Fluor 568 mouse and human α-syn PFFs at a concentration of 10 μg/ml. During the 7 days of incubation, the medium was changed every 48 hours. Dopaminergic neurons were immunostained as described above with βIII-tubulin (1:500, Abcam, catalog #ab41489, RRID:AB_727049), anti–α-syn (phospho-S129) antibody EP1536Y (1:2000, Abcam, catalog #ab51253, RRID:AB_869973), and DAPI Fluoromount-G (Southern Biotech, catalog #0100-20).

### Endocytosis and internalization assays

For endocytosis assay in primary hippocampal culture curated from nTg (The Jackson Laboratory, JAX stock #000664, RRID:IMSR_JAX:000664), P0 pups at DIV7 were treated with endocytosis inhibitors including 50 μM ethyl-isopropyl amiloride (EIPA, Caymanchem, catalog #14406), 50 μM Dyngo 4a (Abcam, ab120689), 0.2 μM wortmannin (Sigma-Aldrich, catalog #W1628-1MG), 15 μM Pitstop 2 (Abcam, catalog #ab120687), or 2 mM methyl-β-cyclodextrin (Sigma-Aldrich, catalog #C4555-1G) or 0.001% DMSO (Millipore, catalog #D2650-5X5ML; vehicle control) for 30 min before incubation with pHrodo-conjugated α-syn PFFs. The internalization rate was measured by the proportion of fluorescence emitting from the conjugated fibrils and DAPI count with added cell mask. A detailed protocol is available: dx.doi.org/10.17504/protocols.io.4r3l22j5ql1y/v1.

Uptake of ~60-day-old iPSC-DA neurons were assessed by the treatment of Alexa Fluor 568 human and mouse α-syn PFFs at 10 μg/ml compared to untreated control cells to measure uptake. Following 8 hours of treatment, dopaminergic neurons were fixed in 4% PFA (Polysciences, catalog #40181) for 30 min and then washed with PBS (Thermo Fisher Scientific, catalog #10010-049) three times. The total cell membrane was labeled using CellTag 700 (Li-Cor Biosciences, catalog #926-41090) according to the manufacturer’s instructions. In brief, CellTag 700 was reconstituted in 100 μl of PBS, vortexed for ~45 s, and allowed to rehydrate in the dark for 30 min. The reconstituted CellTag 700, diluted to 1:500, was added to a coverslip within a 48 injected material-well plate, corresponding to a final volume of 300 μl. Following a 1-hour room temperature incubation protected from light, the cells were washed three times with PBS, and 300 μl of 0.1% trypan blue (Invitrogen, catalog #T10282) was added for 5 min to quench extracellular α-syn fibril signal. The wells were aspirated, and the coverslips were mounted onto microscope slides (Fisher Scientific, catalog #12-550-15) with DAPI Fluoromount mounting medium (Southern Biotech, catalog #0100-20) and imaged.

For internalization analysis in MDM cultures, cells were treated with Alexa Fluor 647 human and mouse α-syn PFFs at 1 μg/ml for the indicated time followed by subsequent wash cycles with PBS and fixation in 4% PFA in PBS and immunostained for LAMP1 and Hoechst 33342.

### Enzyme-linked immunosorbent assays

Human and mouse α-syn aggregate levels were measured according to the manufacturer’s protocol (BioLegend, catalog #449407). In brief, collected cell lysates were diluted (1:100) before adding to the antibody-coated wells along with two separate standard curves of serially diluted mouse and human α-syn PFFs. Absorbance was recorded at 450 nm using a SPECTROstar plate reader (BMG Inc.).

A detailed protocol is available: dx.doi.org/10.17504/protocols.io.5qpvob8odl4o/v1. Chemokines and cytokines were analyzed via R&D DuoSet ELISA systems (Human CCL5/RANTES DuoSet ELISA, catalog #DY278; DuoSet ELISA Ancillary Reagent Kit 2, catalog #DY008; Human IL-6 Elisa Kit, catalog #DY206-05; R&D Systems) according to the manufacturer’s instructions and included human IL-6 and CCL5.

### Stereotaxic intracranial injections and processing

Intracranial injections of α-syn fibrils, or vehicle control, were performed as previously described (*46*). Transgenic mice SNCA-OVX [B6.Cg-Tg(SNCA)OVX37Rwm Snca^tm1Rosl^/J] obtained from The Jackson Laboratory (JAX stock #023837; RRID:IMSR_JAX:023837 were randomized to groups spread across multiple cages. Stereotaxic injections of α-syn PFFs (10 μg per injection, equivalent protein weight for both human and mouse variants) were performed on mice at 4 to 5 months of age. A solution containing the indicated amount of α-syn PFFs (2 μl per each injection), or vehicle control, was injected into the right dorsal striatum relative to bregma: 1.0 mm anterior, 1.85 mm lateral, and 3.0 mm ventral relative to the skull. Three months after injections, mice were anesthetized with isoflurane and transcardially perfused with PBS followed by freshly prepared 4% PFA buffered in PBS. Removed brains were postfixed for 12 hours in a 4% PFA in PBS followed by an incubation in a 30% sucrose PBS for 48 hours. Frozen brains were then sectioned to 40 μm using a freezing microtome (Leica SM2010 R Sliding Microtome) and placed into six-well plates with 50% glycerol in PBS. Sections were incubated in an antigen retrieval buffer (10 mM sodium citrate, pH 6.0, supplemented with 0.05% Tween 20) for 30 min with gentle rocking at 37°C followed by three wash cycles in TBS (Thermo Fisher Scientific, catalog #J60764.K2). Rinsed sections were incubated in 5% donkey serum and 0.3% Triton X-100 in TBS for 1 hour at room temperature. For α-syn pathology detection, sections were incubated with anti–α-syn (phospho-S129) antibody EP1536Y (1:2500; Abcam, catalog #ab51253, RRID:AB_869973) primary antibodies in TBS buffer supplemented with 2.5% donkey serum and 0.1% Triton X-100 for 24 hours. Following wash cycles in TBS, sections were then incubated with donkey anti-rabbit Alexa Fluor 488 (1:500; Abcam, catalog #A32790, RRID:AB_2762833) secondary antibodies and Hoechst 33342 (1:5000, BD Biosciences, catalog #561908, RRID:AB_2869394) for 24 hours. Immunostained sections were mounted on Superfrost slides with ProLong Gold Antifade Mountant (Thermo Fisher Scientific, catalog #P36930) and stored at 4°C until imaging on the slide scanner VS200 Olympus. To quantitatively measure the distribution of pSyn-positive areas, an artificial intelligence (AI) model designed in QuPath (RRID:SCR_018257; Queens University Belfast) software was implemented and applied to each collected brain section image. Further sections were imported into QuickNII (RRID:SCR_016854; University of Oslo) software to align with the Allen Brain Atlas mouse brain edition (2017) followed by further adjustment using VisuAlign (RRID:SCR_017978; University of Oslo) software.

pSyn area calculations were performed using Nutil (RRID:SCR_017183; University of Oslo) software, and data were analyzed as the proportion of pSyn area to the brain region area for each section. The average of 15 to 25 collected sections per animal was calculated to generate a data point used to build a group analysis plot in GraphPad software. A detailed protocol is available: dx.doi.org/10.17504/protocols.io.4r3l22y6jl1y/v1. Analysis was performed by investigators blinded to sample identity until final data curation using coded identifiers for slides, mice, and injected material.

### α-Syn sedimentation

Seeding and fibrillization growth was assayed by adding 1% α-syn PFFs (3 μg/ml) into a fibrillization reaction that contained freshly thawed mouse or human α-syn monomer (0.3 mg/ml). The fibrillization reactions were performed according to the RT-QuIC approach amyloid dye-free environment in triplicate, with a volume of 50 μl for each specified time point. The resulting reactions were analyzed using the previously described slot-blot analysis or through SDS-PAGE with a Coomassie stain. A detailed protocol is available: dx.doi.org/10.17504/protocols.io.6qpvr3nwpvmk/v1.

### Statistical analyses

Statistical analyses were performed using the GraphPad Prism 9 software (RRID:SCR_002798; GraphPad Inc.). The specific statistical tests used for each dataset are detailed in the legends of each figure.

## Supplementary Materials

### The PDF file includes:

Tables S1 and S2 Figs. S1 to S13 Legend for data S1 References
