# Supplementary material for: Mouse α-synuclein fibrils are structurally and functionally distinct from human fibrils associated with Lewy body diseases
Source: Sci Adv. 2024 Nov 1;10(44):eadq3539. doi: 10.1126/sciadv.adq3539 (PMC11800946; doi:10.1126/sciadv.adq3539)
Supplement: Supplementary file 1 — Tables S1 and S2 Figs. S1 to S13 Legend for data S1 References [file sciadv.adq3539_sm.pdf]

Science Advances
AAAS

Supplementary Materials for

Mouse α-synuclein fibrils are structurally and functionally distinct from human fibrils associated with Lewy body diseases

Arpine Sokratian *et al.*

Corresponding author: Andrew B. West, andrew.west@duke.edu; Hilal A. Lashuel, hilal.lashuel@epfl.ch

Sci. Adv. **10**, eadq3539 (2024)
DOI: 10.1126/sciadv.adq3539

The PDF file includes:

Tables S1 and S2
Figs. S1 to S13
Legend for data S1
References

Other Supplementary Material for this manuscript includes the following:

Data S1

Supplemental Tables

table S1. Statistics of cryo-EM data collection and structure refinement of human α-syn fibril structure rederived at Duke University in this study (similar to previously described 6sst from EPFL)

Fibril type:	Human (similar to 6sst)
**Data collection**	
**Pixel size (Å)**	1.08
**Defocus Range (μm)**	-2.5 to -0.8
**Voltage (kV)**	300
**Exposure time (s/ movie)**	8
**Number of frames**	55
**Total dose (e^-/ Å2)**	55
**Micrographs**	6,530
**Inter-box distance (pixels)**	27
**Initially extracted fragments**	1,689,529
**Segments after 2D classification**	526,795
**Reconstruction**	
**Segments after 3D classification**	81,190
**Resolution after 3D refinement (Å)**	2.8
**Final resolution (Å)**	2.7
**Estimated map sharpening B-factor (Å^2)**	-68
**Helical rise (Å)**	4.82
**Helical twist (°)**	-0.75
**Atomic model**	
**EMDB code**	N/A
**PDB code**	N/A
**FSC threshold**	0.143
**Composition**<br>- Chains<br>- Atoms(non-Hydrogen)<br>- Residues	N.D.
**B-factors (Å2)**<br>-Protein	
**R.M.S. deviations**<br>- Bond lengths (Å)<br>- Bond angles (°)	
**Validation**<br>- MolProbity score<br>- Clashscore<br>- Poor rotamers (%)	
**Ramachandran plot**	
-Favored (%)	
-Allowed (%)	
-Disallowed (%)	

N.D. is not determined, N/A is not available, R.M.S. is root mean square

table S2. Structural comparison of mouse (Duke University) α-syn fibrils to other α-syn fibrils

PDB code	Recombinant protein	Buffer and additives*	RMSD(Å) from 8uie** Global / Only residues 54-66 / Rung core alignment	Structural Geometry Crossover distance (Å) / Helical twist (°) / Helical rise (Å)	Reference^{++}
**Recombinant α-syn fibril structures**					
8uie	Mouse (Duke)	PBS, pH 7.6	-	1035 / -0.84 / 4.84	this study
9ewv	Mouse (EPFL)	PBS, pH ~7.2 to 7.4	1.312 / 1.070 / 1.690	1050 / -0.8 / 4.8	this study
6ufr	E46K mutant human α-syn	15 mM $C_{16}H_{36}BrP$	2.909 / 1.115 / 3.107	800 / -1.08 / 4.85	(*25*)
6sst	Human α-syn polymorph 2B	50 mM Tris-HCl, 150 mM KCl, pH 7.5	4.322 / 1.739 / 4.611	1183 / -0.73 / 4.8	(*21*)
6rtb	Human α-syn polymorph 2B		3.149 / - / -	1210 / -0.73 / 4.91	
6ssx	Human α-syn/N-terminal acetylated/pS129 polymorph 2A		4.341 / 1.702 / 4.592	1080 / -0.8 / 4.8	
6rt0	Human α-syn/N-terminal acetylated/pS129polymorph 2A		4.269 / 1.746 / 4.657	1107 / -0.8 / 4.92	
7v4d	N-terminal acetylated human α-syn	50 mM Tris-HCl, 150 mM KCl, pH 7.5, 0.05% NaN_3, 600 µg per mL of heparin	10.795 / - / -	641 / -1.35 / 4.81	(*125*)
8hzb			12.098 / - / -	1033 / -0.84 / 4.82	
7v4c			9.587 / 1.378 / 9.851	282 / -3.06 / 4.8	
6h6b	Truncated (1-121) human α-syn	DPBS, pH 7.0–7.3	11.491 / - / -	882 / -1 / 4.9	(*18*)
6l1u	pY39-modified human α-syn	50 mM Tris, pH 7.5, 150 mM KCl, 0.05% NaN_3	10.244 / 1.096 / 22.332	1252 / -0.69 / 4.8	(*17*)
7uak	A53E mutant human α-syn	15 mM $C_{16}H_{36}BrP$	11.257 / - / -	877 / -0.99 / 4.74	(*126*)
7wnz	A53T mutant human α-syn	N/A	11.215 / - / -	613 / -1.39 / 4.74	N/A
8ce7	Human and/or JOS mutant α-syn	PBS	10.909 / - / -	542 / -1.58 / 4.76	(*5*)

8ceb			10.943 / - / -	973 / -0.88 / 4.76	
6cu8	Human α-syn	15 mM $C_{16}H_{36}BrP$	9.889 / - / -	460 / -1.88 / 4.8	(*7*)
6peo	H50Q mutant human α-syn	15 mM $C_{16}H_{36}BrP$	11.423 / - / -	893 / -0.97 / 4.81	(*127*)
7e0f	G51D mutant human α-syn	50 mM phosphate buffer, pH 7.0, 50 mM NaCl, 0.05% NaN_3	11.518 / 1.292 / 13.484	694 / -1.26 / 4.86	(*128*)
7lc9	Truncated (41-140) human α-syn	20 mM NaPi, pH 7.4, 140 mM NaCl	16.359 / - / -	527 / -1.64 / 4.80	(*129*)
7ynm	N-terminal acetylated α-syn and FLAG-tagged human α-syn	50 mM Tris-HCl, pH 7.5, 150 mM KCl, 50 μM ThT, 0.05% NaN_3	11.710 / - / -	537 / -1.62 /4.83	(*130*)
8adu	Human α-syn	50 mM HEPES, pH 7.4, 100 mM NaCl, lipid additives	15.619 / 1.132 / 23.134	889 / -0.95 / 4.69	(*131*)
8ads	Human α-syn		3.363 / 0.892 / 4.232	1029 / -0.82 / 4.69	
**Recombinant α-syn fibrils amplified from patient material**					
7v47	Human α-syn	20% PD CSF, 100 mM PIPES, pH 6.5, 500 mM NaCl,	2.816 / 1.314 / 3.217	784 / -1.12 / 4.88	(*20*)
7v48			2.829 / 1.462 / 3.214	803 / -1.08 / 4.82	
7v49			11.643 / - / -	703 / -1.24 / 4.84	
8h05			11.617 / - / -	703 / -1.24 / 4.84	(*132*)
8h03			2.902 / 1.360 / 3.275	798 / -1.1 / 4.88	
8h04			2.881 / 1.408 / 3.235	803 / -1.08 / 4.82	
7xo0			3.070 / 1.464 / 3.528	660 / -1.32 / 4.84	
7xo1			3.069 / 1.468 / 3.562	748 / -1.16 / 4.82	
7xo2			3.075 / 1.428 / 3.459	660 / -1.32 / 4.84	

7xo3			2.973 / 1.478 / 3.331	761 / -1.14 / 4.82	
7ozg	N-terminal acetylated α-syn	5% PMCA PD product, 50 mM HEPES, pH 7.4, 100 mM NaCl, 0.02% NaN 3	4.221 / 1.730 / 4.583	1080 / -0.78 / 4.68	(*24*)
7ozh	N-terminal acetylated α-syn	5% PMCA MSA product, 50 mM HEPES, pH 7.4, 100 mM NaCl, 0.02% NaN 3	3.784 / 1.636 / 4.215	1255 / -0.68 / 4.74	
7nca	Human α-syn	2 uM MSA brain homogenate (sarkosyl extracted), 100 mm PIPES, pH 6.5, 500 mm NaCl, 0.05% NaN_3	2.899 / 0.930 / 3.049	824 / -1.04 / 4.76	(*23*)
7ncg			2.808 / 0.836 / 3.000	900 / -0.95 / 4.75	
7nch			4.559 / 1.621 / 4.857	1000 / -0.86 / 4.78	
7nck			11.296 / - / -	900 / -0.95 / 4.75	
7nci			4.348 / 1.736 / 4.693	1110 / -0.77 / 4.75	
8cyx	Human α-syn	10% DLB CSF, PBS, pH 7.6	4.360 / 1.659 / 4.619	1152 / -0.75 / 4.8	(*133*)
8fpt	Human α-syn	10% DLB brain homogenate, 20 mM Tris-HCl, pH 8.0, 100 mM NaCl, 2% Triton X-100	16.05 / - / -	ssNMR	(*22*)
***Ex vivo* α-syn fibrils extracted from brain homogenates**					
8a9l	-	DLB/PD/PDD brain homogenate, 2% sarkosyl extraction, 20 mM Tris-HCl, pH 7.4 recovery	11.119 / 0.758 / 11.645	996 / 0.86 / 4.76	(*3*)
8bqv	-	JOS-α-syn brain homogenate, 2% sarkosyl extraction, 20 mM Tris-HCl, pH 7.4 recovery	11.995 / - / -	646 / 1.33 / 4.77	(*5*)
8bqw			11.987 / - / -	764 / -1.11 / 4.71	
6xyo	-	MSA brain homogenate, 2% sarkosyl extraction, 30 mM Tris-HCl, pH 7.4 recovery	11.587 / - / -	590 / -1.44 / 4.72	(*3*)
6xyp			10.465 / - / -	602 / -1.41 / 4.72	
6xyq			10.427 / - / -	634 / -1.34 / 4.72	

*DPBS is Dulbecco's phosphate-buffered saline, HEPES is (4-(2-hydroxyethyl)-1-piperazineethanesulfonic acid, NaPi is sodium phosphate buffer, PMCA PD product is α-syn amplified products from Parkinson's disease brain homogenates using protein misfolding

cyclic amplification approach, PD or DLB CSF is Parkinson's disease or dementia with Lewy bodies cerebrospinal fluid, MSA or DLB/PD/PDD brain homogenates is brain tissues from multiple system atrophy or Lewy disorders including dementia with Lewy bodies, Parkinson's disease, Parkinson's disease with dementia, used to extract α-syn species in indicated buffer conditions, JOS-α-syn is α-syn detected in juvenile-onset synucleinopathy disorders, PIPES is piperazine-1,4-bis(2-ethanesulfonic acid)

** Global is a global alignment using available (full) fibril structure, or, Only residue 54-66 indicates a regional fit and RMSD calculation only with the spanning region from 54-66, orm a Rung core alignment is full fibril structure RMSD calculation based on local alignment to the structurally conserved region 54-66.

Supplemental figures and legends

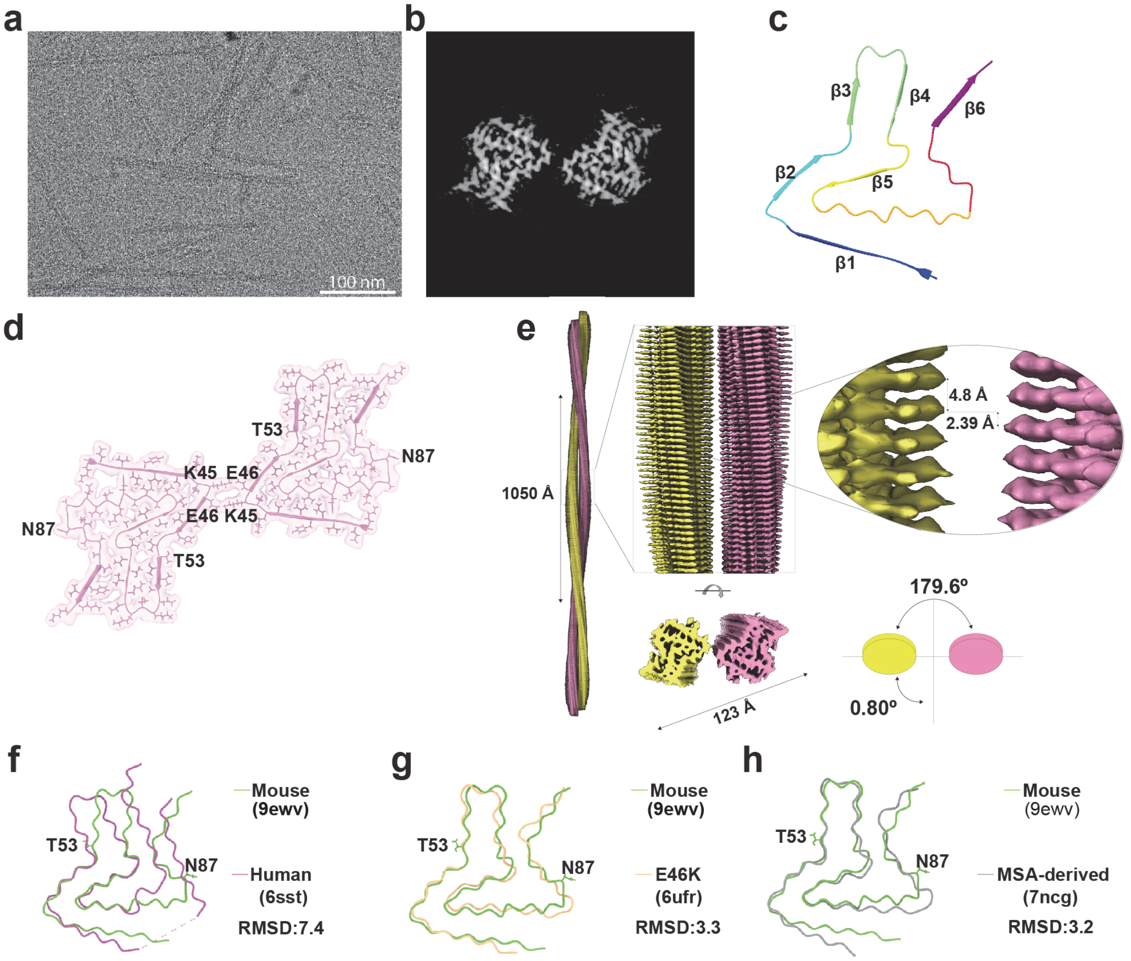

fig. S1. Cryo-EM structure and atomic models of EPFL-derived mouse α-syn fibrils.
(**a**) Representative cryo-EM micrograph of mouse α-syn fibrils with an indicated scale bar of 100 nm. (**b**) Cross-sectional view of the cryo-EM map density with detailed internal structure of a mouse α-syn fibril. (**c**) Cartoon representation of the fitted atomic model with the indicated six β-strands (β1-β6), each delineated by a distinct color. (**d**) Cryo-EM density map with the fitted atomic model of mouse α-syn spanning residues 34-97. Residues N87 and T53 are highlighted, noting their variation from the human α-syn sequence, as well as key bridge residues K45 and E45. (**e**) Representation of overall EPFL-derived mouse α-syn fibril architecture, noting a mean crossover distance of 1050 Å with a width of 123 Å, calculated pseudo-2 fold symmetry with a rise (per subunit indicated) of 2.39 Å and a twist of 179.6° (0.80°) highlighted. (**f**) Global fit comparative analysis of the mouse α-syn fibril (9ewv, green) structure and recombinant human α-syn fibril atomic model (6sst, magenta), or (**g**) recombinant E46K (6ufr, yellow), or (**h**) MSA-amplified (7ncg, gray), with visualized T53 and N87 amino acid schematics, and indicated aligned total RMSD values.

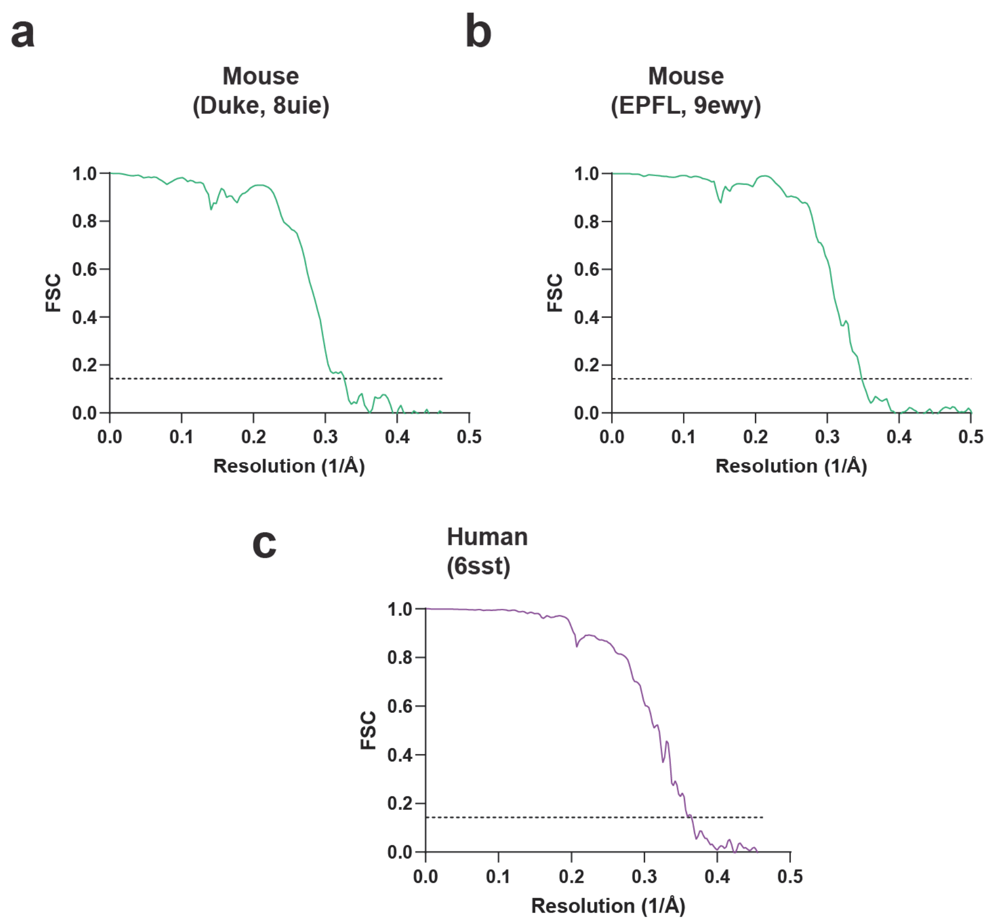

fig. S2. Resolution of the cryo-EM maps of mouse and human recombinant α-syn fibrils. Fourier shell correlation (FSC) plots between half-maps for the 3D reconstructions obtained from cryo-EM collected images of procured mouse fibrils generated by (**a**) Duke (**b**) and EPFL. (**c**) FSC plot of rederived human α-syn fibrils collected and generated at Duke University.

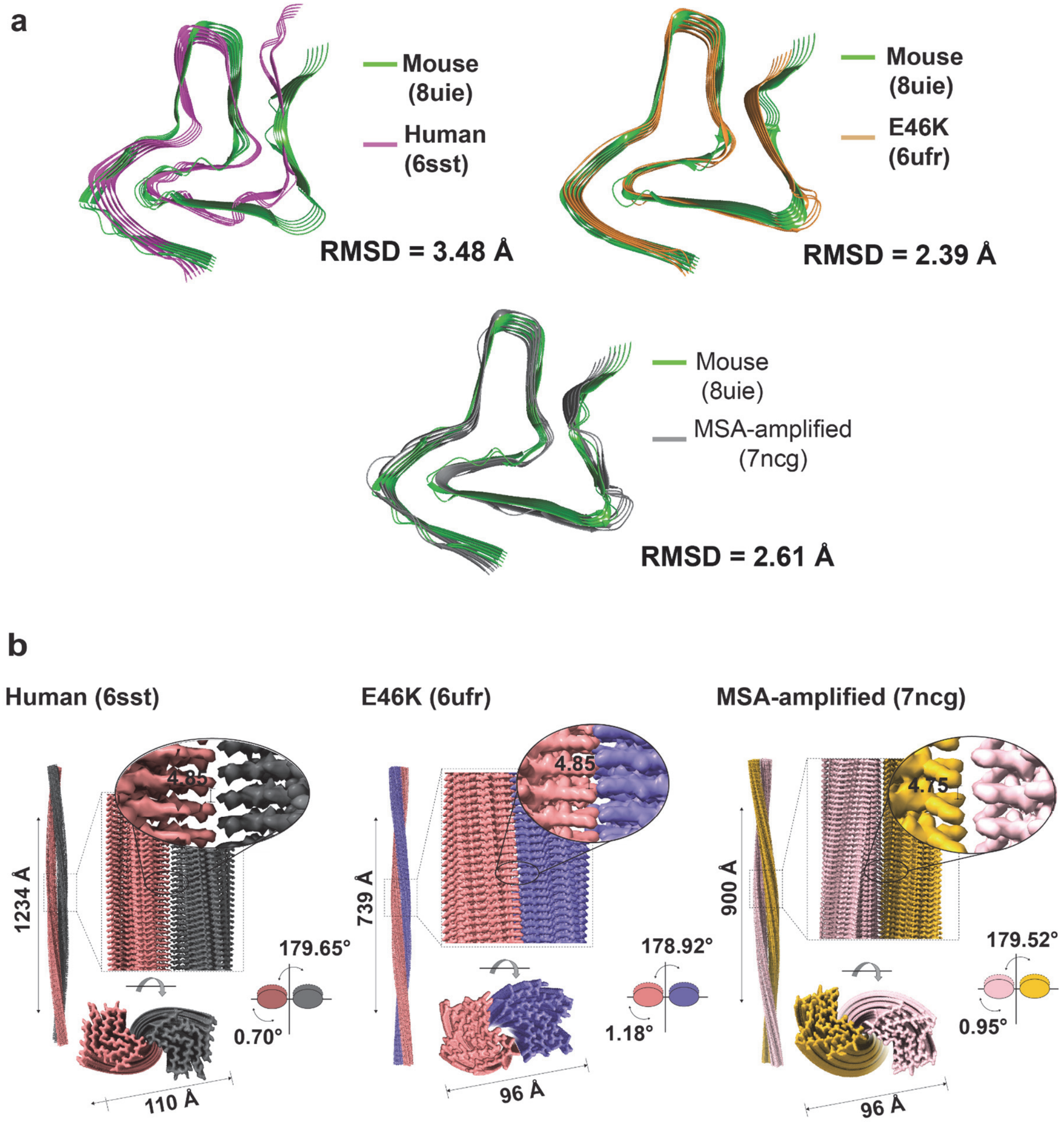

fig. S3. Mouse β-sheets closely align with E46K and MSA-amplified human α-syn fibril structures in global fit alignments of five stacked fibril rungs.

(**a**) Overlay of human, E46K, and MSA-amplified protofilament structures compared with the mouse α-syn protofilament structure generated at Duke (8uie). RMSD values represent the composite scores for the aligned protofilament structures from 5-high stacked fibril rungs. The RMSD values shown here, which are slightly different from that of alignments of individual rungs (see table S2), are with respect to a global fit comparison. (**b**) Cryo-EM 3D reconstruction density map of the human (6sst), E46K (6ufr), and MSA-amplified (7ncg) α-syn fibrils (i.e., two intertwined protofilaments), with a cross-section view. The pitch length, helical rise, and corresponding twist angle of the displayed fibrils are indicated.

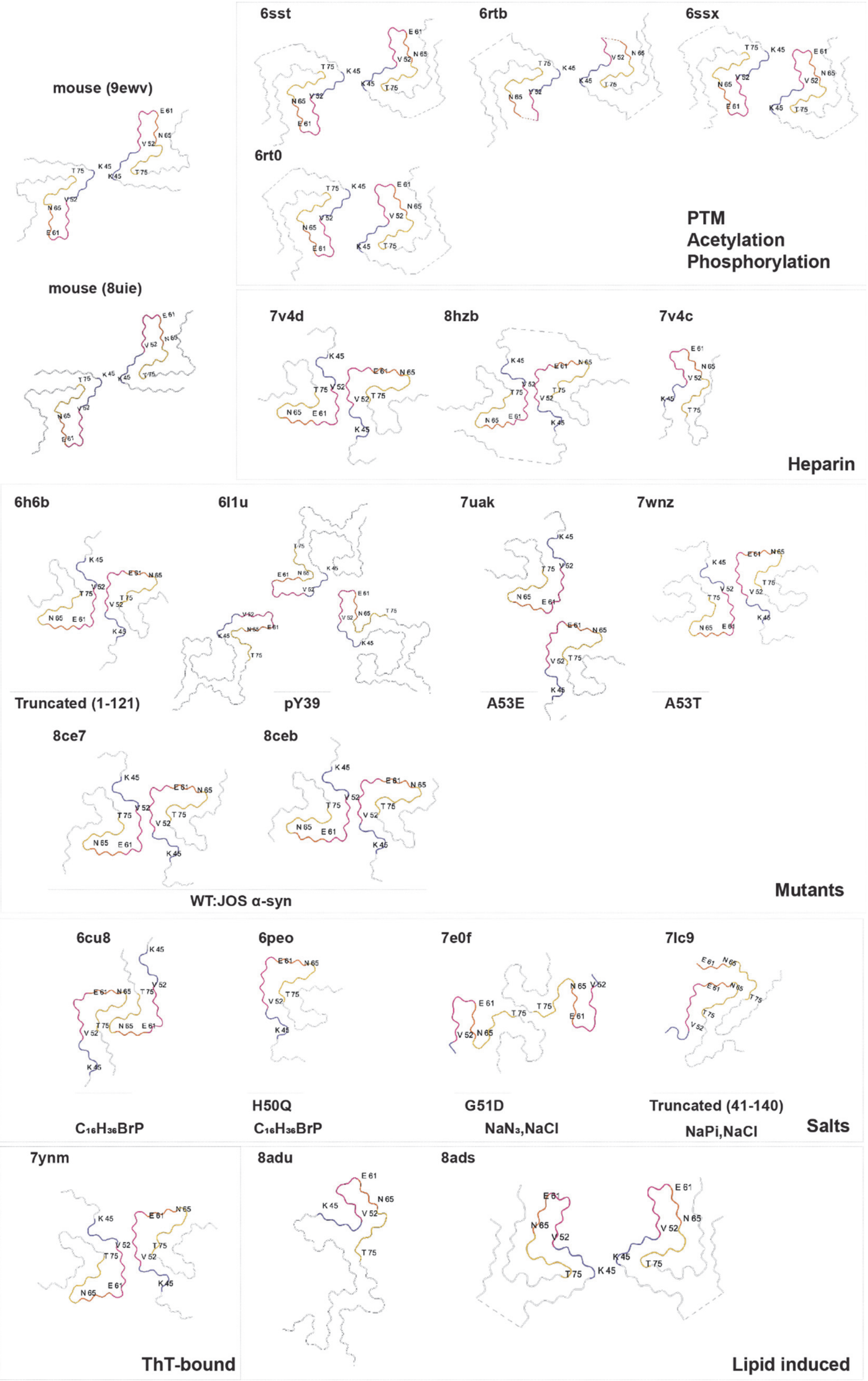

fig. S4. Summary of previously resolved recombinant α-syn fibril structures in relation to mouse α-syn fibril Cryo-EM maps.

Cryo-EM maps of mouse α-syn fibrils generated from EPFL (9ewv) and Duke (8uie) research groups with β-sheets color-coded: β2 (blue), β3 (red), β4 (orange), and β5 (yellow). Previously recorded structures are categorized based on the unique characteristics that were employed for fibril

generation. These characteristics are elaborated in table S2 along with alignment values. The color scheme is consistently maintained across the entire structure panel, serving to emphasize the structural features that are specific to the mouse α-syn structures.

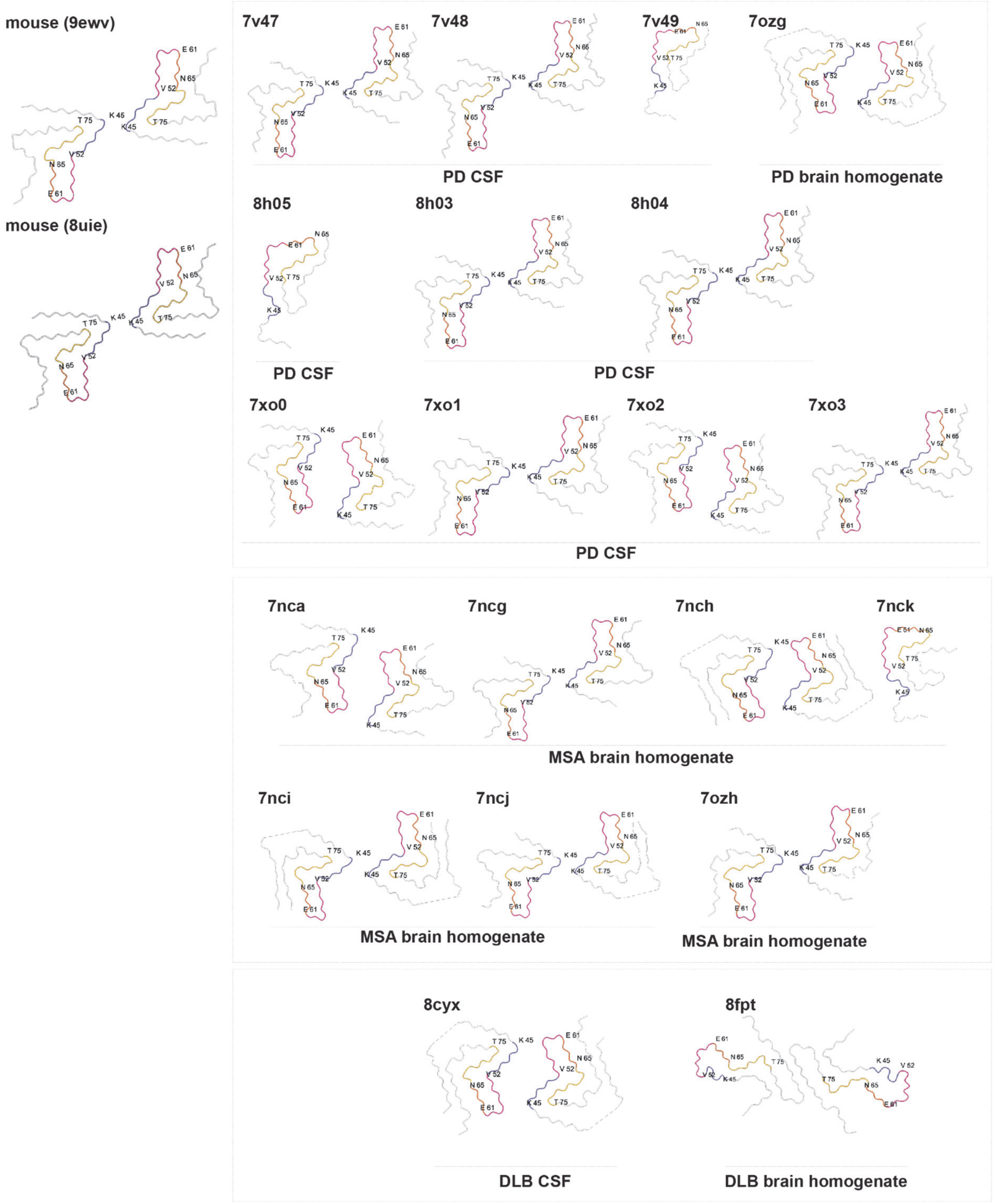

fig. S5. Structures of recombinant α-syn fibrils amplified from patient material in comparison to mouse α-syn fibril Cryo-EM maps

Cryo-EM maps of mouse α-syn fibrils curated at Duke (8uie) and EPFL (9ewv) with β-sheets color-coded as β2 (blue), β3 (red), β4 (orange), and β5 (yellow). Previously reported structures include α-syn fibrils generated in presence of patient material including brain homogenates and cerebrospinal fluid. Alignment values are detailed in table S2.

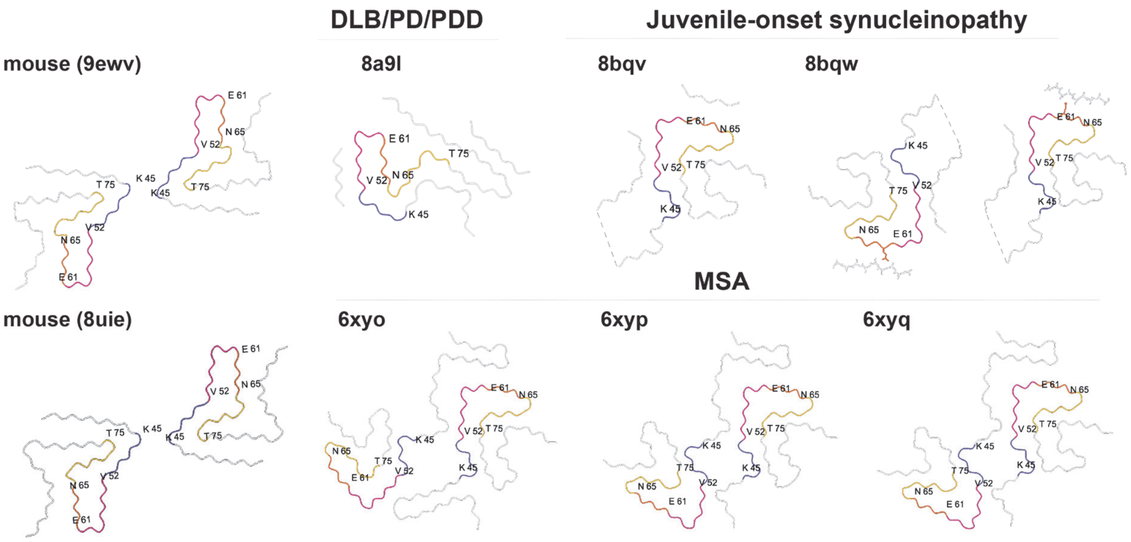

fig. S6. Structures of *ex vivo* α-syn fibrils sarkosyl extracted from brain homogenates in comparison to mouse α-syn fibril Cryo-EM maps.

Cryo-EM maps of mouse α-syn fibrils curated at Duke (8uie) and EPFL (9ewv) with β-sheets color-coded as β2 (blue), β3 (red), β4 (orange), and β5 (yellow). Previously reported structures include ex vivo α-syn fibrillar assemblies extracted from patient material and detailed in table S2. Note, the mouse α-syn fibrils structurally curated here appear unstable in sarkosyl extraction buffers (see fig S9), unlike the brain-derived extracted fibrils above.

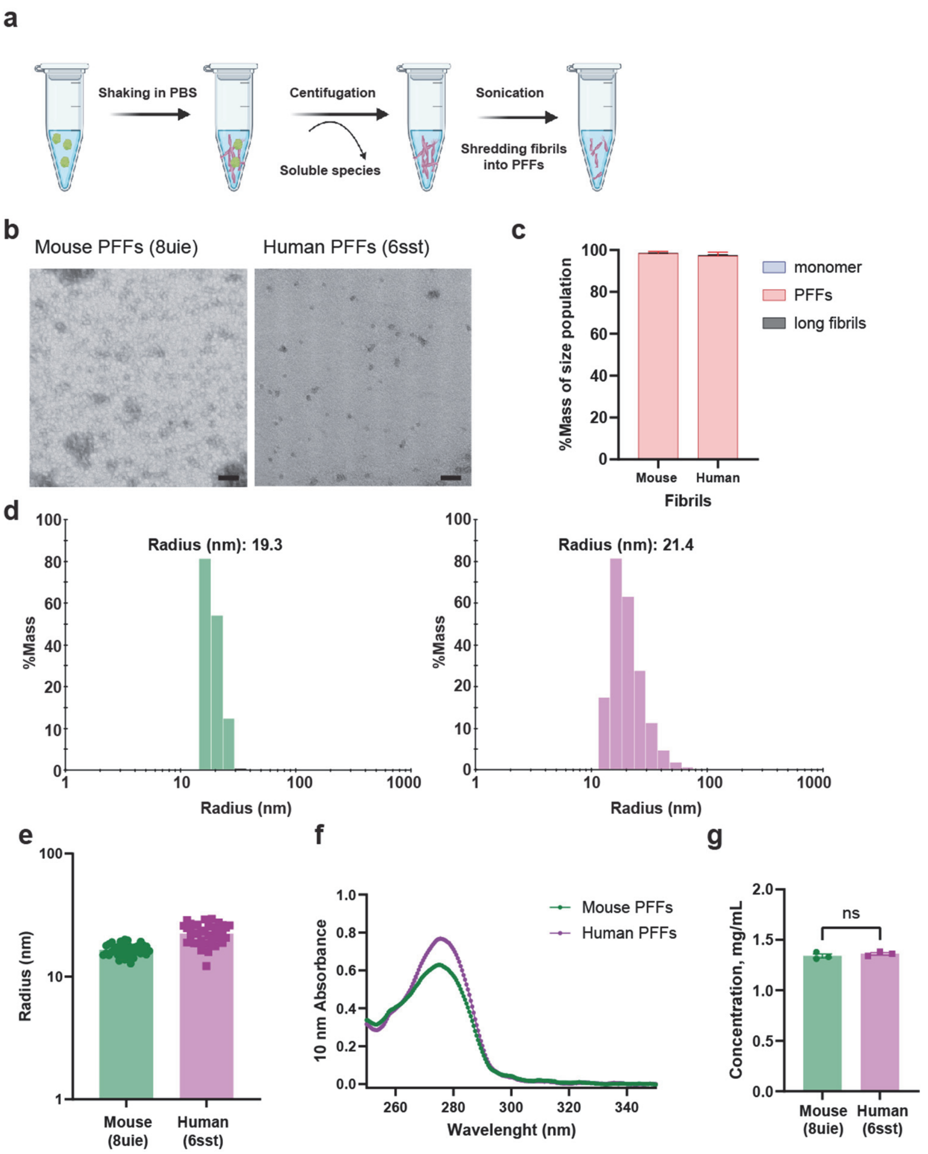

fig. S7. Generation and validation of mouse and human α-syn PFFs.

(**a**) Schematic representation of the α-syn PFF preparation process, which includes the initial formation of aggregates through continuous shaking in PBS, separation of large aggregates from oligomers and monomeric protein using multiple cycles of high-speed centrifugation, and sonication of heavy pellets to achieve a uniform size of very short fibrils, or PFFs. (**b**) Representative TEM of mouse and human PFFs after this process. (**c**) Group analysis of the size population proportions of sonicated mouse and human α-syn preparations corresponding to (**d** and **e**) typical radii distribution relative to percent of mass in the preparations. (**f**) UV absorbance spectra and (**g**) coefficient extinction adjusted protein concentration verification of PFF preparations. Each data point in panels c,e, and g show the mean values of acquisitions from three independent experiments. Each dot in the curves in panel f is the mean of two technical replicates from three independent batches of PFFs. Significance was assessed via 2-tailed t-tests and ns is not significant.

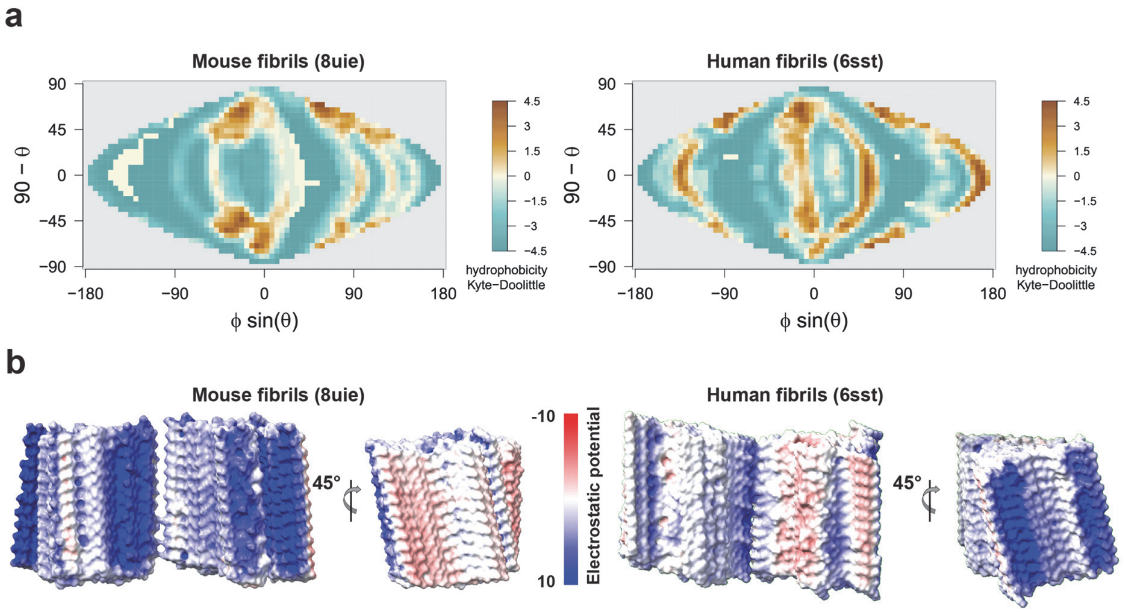

fig. S8. Mouse α-syn fibrils exhibit distinct characteristics of hydrophobicity and electrostatic surface charge.

(**a**) Molecular 2D Kyle-Doolittle hydrophobicity maps projecting 24 α-syn fibril layers of β-stacks with spherical coordinates (φ, θ) and the Sanson-Flamsteed 2D projection. The projected 2D map is divided into a grid of 36 × 72 cells. Each cell is smoothed by averaging its value with those of the eight surrounding cells, and is associated with the average of the corresponding hydrophobicity values. (**b**) Cross and side views of the electrostatic potential maps of mouse and human α-syn fibrils, generated using molecular dynamic (MD) simulations of atomic models placed under physiological pH (7.4) and salt conditions.

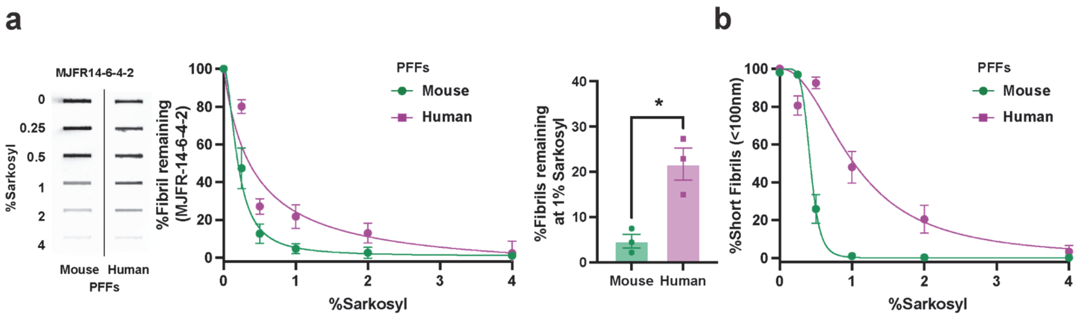

fig. S9. Mouse α-syn PFFs are highly susceptible to sarkosyl denaturation.
(a) Filter-trap slot-blot analysis of sonicated fibrils (PFFs) exposed to different concentrations of sarkosyl (left) and the remaining fibrils detected with the fibril-selective antibody MJFR14-6-4-2 in a dose-response curve (middle), with group analysis between two PFF variants at 1% Sarkosyl compared (right). Error bars indicate S.E.M from three independent experiments. (**b**) DLS analysis of sonicated fibrils (PFFs) exposed to sarkosyl concentrations in a dose-response curve. Error bars indicate S.E.M of three independent experiments with 10 acquisition measurements for each biological sample. *p<0.05 from unpaired 2-tailed t-tests.

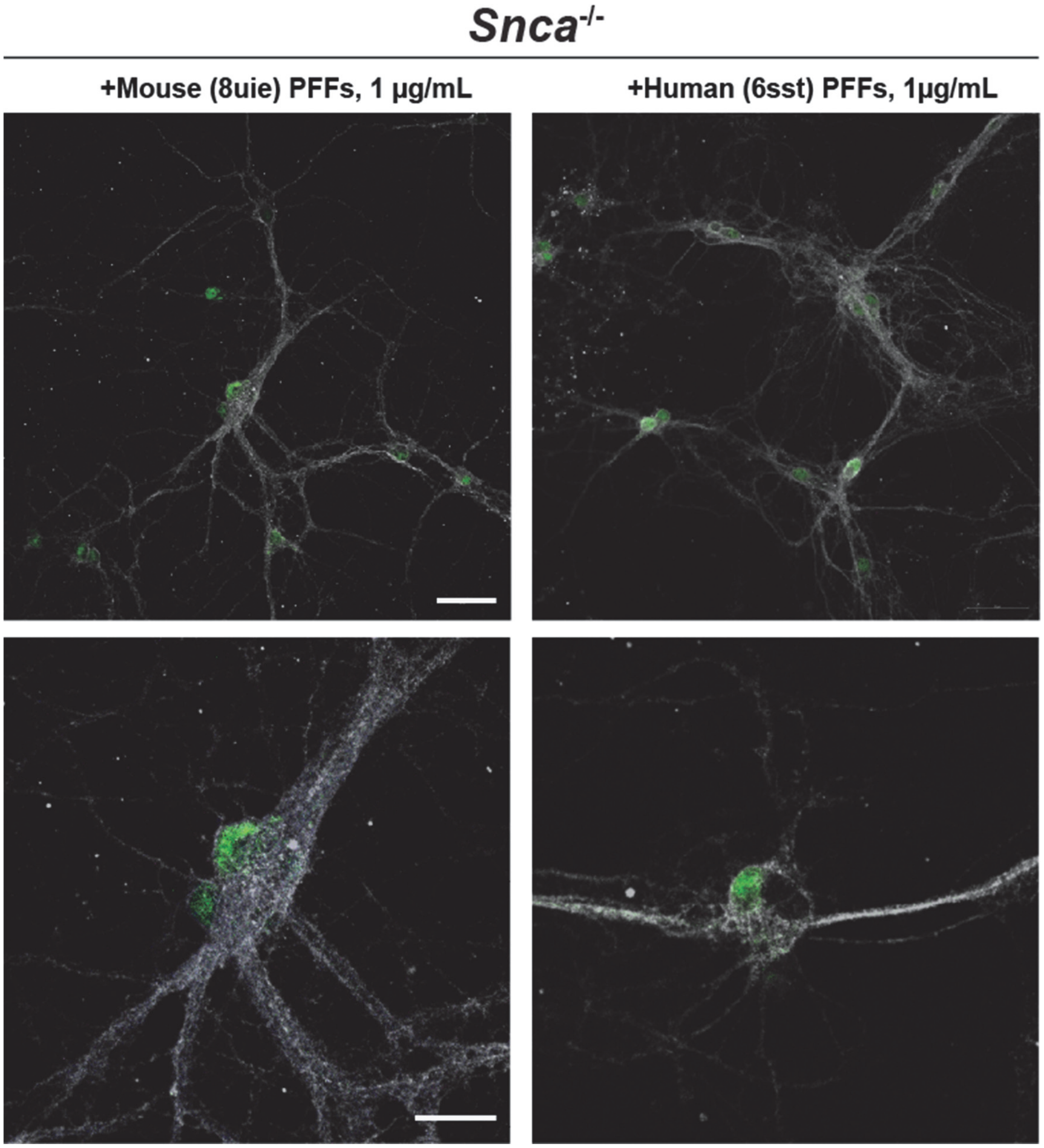

fig. S10. Seeding propensity following α-syn PFF treatment in *Snca*$^{-/-}$ hippocampal neuronal culture.

Shown is representative Immunostaining of mouse *Snca*$^{-/-}$ primary hippocampal neurons treated with mouse or human α-syn PFFs for 14 days before fixing. Grey color indicates tau, and green as NeuN, whereas sparse magenta staining indicates background signal from pS129-α-syn staining. The scale bar represents 50 µm for the main images and 20 µm for the magnified images. Negligible background staining is observable with the pS129-α-syn antibody in the α-syn null animals, indicating a good specificity for the staining approach. Notably, there were no detectable traces of the PFFs previously added, indicating the PFFs are unlikely to be phosphorylated and persist over time without the presence of endogenous α-syn.

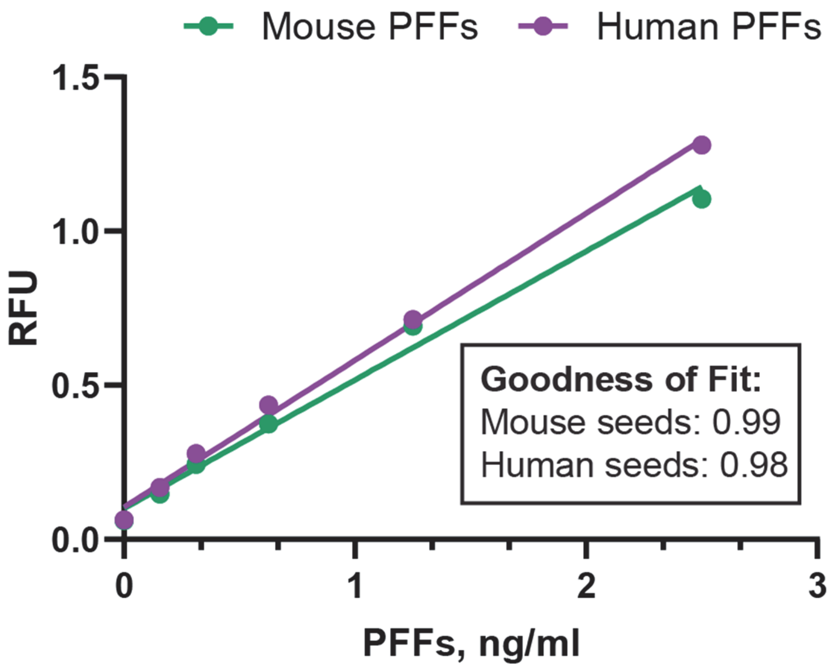

fig. S11. Evaluation of α-syn aggregation ELISA using control recombinant mouse and human α-syn fibril PFFs.

Standard curves generated from mouse and human PFFs in a pan-α-syn aggregate-specific ELISA. The ELISA approach was utilized to quantify the level of aggregates present in lysates derived from the neuronal cultures. The standard curve, along with the indicated goodness of fit and corresponding r^2 values, provides reliable measures at physiological ranges. Each data point represents the mean from three technical replicates from two independent experiments with errors bars indicating S.E.M.

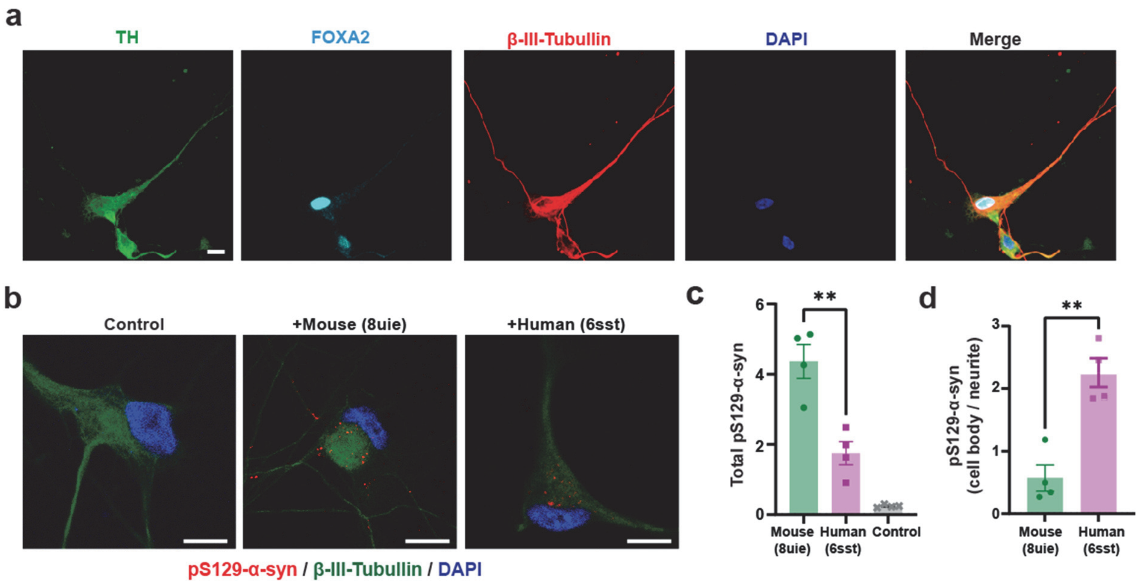

fig. S12. Elevated p-S129-α-syn levels in iPSC-derived dopaminergic neurons following treatment with mouse α-syn PFFs

(**a**) Representative Immunofluorescence images of ~70-day-old iPSCs-derived DA neurons stained with the neuronal marker β-III-tubulin (red), dopaminergic markers tyrosine hydroxylase (TH, green) and FOXA2 (cyan) counterstained with DAPI (blue). The scale bar is 10 µm. (b) Representative immunostaining of iPSCs-derived DA neurons treated with either Alexa-568 labeled mouse or human α-syn PFFs at 10 µg per mL for 7 days, along with intact control cells. iPSCs-derived DA neurons were fixed 7-days post initial treatment, permeabilized, and stained for β-III-tubulin (green) to outline neuronal architecture, pS129-α-syn (red), and DAPI (blue). The scale bar is 5 µm. (**b**) Levels of pS129-α-syn in the β-III-tubulin area in iPSC-derived DA neurons after 7 days of treatment with 10 µg/mL of PFFs or control. (**c**) The quantity of pS129-α-syn puncta relative to the β-III-tubulin area measured in each image and compared across conditions. (**d**) Proportion of abundance of pS129-α-syn puncta localized in cell bodies and neurites in group analysis between mouse and human α-syn PFF treatments. Each data point in c and d represent the mean value of the images from one well (n=4) and errors bars indicate S.E.M with **p<0.01 from 2-tailed t-tests.

a

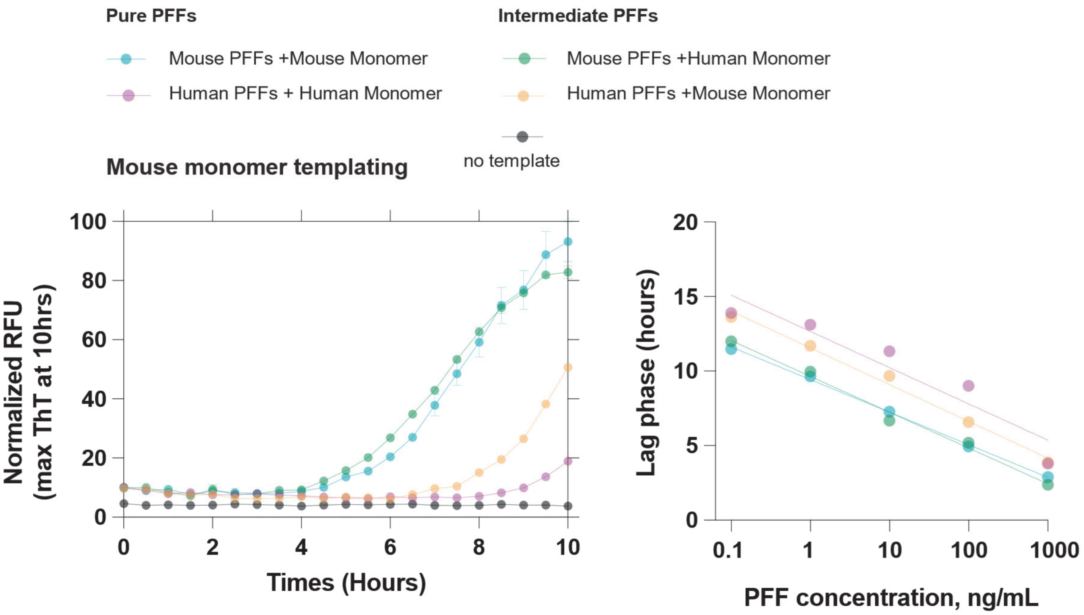

b

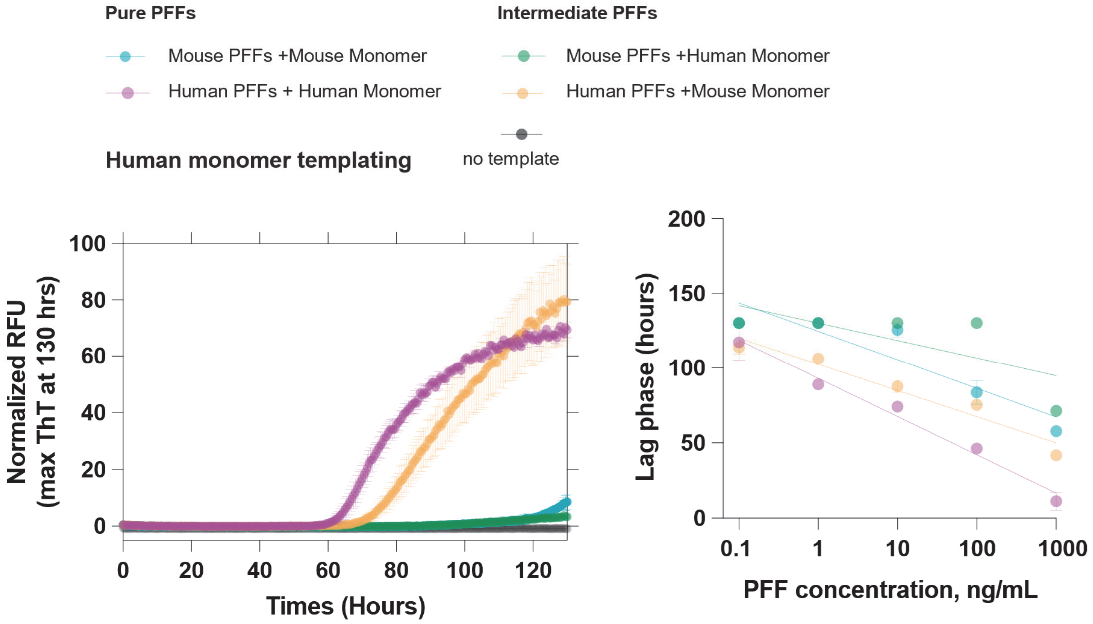

fig. S13. Evaluation of kinetic properties in aggregation assays with cross-seeded chimeric fibrils show that the chimeric fibrils largely replicate the functional properties of their parental seeds, despite different amino acid sequences between the fibril preparations.

(**a**) Representative RT-QuIC (real-time quaking induced) assays of normalized relative fluorescence values (RFUs) with mouse monomer templating on different PFF seeds in the creation of new fibrils. The lack of formation of spontaneous fibrils (gray color, no PFFs added "no template") indicate that spontaneous aggregation is not occurring under the given aggregation conditions within the specified timeframes. Line graphs show corresponding lag in amplification (in hours, or time to initial fluorescence threshold) of the different reactions combined with different PFF concentrations added and analyzed to a linear regression model. Data points represent normalized ThT fluorescence to their maximal fluorescence in the individual reactions, accounting for differential ThT binding to

different fibril structures. (**b**) Comparable reactions as above but with human instead of mouse monomer templating with the indicated PFF seed.

Data S1 contains all raw data associated with this manuscript and is available as supplementary materials online.

REFERENCES AND NOTES

1. M. S. Pollanen, D. W. Dickson, C. Bergeron, Pathology and biology of the Lewy body. *J. Neuropathol. Exp. Neurol.* **52**, 183–191 (1993).

2. M. G. Spillantini, M. L. Schmidt, V. M. Lee, J. Q. Trojanowski, R. Jakes, M. Goedert, α-Synuclein in Lewy bodies. *Nature* **388**, 839–840 (1997).

3. M. Schweighauser, Y. Shi, A. Tarutani, F. Kametani, A. G. Murzin, B. Ghetti, T. Matsubara, T. Tomita, T. Ando, K. Hasegawa, S. Murayama, M. Yoshida, M. Hasegawa, S. H. W. Scheres, M. Goedert, Structures of α-synuclein filaments from multiple system atrophy. *Nature* **585**, 464–469 (2020).

4. Y. Yang, Y. Shi, M. Schweighauser, X. Zhang, A. Kotecha, A. G. Murzin, H. J. Garringer, P. W. Cullinane, Y. Saito, T. Foroud, T. T. Warner, K. Hasegawa, R. Vidal, S. Murayama, T. Revesz, B. Ghetti, M. Hasegawa, T. Lashley, S. H. W. Scheres, M. Goedert, Structures of α-synuclein filaments from human brains with Lewy pathology. *Nature* **610**, 791–795 (2022).

5. Y. Yang, H. J. Garringer, Y. Shi, S. Lövestam, S. Peak-Chew, X. Zhang, A. Kotecha, M. Bacioglu, A. Koto, M. Takao, M. G. Spillantini, B. Ghetti, R. Vidal, A. G. Murzin, S. H. W. Scheres, M. Goedert, New SNCA mutation and structures of α-synuclein filaments from juvenile-onset synucleinopathy. *Acta Neuropathol.* **145**, 561–572 (2023).

6. L. Bousset, L. Pieri, G. Ruiz-Arlandis, J. Gath, P. H. Jensen, B. Habenstein, K. Madiona, V. Olieric, A. Böckmann, B. H. Meier, R. Melki, Structural and functional characterization of two alpha-synuclein strains. *Nat. Commun.* **4**, 2575 (2013).

7. B. Li, P. Ge, K. A. Murray, P. Sheth, M. Zhang, G. Nair, M. R. Sawaya, W. S. Shin, D. R. Boyer, S. Ye, D. S. Eisenberg, Z. H. Zhou, L. Jiang, Cryo-EM of full-length α-synuclein reveals fibril polymorphs with a common structural kernel. *Nat. Commun.* **9**, 3609 (2018).

8. L. Frey, D. Ghosh, B. M. Qureshi, D. Rhyner, R. Guerrero-Ferreira, A. Pokharna, W. Kwiatkowski, T. Serdiuk, P. Picotti, R. Riek, J. Greenwald, On the pH-dependence of α-synuclein amyloid polymorphism and the role of secondary nucleation in seed-based amyloid propagation. *eLife* **12**, RP93562 (2023).

9. S. Zhang, R. Zhu, B. Pan, H. Xu, M. F. Olufemi, R. J. Gathagan, Y. Li, L. Zhang, J. Zhang, W. Xiang, E. M. Kagan, X. Cao, C. Yuan, S.-J. Kim, C. K. Williams, S. Magaki, H. V. Vinters, H. A. Lashuel, B. A. Garcia, E. James Petersson, J. Q. Trojanowski, V. M.-Y. Lee, C. Peng, Post-translational modifications of soluble α-synuclein regulate the amplification of pathological α-synuclein. *Nat. Neurosci.* **26**, 213–225 (2023).

10. J. Hu, W. Xia, S. Zeng, Y.-J. Lim, Y. Tao, Y. Sun, L. Zhao, H. Wang, W. Le, D. Li, S. Zhang, C. Liu, Y.-M. Li, Phosphorylation and O-GlcNAcylation at the same α-synuclein site generate distinct fibril structures. *Nat. Commun.* **15**, 2677 (2024).

11. J. Collinge, A. R. Clarke, A general model of prion strains and their pathogenicity. *Science* **318**, 930–936 (2007).

12. J. I. Ayers, J. Lee, O. Monteiro, A. L. Woerman, A. A. Lazar, C. Condello, N. A. Paras, S. B. Prusiner, Different α-synuclein prion strains cause dementia with Lewy bodies and multiple system atrophy. *Proc. Natl. Acad. Sci. U.S.A.* **119**, e2113489119 (2022).

13. K. C. Luk, V. Kehm, J. Carroll, B. Zhang, P. O'Brien, J. Q. Trojanowski, V. M.-Y. Lee, Pathological α-synuclein transmission initiates Parkinson-like neurodegeneration in nontransgenic mice. *Science* **338**, 949–953 (2012).

14. K. C. Luk, C. Song, P. O'Brien, A. Stieber, J. R. Branch, K. R. Brunden, J. Q. Trojanowski, V. M.-Y. Lee, Exogenous α-synuclein fibrils seed the formation of Lewy body-like intracellular inclusions in cultured cells. *Proc. Natl. Acad. Sci. U.S.A.* **106**, 20051–20056 (2009).

15. L. A. Volpicelli-Daley, K. C. Luk, V. M.-Y. Lee, Addition of exogenous α-synuclein preformed fibrils to primary neuronal cultures to seed recruitment of endogenous α-synuclein to Lewy body and Lewy neurite-like aggregates. *Nat. Protoc.* **9**, 2135–2146 (2014).

16. E. Gazit, The "Correctly Folded" state of proteins: Is it a metastable state? *Angew. Chem. Int. Ed. Engl.* **41**, 257–259 (2002).

17. K. Zhao, Y.-J. Lim, Z. Liu, H. Long, Y. Sun, J.-J. Hu, C. Zhao, Y. Tao, X. Zhang, D. Li, Y.-M. Li, C. Liu, Parkinson's disease-related phosphorylation at Tyr39 rearranges α-synuclein amyloid fibril structure revealed by cryo-EM. *Proc. Natl. Acad. Sci. U.S.A.* **117**, 20305–20315 (2020).

18. R. Guerrero-Ferreira, N. M. Taylor, D. Mona, P. Ringler, M. E. Lauer, R. Riek, M. Britschgi, H. Stahlberg, Cryo-EM structure of alpha-synuclein fibrils. *eLife* **7**, e36402 (2018).

19. S. Zhang, K. Dauer, T. Strohäker, L. Tatenhorst, L. Caldi Gomes, S. Mayer, B. C. Jung, W. S. Kim, S.-J. Lee, S. Becker, F. Liesche-Starnecker, M. Zweckstetter, P. Lingor, Alpha-synuclein fibrils amplified from multiple system atrophy and Parkinson's disease patient brain spread after intracerebral injection into mouse brain. *Brain Pathol.* **33**, e13196 (2023).

20. Y. Fan, Y. Sun, W. Yu, Y. Tao, W. Xia, Y. Liu, Q. Zhao, Y. Tang, Y. Sun, F. Liu, Q. Cao, J. Wu, C. Liu, J. Wang, D. Li, Conformational change of α-synuclein fibrils in cerebrospinal fluid from different clinical phases of Parkinson's disease. *Structure* **31**, 78–87.e5 (2023).

21. R. Guerrero-Ferreira, N. M. Taylor, A.-A. Arteni, P. Kumari, D. Mona, P. Ringler, M. Britschgi, M. E. Lauer, A. Makky, J. Verasdonck, R. Riek, R. Melki, B. H. Meier, A. Böckmann, L. Bousset, H. Stahlberg, Two new polymorphic structures of human full-length alpha-synuclein fibrils solved by cryo-electron microscopy. *eLife* **8**, e48907 (2019).

22. D. D. Dhavale, A. M. Barclay, C. G. Borcik, K. Basore, D. A. Berthold, I. R. Gordon, J. Liu, M. H. Milchberg, J. Y. O'Shea, M. J. Rau, Z. Smith, S. Sen, B. Summers, J. Smith, O. A. Warmuth, R. J. Perrin, J. S. Perlmutter, Q. Chen, J. A. J. Fitzpatrick, C. D. Schwieters, E. Tajkhorshid, C. M. Rienstra, P. T. Kotzbauer, Structure of alpha-synuclein fibrils derived from human Lewy body dementia tissue. *Nat. Commun.* **15**, 2750 (2024).

23. S. Lövestam, M. Schweighauser, T. Matsubara, S. Murayama, T. Tomita, T. Ando, K. Hasegawa, M. Yoshida, A. Tarutani, M. Hasegawa, M. Goedert, S. H. W. Scheres, Seeded assembly in vitro does not replicate the structures of α-synuclein filaments from multiple system atrophy. *FEBS Open Bio.* **11**, 999–1013 (2021).

24. B. Frieg, J. A. Geraets, T. Strohäker, C. Dienemann, P. Mavroeidi, B. C. Jung, W. S. Kim, S.-J. Lee, M. Xilouri, M. Zweckstetter, G. F. Schröder, Quaternary structure of patient-homogenate amplified α-synuclein fibrils modulates seeding of endogenous α-synuclein. *Commun. Biol.* **5**, 1040 (2022).

25. D. R. Boyer, B. Li, C. Sun, W. Fan, K. Zhou, M. P. Hughes, M. R. Sawaya, L. Jiang, D. S. Eisenberg, The α-synuclein hereditary mutation E46K unlocks a more stable, pathogenic fibril structure. *Proc. Natl. Acad. Sci. U.S.A.* **117**, 3592–3602 (2020).

26. K. C. Luk, V. M. Kehm, B. Zhang, P. O'Brien, J. Q. Trojanowski, V. M. Y. Lee, Intracerebral inoculation of pathological α-synuclein initiates a rapidly progressive neurodegenerative α-synucleinopathy in mice. *J. Exp. Med.* **209**, 975–986 (2012).

27. Z. A. Sorrentino, M. M. T. Brooks, V. Hudson III, N. J. Rutherford, T. E. Golde, B. I. Giasson, P. Chakrabarty, Intrastriatal injection of α-synuclein can lead to widespread synucleinopathy independent of neuroanatomic connectivity. *Mol. Neurodegener.* **12**, 40 (2017).

28. A. Shimozawa, M. Ono, D. Takahara, A. Tarutani, S. Imura, M. Masuda-Suzukake, M. Higuchi, K. Yanai, S.-I. Hisanaga, M. Hasegawa, Propagation of pathological α-synuclein in marmoset brain. *Acta Neuropathol. Commun.* **5**, 12 (2017).

29. A.-L. Mahul-Mellier, J. Burtscher, N. Maharjan, L. Weerens, M. Croisier, F. Kuttler, M. Leleu, G. W. Knott, H. A. Lashuel, The process of Lewy body formation, rather than simply α-synuclein fibrillization, is one of the major drivers of neurodegeneration. *Proc. Natl. Acad. Sci. U.S.A.* **117**, 4971–4982 (2020).

30. S. T. Kumar, A.-L. Mahul-Mellier, R. N. Hegde, G. Rivière, R. Moons, A. Ibáñez de Opakua, P. Magalhães, I. Rostami, S. Donzelli, F. Sobott, M. Zweckstetter, H. A. Lashuel, A NAC domain mutation (E83Q) unlocks the pathogenicity of human alpha-synuclein and recapitulates its pathological diversity. *Sci. Adv.* **8**, eabn0044 (2022).

31. I. Lam, A. Ndayisaba, A. J. Lewis, Y. Fu, G. T. Sagredo, L. Zaccagnini, J. Sandoe, R. L. Sanz, A. Vahdatshoar, T. D. Martin, N. Morshed, T. Ichihashi, A. Tripathi, N. Ramalingam, C. Oettgen-Suazo, T. Bartels, M. Schäbinger, E. Hallacli, X. Jiang, A. Verma, C. Tea, Z. Wang,

H. Hakozaki, X. Yu, K. Hyles, C. Park, T. W. Theunissen, H. Wang, R. Jaenisch, S. Lindquist, B. Stevens, N. Stefanova, G. Wenning, K. C. Luk, R. S. Pernaute, J. C. Gómez-Esteban, D. Felsky, Y. Kiyota, N. Sahni, S. S. Yi, C.-Y. Chung, H. Stahlberg, I. Ferrer, J. Schöneberg, S. J. Elledge, U. Dettmer, G. M. Halliday, T. Bartels, V. Khurana, Rapid iPSC inclusionopathy models shed light on formation, consequence, and molecular subtype of α-synuclein inclusions. *Neuron* **112**, 2886–2909 (2024).

32. A. Bayati, R. Ayoubi, A. Aguila, C. E. Zorca, C. Han, E. Banks, E. Nguyen-Renou, W. Luo, I. Shlaifer, E. Del Cid-Pellitero, M. Yaqubi, E. A. Fon, J. A. Stratton, T. M. Durcan, P. C. Nahirney, P. S. McPherson, A dual hit of α-synuclein internalization and immune challenge leads to the formation and maintenance of Lewy body-like inclusions in human dopaminergic neurons, bioRxiv 542776 [Preprint] (2023). https://doi.org/10.1101/2023.05.29.542776.

33. K. L. Paumier, K. C. Luk, F. P. Manfredsson, N. M. Kanaan, J. W. Lipton, T. J. Collier, K. Steece-Collier, C. J. Kemp, S. Celano, E. Schulz, I. M. Sandoval, S. Fleming, E. Dirr, N. K. Polinski, J. Q. Trojanowski, V. M. Lee, C. E. Sortwell, Intrastriatal injection of pre-formed mouse α-synuclein fibrils into rats triggers α-synuclein pathology and bilateral nigrostriatal degeneration. *Neurobiol. Dis.* **82**, 185–199 (2015).

34. Y. Chu, S. Muller, A. Tavares, O. Barret, D. Alagille, J. Seibyl, G. Tamagnan, K. Marek, K. C. Luk, J. Q. Trojanowski, V. M. Y. Lee, J. H. Kordower, Intrastriatal alpha-synuclein fibrils in monkeys: Spreading, imaging and neuropathological changes. *Brain* **142**, 3565–3579 (2019).

35. K. C. Luk, D. J. Covell, V. M. Kehm, B. Zhang, I. Y. Song, M. D. Byrne, R. M. Pitkin, S. C. Decker, J. Q. Trojanowski, V. M.-Y. Lee, Molecular and biological compatibility with host alpha-synuclein influences fibril pathogenicity. *Cell Rep.* **16**, 3373–3387 (2016).

36. N. L. Rey, S. George, J. A. Steiner, Z. Madaj, K. C. Luk, J. Q. Trojanowski, V. M.-Y. Lee, P. Brundin, Spread of aggregates after olfactory bulb injection of α-synuclein fibrils is associated with early neuronal loss and is reduced long term. *Acta Neuropathol.* **135**, 65–83 (2018).

37. N. L. Rey, J. A. Steiner, N. Maroof, K. C. Luk, Z. Madaj, J. Q. Trojanowski, V. M.-Y. Lee, P. Brundin, Widespread transneuronal propagation of α-synucleinopathy triggered in olfactory bulb mimics prodromal Parkinson's disease. *J. Exp. Med.* **213**, 1759–1778 (2016).

38. M. Masuda-Suzukake, T. Nonaka, M. Hosokawa, T. Oikawa, T. Arai, H. Akiyama, D. M. A. Mann, M. Hasegawa, Prion-like spreading of pathological α-synuclein in brain. *Brain* **136**, 1128–1138 (2013).

39. M. Masuda-Suzukake, T. Nonaka, M. Hosokawa, M. Kubo, A. Shimozawa, H. Akiyama, M. Hasegawa, Pathological alpha-synuclein propagates through neural networks. *Acta Neuropathol. Commun.* **2**, 88 (2014).

40. M.-B. Fares, B. Maco, A. Oueslati, E. Rockenstein, N. Ninkina, V. L. Buchman, E. Masliah, H. A. Lashuel, Induction of de novo α-synuclein fibrillization in a neuronal model for Parkinson's disease. *Proc. Natl. Acad. Sci. U.S.A.* **113**, E912–E921 (2016).

41. M. X. Henderson, E. J. Cornblath, A. Darwich, B. Zhang, H. Brown, R. J. Gathagan, R. M. Sandler, D. S. Bassett, J. Q. Trojanowski, V. M. Y. Lee, Spread of α-synuclein pathology through the brain connectome is modulated by selective vulnerability and predicted by network analysis. *Nat. Neurosci.* **22**, 1248–1257 (2019).

42. J. Burtscher, J.-C. Copin, C. Sandi, H. A. Lashuel, Pronounced α-synuclein pathology in a seeding-based mouse model is not sufficient to induce mitochondrial respiration deficits in the striatum and amygdala. *eNeuro* **7**, ENEURO.0110-20.2020 (2020).

43. D. J. Apicco, E. Shlevkov, C. L. Nezich, D. T. Tran, E. Guilmette, J. W. Nicholatos, C. M. Bantle, Y. Chen, K. E. Glajch, N. A. Abraham, L. T. Dang, G. C. Kaynor, E. A. Tsai, K.-D. H. Nguyen, J. Groot, Y. Liu, A. Weihofen, J. A. Hurt, H. Runz, W. D. Hirst, The Parkinson's disease-associated gene ITPKB protects against α-synuclein aggregation by regulating ER-to-mitochondria calcium release. *Proc. Natl. Acad. Sci. U.S.A.* **118**, e2006476118 (2021).

44. N. K. Polinski, L. A. Volpicelli-Daley, C. E. Sortwell, K. C. Luk, N. Cremades, L. M. Gottler, J. Froula, M. F. Duffy, V. M. Y. Lee, T. N. Martinez, K. D. Dave, Best practices for generating

and using alpha-synuclein pre-formed fibrils to model Parkinson's disease in rodents. *J. Parkinsons Dis.* **8**, 303–322 (2018).

45. T.-I. Kam, H. Park, S.-C. Chou, J. G. Van Vranken, M. J. Mittenbühler, H. Kim, A. Mu, Y. R. Choi, D. Biswas, J. Wang, Y. Shin, A. Loder, S. S. Karuppagounder, C. D. Wrann, V. L. Dawson, B. M. Spiegelman, T. M. Dawson, Amelioration of pathologic α-synuclein-induced Parkinson's disease by irisin. *Proc. Natl. Acad. Sci. U.S.A.* **119**, e2204835119 (2022).

46. H. Abdelmotilib, T. Maltbie, V. Delic, Z. Liu, X. Hu, K. B. Fraser, M. S. Moehle, L. Stoyka, N. Anabtawi, V. Krendelchtchikova, L. A. Volpicelli-Daley, A. West, α-Synuclein fibril-induced inclusion spread in rats and mice correlates with dopaminergic Neurodegeneration. *Neurobiol. Dis.* **105**, 84–98 (2017).

47. L. Kang, K.-P. Wu, M. Vendruscolo, J. Baum, The A53T mutation is key in defining the differences in the aggregation kinetics of human and mouse α-synuclein. *J. Am. Chem. Soc.* **133**, 13465–13470 (2011).

48. N. Landeck, K. E. Strathearn, D. Ysselstein, K. Buck, S. Dutta, S. Banerjee, Z. Lv, J. D. Hulleman, J. Hindupur, L.-K. Lin, S. Padalkar, L. A. Stanciu, Y. L. Lyubchenko, D. Kirik, J.-C. Rochet, Two C-terminal sequence variations determine differential neurotoxicity between human and mouse α-synuclein. *Mol. Neurodegener.* **15**, 49 (2020).

49. C. Lavedan, The synuclein family. *Genome Res.* **8**, 871–880 (1998).

50. J. W. Touchman, A. Dehejia, O. Chiba-Falek, D. E. Cabin, J. R. Schwartz, B. M. Orrison, M. H. Polymeropoulos, R. L. Nussbaum, Human and mouse α-synuclein genes: Comparative genomic sequence analysis and identification of a novel gene regulatory element. *Genome Res.* **11**, 78–86 (2001).

51. T. Ohgita, H. Kono, I. Morita, H. Oyama, T. Shimanouchi, N. Kobayashi, H. Saito, Intramolecular interaction kinetically regulates fibril formation by human and mouse α-synuclein. *Sci. Rep.* **13**, 10885 (2023).

52. S. Hwang, P. Fricke, M. Zinke, K. Giller, J. S. Wall, D. Riedel, S. Becker, A. Lange, Comparison of the 3D structures of mouse and human α-synuclein fibrils by solid-state NMR and STEM. *J. Struct. Biol.* **206**, 43–48 (2019).

53. M. Shahnawaz, A. Mukherjee, S. Pritzkow, N. Mendez, P. Rabadia, X. Liu, B. Hu, A. Schmeichel, W. Singer, G. Wu, A.-L. Tsai, H. Shirani, K. P. R. Nilsson, P. A. Low, C. Soto, Discriminating α-synuclein strains in Parkinson's disease and multiple system atrophy. *Nature* **578**, 273–277 (2020).

54. T. Strohäker, B. C. Jung, S.-H. Liou, C. O. Fernandez, D. Riedel, S. Becker, G. M. Halliday, M. Bennati, W. S. Kim, S.-J. Lee, M. Zweckstetter, Structural heterogeneity of α-synuclein fibrils amplified from patient brain extracts. *Nat. Commun.* **10**, 5535 (2019).

55. F. De Giorgi, F. Laferrière, F. Zinghirino, E. Faggiani, A. Lends, M. Bertoni, X. Yu, A. Grélard, E. Morvan, B. Habenstein, N. Dutheil, E. Doudnikoff, J. Daniel, S. Claverol, C. Qin, A. Loquet, E. Bezard, F. Ichas, Novel self-replicating α-synuclein polymorphs that escape ThT monitoring can spontaneously emerge and acutely spread in neurons. *Sci. Adv.* **6**, eabc4364 (2020).

56. P. Greenspan, S. D. Fowler, Spectrofluorometric studies of the lipid probe, nile red. *J. Lipid Res.* **26**, 781–789 (1985).

57. M. N. Bongiovanni, J. Godet, M. H. Horrocks, L. Tosatto, A. R. Carr, D. C. Wirthensohn, R. T. Ranasinghe, J.-E. Lee, A. Ponjavic, J. V. Fritz, C. M. Dobson, D. Klenerman, S. F. Lee, Multi-dimensional super-resolution imaging enables surface hydrophobicity mapping. *Nat. Commun.* **7**, 13544 (2016).

58. M. R. Sawaya, M. P. Hughes, J. A. Rodriguez, R. Riek, D. S. Eisenberg, The expanding amyloid family: Structure, stability, function, and pathogenesis. *Cell* **184**, 4857–4873 (2021).

59. L. Bousset, P. Brundin, A. Böckmann, B. Meier, R. Melki, An efficient procedure for removal and inactivation of alpha-synuclein assemblies from laboratory materials. *J. Parkinsons Dis.* **6**, 143–151 (2016).

60. L. A. Volpicelli-Daley, K. C. Luk, T. P. Patel, S. A. Tanik, D. M. Riddle, A. Stieber, D. F. Meaney, J. Q. Trojanowski, V. M.-Y. Lee, Exogenous α-synuclein fibrils induce Lewy body pathology leading to synaptic dysfunction and neuron death. *Neuron* **72**, 57–71 (2011).

61. H. Gram, V. Theologidis, T. Boesen, P. H. Jensen, Sarkosyl differentially solubilizes patient-derived alpha-synuclein fibril strains. *Front. Mol. Biosci.* **10**, 1177556 (2023).

62. J. M. Froula, M. Castellana-Cruz, N. M. Anabtawi, J. D. Camino, S. W. Chen, D. R. Thrasher, J. Freire, A. A. Yazdi, S. Fleming, C. M. Dobson, J. R. Kumita, N. Cremades, L. A. Volpicelli-Daley, Defining α-synuclein species responsible for Parkinson's disease phenotypes in mice. *J. Biol. Chem.* **294**, 10392–10406 (2019).

63. A. Tarutani, G. Suzuki, A. Shimozawa, T. Nonaka, H. Akiyama, S.-I. Hisanaga, M. Hasegawa, The effect of fragmented pathogenic α-synuclein seeds on prion-like propagation. *J. Biol. Chem.* **291**, 18675–18688 (2016).

64. A. Sakunthala, D. Datta, A. Navalkar, L. Gadhe, P. Kadu, K. Patel, S. Mehra, R. Kumar, D. Chatterjee, J. Devi, K. Sengupta, R. Padinhateeri, S. K. Maji, Direct demonstration of seed size-dependent α-synuclein amyloid amplification. *J. Phys. Chem. Lett.* **13**, 6427–6438 (2022).

65. D. M. Beal, M. Tournus, R. Marchante, T. J. Purton, D. P. Smith, M. F. Tuite, M. Doumic, W.-F. Xue, The Division of amyloid fibrils: Systematic comparison of fibril fragmentation stability by linking theory with experiments. *iScience* **23**, 101512 (2020).

66. W.-F. Xue, A. L. Hellewell, E. W. Hewitt, S. E. Radford, Fibril fragmentation in amyloid assembly and cytotoxicity: When size matters. *Prion* **4**, 20–25 (2010).

67. M. Saiki, S. Honda, K. Kawasaki, D. Zhou, A. Kaito, T. Konakahara, H. Morii, Higher-order molecular packing in amyloid-like fibrils constructed with linear arrangements of hydrophobic and hydrogen-bonding side-chains. *J. Mol. Biol.* **348**, 983–998 (2005).

68. A. Sokratian, J. Ziaee, K. Kelly, A. Chang, N. Bryant, S. Wang, E. Xu, J. Y. Li, S.-H. Wang, J. Ervin, S. M. Swain, R. A. Liddle, A. B. West, Heterogeneity in α-synuclein fibril activity

correlates to disease phenotypes in Lewy body dementia. *Acta Neuropathol.* **141**, 547–564 (2021).

69. J. W. Howe, C. E. Sortwell, M. F. Duffy, C. J. Kemp, C. P. Russell, M. Kubik, P. Patel, K. C. Luk, O. M. A. El-Agnaf, J. R. Patterson, Preformed fibrils generated from mouse alpha-synuclein produce more inclusion pathology in rats than fibrils generated from rat alpha-synuclein. *Parkinsonism Relat. Disord.* **89**, 41–47 (2021).

70. R. H. Earls, K. B. Menees, J. Chung, J. Barber, C.-A. Gutekunst, M. G. Hazim, J.-K. Lee, Intrastriatal injection of preformed alpha-synuclein fibrils alters central and peripheral immune cell profiles in non-transgenic mice. *J. Neuroinflammation* **16**, 250 (2019).

71. K. Saini, S. Cho, L. J. Dooling, D. E. Discher, Tension in fibrils suppresses their enzymatic degradation—A molecular mechanism for 'use it or lose it'. *Matrix Biol.* **85-86**, 34–46 (2020).

72. M. I. Sulatsky, A. I. Sulatskaya, O. V. Stepanenko, O. I. Povarova, I. M. Kuznetsova, K. K. Turoverov, Denaturant effect on amyloid fibrils: Declasterization, depolymerization, denaturation and reassembly. *Int. J. Biol. Macromol.* **150**, 681–694 (2020).

73. D. S. Davidson, A. M. Brown, J. A. Lemkul, Insights into stabilizing forces in amyloid fibrils of differing sizes from polarizable molecular dynamics simulations. *J. Mol. Biol.* **430**, 3819–3834 (2018).

74. V. Grozdanov, L. Bousset, M. Hoffmeister, C. Bliederhaeuser, C. Meier, K. Madiona, L. Pieri, M. Kiechle, P. J. McLean, J. Kassubek, C. Behrends, A. C. Ludolph, J. H. Weishaupt, R. Melki, K. M. Danzer, Increased immune activation by pathologic α-synuclein in Parkinson's disease. *Ann. Neurol.* **86**, 593–606 (2019).

75. A. S. Harms, V. Delic, A. D. Thome, N. Bryant, Z. Liu, S. Chandra, A. Jurkuvenaite, A. B. West, α-Synuclein fibrils recruit peripheral immune cells in the rat brain prior to neurodegeneration. *Acta Neuropathol. Commun.* **5**, 85 (2017).

76. E. Xu, R. Boddu, H. A. Abdelmotilib, A. Sokratian, K. Kelly, Z. Liu, N. Bryant, S. Chandra, S. M. Carlisle, E. J. Lefkowitz, A. S. Harms, E. N. Benveniste, T. A. Yacoubian, L. A. Volpicelli-Daley, D. G. Standaert, A. B. West, Pathological α-synuclein recruits LRRK2 expressing pro-inflammatory monocytes to the brain. *Mol. Neurodegener.* **17**, 7 (2022).

77. F. Hu, D. Song, Y. Yan, C. Huang, C. Shen, J. Lan, Y. Chen, A. Liu, Q. Wu, L. Sun, F. Xu, F. Hu, L. Chen, X. Luo, Y. Feng, S. Huang, J. Hu, G. Wang, IL-6 regulates autophagy and chemotherapy resistance by promoting BECN1 phosphorylation. *Nat. Commun.* **12**, 3651 (2021).

78. P. Jiang, M. Gan, S.-H. Yen, P. J. McLean, D. W. Dickson, Impaired endo-lysosomal membrane integrity accelerates the seeding progression of α-synuclein aggregates. *Sci. Rep.* **7**, 7690 (2017).

79. A. M. Silverman, R. Nakata, H. Shimada, R. Sposto, Y. A. DeClerck, A galectin-3-dependent pathway upregulates interleukin-6 in the microenvironment of human neuroblastoma. *Cancer Res.* **72**, 2228–2238 (2012).

80. C.-H. Tang, C.-J. Hsu, Y.-C. Fong, The CCL5/CCR5 axis promotes interleukin-6 production in human synovial fibroblasts. *Arthritis Rheum.* **62**, 3615–3624 (2010).

81. Z. Liu, A. Sokratian, A. M. Duda, E. Xu, C. Stanhope, A. Fu, S. Strader, H. Li, Y. Yuan, B. G. Bobay, J. Sipe, K. Bai, I. Lundgaard, N. Liu, B. Hernandez, C. Bowes Rickman, S. E. Miller, A. B. West, Anionic nanoplastic contaminants promote Parkinson's disease-associated α-synuclein aggregation. *Sci. Adv.* **9**, eadi8716 (2023).

82. D. Freeman, R. Cedillos, S. Choyke, Z. Lukic, K. McGuire, S. Marvin, A. M. Burrage, S. Sudholt, A. Rana, C. O'Connor, C. M. Wiethoff, E. M. Campbell, Alpha-synuclein induces lysosomal rupture and cathepsin dependent reactive oxygen species following endocytosis. *PLOS ONE* **8**, e62143 (2013).

83. A. Bayati, E. Banks, C. Han, W. Luo, W. E. Reintsch, C. E. Zorca, I. Shlaifer, E. Del Cid Pellitero, B. Vanderperre, H. M. McBride, E. A. Fon, T. M. Durcan, P. S. McPherson, Rapid macropinocytic transfer of α-synuclein to lysosomes. *Cell Rep.* **40**, 111102 (2022).

84. N. P. Marotta, J. Ara, N. Uemura, M. G. Lougee, E. S. Meymand, B. Zhang, E. J. Petersson, J. Q. Trojanowski, V. M.-Y. Lee, Alpha-synuclein from patient Lewy bodies exhibits distinct pathological activity that can be propagated in vitro. *Acta Neuropathol. Commun.* **9**, 188 (2021).

85. J. R. Mazzulli, F. Zunke, O. Isacson, L. Studer, D. Krainc, α-Synuclein-induced lysosomal dysfunction occurs through disruptions in protein trafficking in human midbrain synucleinopathy models. *Proc. Natl. Acad. Sci. U.S.A.* **113**, 1931–1936 (2016).

86. M. F. Duffy, T. J. Collier, J. R. Patterson, C. J. Kemp, K. C. Luk, M. G. Tansey, K. L. Paumier, N. M. Kanaan, D. L. Fischer, N. K. Polinski, O. L. Barth, J. W. Howe, N. N. Vaikath, N. K. Majbour, O. M. A. El-Agnaf, C. E. Sortwell, Lewy body-like alpha-synuclein inclusions trigger reactive microgliosis prior to nigral degeneration. *J. Neuroinflammation* **15**, 129 (2018).

87. A. T. Balana, A.-L. Mahul-Mellier, B. A. Nguyen, M. Horvath, A. Javed, E. R. Hard, Y. Jasiqi, P. Singh, S. Afrin, R. Pedretti, V. Singh, V. M.-Y. Lee, K. C. Luk, L. Saelices, H. A. Lashuel, M. R. Pratt, O-GlcNAc forces an α-synuclein amyloid strain with notably diminished seeding and pathology. *Nat. Chem. Biol.* **20**, 646–655 (2024).

88. V. Delic, S. Chandra, H. Abdelmotilib, T. Maltbie, S. Wang, D. Kem, H. J. Scott, R. N. Underwood, Z. Liu, L. A. Volpicelli-Daley, A. B. West, Sensitivity and specificity of phospho-Ser129 α-synuclein monoclonal antibodies. *J. Comp. Neurol.* **526**, 1978–1990 (2018).

89. H. T. Tran, C. H.-Y. Chung, M. Iba, B. Zhang, J. Q. Trojanowski, K. C. Luk, V. M. Y. Lee, α-Synuclein immunotherapy blocks uptake and templated propagation of misfolded α-synuclein and neurodegeneration. *Cell Rep.* **7**, 2054–2065 (2014).

90. M. X. Henderson, D. J. Covell, C. H.-Y. Chung, R. M. Pitkin, R. M. Sandler, S. C. Decker, D. M. Riddle, B. Zhang, R. J. Gathagan, M. J. James, J. Q. Trojanowski, K. R. Brunden, V.

M. Y. Lee, K. C. Luk, Characterization of novel conformation-selective α-synuclein antibodies as potential immunotherapeutic agents for Parkinson's disease. *Neurobiol. Dis.* **136**, 104712 (2020).

91. A. Weihofen, Y. Liu, J. W. Arndt, C. Huy, C. Quan, B. A. Smith, J.-L. Baeriswyl, N. Cavegn, L. Senn, L. Su, G. Marsh, P. K. Auluck, F. Montrasio, R. M. Nitsch, W. D. Hirst, J. M. Cedarbaum, R. B. Pepinsky, J. Grimm, P. H. Weinreb, Development of an aggregate-selective, human-derived α-synuclein antibody BIIB054 that ameliorates disease phenotypes in Parkinson's disease models. *Neurobiol. Dis.* **124**, 276–288 (2019).

92. M. A. G. Gilbert, N. Fatima, J. Jenkins, T. J. O'Sullivan, A. Schertel, Y. Halfon, M. Wilkinson, T. H. J. Morrema, M. Geibel, R. J. Read, N. A. Ranson, S. E. Radford, J. J. M. Hoozemans, R. A. W. Frank, CryoET of β-amyloid and tau within postmortem Alzheimer's disease brain. *Nature* **631**, 913–919 (2024).

93. D. Sulzer, R. N. Alcalay, F. Garretti, L. Cote, E. Kanter, J. Agin-Liebes, C. Liong, C. McMurtrey, W. H. Hildebrand, X. Mao, V. L. Dawson, T. M. Dawson, C. Oseroff, J. Pham, J. Sidney, M. B. Dillon, C. Carpenter, D. Weiskopf, E. Phillips, S. Mallal, B. Peters, A. Frazier, C. S. Lindestam Arlehamn, A. Sette, T cells from patients with Parkinson's disease recognize α-synuclein peptides. *Nature* **546**, 656–661 (2017).

94. V. Sanchez-Guajardo, F. Febbraro, D. Kirik, M. Romero-Ramos, Microglia acquire distinct activation profiles depending on the degree of alpha-synuclein neuropathology in a rAAV based model of Parkinson's disease. *PLOS ONE* **5**, e8784 (2010).

95. A. A. Dijkstra, A. Ingrassia, R. X. de Menezes, R. E. van Kesteren, A. J. M. Rozemuller, P. Heutink, W. D. J. van de Berg, Evidence for immune response, axonal dysfunction and reduced endocytosis in the substantia nigra in early stage Parkinson's disease. *PLOS ONE* **10**, e0128651 (2015).

96. H. Asai, S. Ikezu, S. Tsunoda, M. Medalla, J. Luebke, T. Haydar, B. Wolozin, O. Butovsky, S. Kügler, T. Ikezu, Depletion of microglia and inhibition of exosome synthesis halt tau propagation. *Nat. Neurosci.* **18**, 1584–1593 (2015).

97. I. Dalgediene, R. Lasickiene, R. Budvytyte, G. Valincius, R. Morkuniene, V. Borutaite, A. Zvirbliene, Immunogenic properties of amyloid beta oligomers. *J. Biomed. Sci.* **20**, 10 (2013).

98. C. D. Hughes, M. L. Choi, M. Ryten, L. Hopkins, A. Drews, J. A. Botía, M. Iljina, M. Rodrigues, S. A. Gagliano, S. Gandhi, C. Bryant, D. Klenerman, Picomolar concentrations of oligomeric alpha-synuclein sensitizes TLR4 to play an initiating role in Parkinson's disease pathogenesis. *Acta Neuropathol.* **137**, 103–120 (2019).

99. J. Jia, A. Claude-Taupin, Y. Gu, S. W. Choi, R. Peters, B. Bissa, M. H. Mudd, L. Allers, S. Pallikkuth, K. A. Lidke, M. Salemi, B. Phinney, M. Mari, F. Reggiori, V. Deretic, Galectin-3 coordinates a cellular system for lysosomal repair and removal. *Dev. Cell* **52**, 69–87.e8 (2020).

100. T. Otomo, T. Yoshimori, Lysophagy: A method for monitoring lysosomal rupture followed by autophagy-dependent recovery. *Methods Mol. Biol.* **1594**, 141–149 (2017).

101. C. Papadopoulos, H. Meyer, Detection and clearance of damaged lysosomes by the endo-lysosomal damage response and lysophagy. *Curr. Biol.* **27**, R1330–R1341 (2017).

102. M. A. Burguillos, M. Svensson, T. Schulte, A. Boza-Serrano, A. Garcia-Quintanilla, E. Kavanagh, M. Santiago, N. Viceconte, M. J. Oliva-Martin, A. M. Osman, E. Salomonsson, L. Amar, A. Persson, K. Blomgren, A. Achour, E. Englund, H. Leffler, J. L. Venero, B. Joseph, T. Deierborg, Microglia-secreted galectin-3 acts as a Toll-like receptor 4 ligand and contributes to microglial activation. *Cell Rep.* **10**, 1626–1638 (2015).

103. C. Peng, R. J. Gathagan, D. J. Covell, C. Medellin, A. Stieber, J. L. Robinson, B. Zhang, R. M. Pitkin, M. F. Olufemi, K. C. Luk, J. Q. Trojanowski, V. M.-Y. Lee, Cellular milieu imparts distinct pathological α-synuclein strains in α-synucleinopathies. *Nature* **557**, 558–563 (2018).

104. S. Donzelli, S. A. OSullivan, A.-L. Mahul-Mellier, A. Ulusoy, G. Fusco, S. T. Kumar, A. Chiki, J. Burtscher, M. L. D. Boussouf, I. Rostami, A. D. Simone, D. A. Di Monte, H. A. Lashuel, Post-fibrillization nitration of alpha-synuclein abolishes its seeding activity and

pathology formation in primary neurons and in vivo. bioRxiv 534149 [Preprint] (2023). https://doi.org/10.1101/2023.03.24.534149.

105. I. Martinez-Valbuena, N. P. Visanji, A. Kim, H. H. C. Lau, R. W. L. So, S. Alshimemeri, A. Gao, M. A. Seidman, M. R. Luquin, J. C. Watts, A. E. Lang, G. G. Kovacs, Alpha-synuclein seeding shows a wide heterogeneity in multiple system atrophy. *Transl. Neurodegener.* **11**, 7 (2022).

106. A. Van der Perren, G. Gelders, A. Fenyi, L. Bousset, F. Brito, W. Peelaerts, C. Van den Haute, S. Gentleman, R. Melki, V. Baekelandt, The structural differences between patient-derived α-synuclein strains dictate characteristics of Parkinson's disease, multiple system atrophy and dementia with Lewy bodies. *Acta Neuropathol.* **139**, 977–1000 (2020).

107. N. K. Polinski, A summary of phenotypes observed in the in vivo rodent alpha-synuclein preformed fibril model. *J. Parkinsons Dis.* **11**, 1555–1567 (2021).

108. H. Long, W. Zheng, Y. Liu, Y. Sun, K. Zhao, Z. Liu, W. Xia, S. Lv, Z. Liu, D. Li, K.-W. He, C. Liu, Wild-type α-synuclein inherits the structure and exacerbated neuropathology of E46K mutant fibril strain by cross-seeding. *Proc. Natl. Acad. Sci. U.S.A.* **118**, e2012435118 (2021).

109. D. N. Mastronarde, Automated electron microscope tomography using robust prediction of specimen movements. *J. Struct. Biol.* **152**, 36–51 (2005).

110. N. Biyani, R. D. Righetto, R. McLeod, D. Caujolle-Bert, D. Castano-Diez, K. N. Goldie, H. Stahlberg, Focus: The interface between data collection and data processing in cryo-EM. *J. Struct. Biol.* **198**, 124–133 (2017).

111. D. Kimanius, L. Dong, G. Sharov, T. Nakane, S. H. W. Scheres, New tools for automated cryo-EM single-particle analysis in RELION-4.0. *Biochem. J.* **478**, 4169–4185 (2021).

112. S. H. W. Scheres, RELION: Implementation of a Bayesian approach to cryo-EM structure determination. *J. Struct. Biol.* **180**, 519–530 (2012).

113. S. Q. Zheng, E. Palovcak, J.-P. Armache, K. A. Verba, Y. Cheng, D. A. Agard, MotionCor2: Anisotropic correction of beam-induced motion for improved cryo-electron microscopy. *Nat. Methods* **14**, 331–332 (2017).

114. A. Rohou, N. Grigorieff, CTFFIND4: Fast and accurate defocus estimation from electron micrographs. *J. Struct. Biol.* **192**, 216–221 (2015).

115. P. Emsley, K. Cowtan, Coot: Model-building tools for molecular graphics. *Acta Crystallogr. D Biol. Crystallogr.* **60**, 2126–2132 (2004).

116. V. B. Chen, W. B. Arendall III, J. J. Headd, D. A. Keedy, R. M. Immormino, G. J. Kapral, L. W. Murray, J. S. Richardson, D. C. Richardson, MolProbity: All-atom structure validation for macromolecular crystallography. *Acta Crystallogr. D Biol. Crystallogr.* **66**, 12–21 (2010).

117. P. D. Adams, P. V. Afonine, G. Bunkóczi, V. B. Chen, I. W. Davis, N. Echols, J. J. Headd, L.-W. Hung, G. J. Kapral, R. W. Grosse-Kunstleve, A. J. McCoy, N. W. Moriarty, R. Oeffner, R. J. Read, D. C. Richardson, J. S. Richardson, T. C. Terwilliger, P. H. Zwart, PHENIX: A comprehensive Python-based system for macromolecular structure solution. *Acta Crystallogr. D Biol. Crystallogr.* **66**, 213–221 (2010).

118. T. I. Croll, ISOLDE: A physically realistic environment for model building into low-resolution electron-density maps. *Acta Crystallogr. D Struct. Biol.* **74**, 519–530 (2018).

119. C. J. Williams, J. J. Headd, N. W. Moriarty, M. G. Prisant, L. L. Videau, L. N. Deis, V. Verma, D. A. Keedy, B. J. Hintze, V. B. Chen, S. Jain, S. M. Lewis, W. B. Arendall III, J. Snoeyink, P. D. Adams, S. C. Lovell, J. S. Richardson, D. C. Richardson, MolProbity: More and better reference data for improved all-atom structure validation. *Protein Sci.* **27**, 293–315 (2018).

120. T. D. Goddard, C. C. Huang, E. C. Meng, E. F. Pettersen, G. S. Couch, J. H. Morris, T. E. Ferrin, UCSF ChimeraX: Meeting modern challenges in visualization and analysis. *Protein Sci.* **27**, 14–25 (2018).

121. E. C. Meng, T. D. Goddard, E. F. Pettersen, G. S. Couch, Z. J. Pearson, J. H. Morris, T. E. Ferrin, UCSF ChimeraX: Tools for structure building and analysis. *Protein Sci.* **32**, e4792 (2023).

122. S. Mukherjee, Y. Zhang, MM-align: A quick algorithm for aligning multiple-chain protein complex structures using iterative dynamic programming. *Nucleic Acids Res.* **37**, e83 (2009).

123. S. Kriks, J.-W. Shim, J. Piao, Y. M. Ganat, D. R. Wakeman, Z. Xie, L. Carrillo-Reid, G. Auyeung, C. Antonacci, A. Buch, L. Yang, M. F. Beal, D. J. Surmeier, J. H. Kordower, V. Tabar, L. Studer, Dopamine neurons derived from human ES cells efficiently engraft in animal models of Parkinson's disease. *Nature* **480**, 547–551 (2011).

124. J. R. Mazzulli, F. Zunke, T. Tsunemi, N. J. Toker, S. Jeon, L. F. Burbulla, S. Patnaik, E. Sidransky, J. J. Marugan, C. M. Sue, D. Krainc, Activation of β-glucocerebrosidase reduces pathological α-synuclein and restores lysosomal function in Parkinson's patient midbrain neurons. *J. Neurosci.* **36**, 7693–7706 (2016).

125. Y. Tao, Y. Sun, S. Lv, W. Xia, K. Zhao, Q. Xu, Q. Zhao, L. He, W. Le, Y. Wang, C. Liu, D. Li, Heparin induces α-synuclein to form new fibril polymorphs with attenuated neuropathology. *Nat. Commun.* **13**, 4226 (2022).

126. C. Sun, K. Zhou, P. DePaola IV, W. S. Shin, T. Hillyer, M. R. Sawaya, R. Zhu, C. Peng, Z. H. Zhou, L. Jiang, Cryo-EM structure of amyloid fibril formed by α-synuclein hereditary A53E mutation reveals a distinct protofilament interface. *J. Biol. Chem.* **299**, 104566 (2023).

127. D. R. Boyer, B. Li, C. Sun, W. Fan, M. R. Sawaya, L. Jiang, D. S. Eisenberg, Structures of fibrils formed by α-synuclein hereditary disease mutant H50Q reveal new polymorphs. *Nat. Struct. Mol. Biol.* **26**, 1044–1052 (2019).

128. Y. Sun, H. Long, W. Xia, K. Wang, X. Zhang, B. Sun, Q. Cao, Y. Zhang, B. Dai, D. Li, C. Liu, The hereditary mutation G51D unlocks a distinct fibril strain transmissible to wild-type α-synuclein. *Nat. Commun.* **12**, 6252 (2021).

129. R. P. McGlinchey, X. Ni, J. A. Shadish, J. Jiang, J. C. Lee, The N terminus of α-synuclein dictates fibril formation. *Proc. Natl. Acad. Sci. U.S.A.* **118**, e2023487118 (2021).

130. Y. Tao, W. Xia, Q. Zhao, H. Xiang, C. Han, S. Zhang, W. Gu, W. Tang, Y. Li, L. Tan, D. Li, C. Liu, Structural mechanism for specific binding of chemical compounds to amyloid fibrils. *Nat. Chem. Biol.* **19**, 1235–1245 (2023).

131. B. Frieg, L. Antonschmidt, C. Dienemann, J. A. Geraets, E. E. Najbauer, D. Matthes, B. L. de Groot, L. B. Andreas, S. Becker, C. Griesinger, G. F. Schröder, The 3D structure of lipidic fibrils of α-synuclein. *Nat. Commun.* **13**, 6810 (2022).

132. B. Frieg, J. A. Geraets, T. Strohäker, C. Dienemann, P. Mavroeidi, B. C. Jung, W. S. Kim, S.-J. Lee, M. Xilouri, M. Zweckstetter, G. F. Schröder, α-Synuclein polymorphism determines oligodendroglial dysfunction. bioRxiv 451731 [Preprint] (2021). https://doi.org/10.1101/2021.07.09.451731.

133. A. Sokratian, Y. Zhou, E. Xu, E. Viverette, L. Dillard, Y. Yuan, J. Y. Li, A. Matarangas, J. Bouvette, M. Borgnia, A. Bartesaghi, A. West, Structural and functional landscape of α-synuclein fibril conformations amplified from cerebrospinal fluid. bioRxiv 499896 [Preprint] (2022). https://doi.org/10.1101/2022.07.13.499896.
